# Microhydration and Interfacial Activity of Triarylmethane Dyes at the Air–Water Interface

**DOI:** 10.1021/acs.jpcb.6c01352

**Published:** 2026-05-19

**Authors:** S. K. A. Ishini Madushika Sooriyabandara, Nikolay V. Tkachenko

**Affiliations:** † Department of Chemistry and Biochemistry, 6187 University of Oklahoma, Norman, Oklahoma 73019, United States; ‡ Materials Science and Engineering Program, University of Oklahoma, Norman, Oklahoma 73019, United States

## Abstract

Interfacial adsorption and aggregation of triarylmethane (TAM) dyes are central to their photophysics and to spectroscopic signals measured at the air–water interface. However, the molecular mechanisms controlling how chemical substitution modulates interfacial behavior remain underexplored. In this work, we report a multiscale computational study of the interfacial behavior of a series of TAM dye cations at the air–water interface. Our results demonstrate that TAM dyes exhibit strong interfacial affinity and adsorb through a two-step process: entering the interface region in a predominantly vertical orientation followed by reorientation into a surface-bound state, with a mostly planar orientation. Moreover, the preference for planar interfacial orientation becomes more pronounced as dye size increases. We further show that at higher concentrations dyes could form long-lived small aggregates that exhibit strong attractive interactions due to dispersion that can overcome Coulombic repulsion. We also show that the qualitative adsorption and orientational trends are robust across many classical water models and temperature regimes. Finally, ensemble-averaged TDDFT spectra computed from MD snapshots with explicit solvation indicate that bulk and interfacial spectra are similar under symmetric parametrization, whereas quinone-like parametrization yields non-negligible bulk-interface shifts.

## Graphical abstract

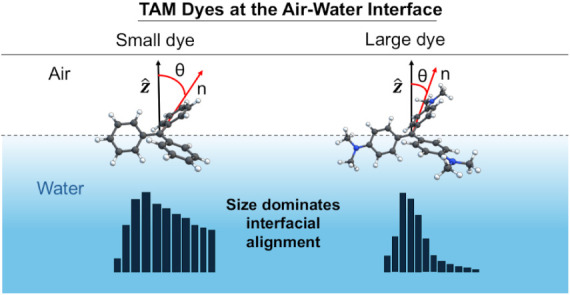

## 1 Introduction

The air–water interface is a structurally and chemically distinct environment compared to bulk water. Molecules near the interface experience anisotropic solvation, strong concentration gradients, and interfacial electric fields. Moreover, they can preferentially orient in ways that are disfavored or rarely sampled in fully solvated bulk environments.^1–3^ As a result, interfaces can shift both thermodynamic stability and kinetics, allowing for reaction pathways and physical behavior that are not observed (or much less favorable) in homogeneous bulk solutions and gas phase.^4–10^ Consistent with this view, experimental studies have shown that oxidation reactions can proceed at rates orders of magnitude faster at the air–water interface, demonstrating that interfaces work as distinct reactive environments.^3,6,11^ Moreover, different molecular orientations at the air–water interface can lead to large differences in chemical reactivity even when the same functional groups present.^12^ In particular, photochemical processes can be highly sensitive to solvation structure, interfacial electric fields, and orientational bias.^9,13^ Therefore, determining whether molecules accumulate at the interface and how they orient there is essential for understanding their photochemistry, optical absorption, and electron–nuclear couplings that control excited state dynamics.

A wide range of experimental techniques, including X-ray related techniques,^14^ vibrational spectroscopy,^15–17^ and surface-sensitive scattering,^18,19^ have been used to probe the air–water interface. These methods can provide surface-specific averaged observables (e.g., interfacial electron density profiles or orientational order parameters). However, they generally do not uniquely resolve each microscopic configuration contributing to the signal, nor its time-dependent fluctuations. Molecular dynamics (MD) simulations address this gap and provide a molecule-resolved picture of the interface, including structure and dynamics.

In this work we investigate a representative class of organic chromophores, triarylmethane (TAM) dyes. Their well-studied photophysical properties make them relevant to both applied systems and fundamental studies.^20–22^ TAM dyes are widely used as model analytes since their strong optical responses and adsorption to metallic nanostructured substrates.^23^ Their electronic structure and chemical tunability led to investigations of TAM derivatives in organic optoelectronics, including OLED emitters,^24,25^ as well as in nonlinear optical materials.^26,27^ Recently, structurally related triarylmethane and trityl-type radicals have also emerged as promising platforms for molecular spin qubits, exhibiting long spin coherence times and straightforward chemical functionalization, making them useful for quantum information processing and molecular spintronics.^28,29^ Furthermore, TAM dyes, including crystal violet and malachite green, are widely used chromophores for investigating ultrafast nonradiative relaxation processes. These pathways depend strongly on solvation, solvent viscosity, and conformational freedom.^30–39^ Thus, at the air–water interface malachite green exhibits excited state dynamics that differ from bulk^40–43^

Overall, the available data suggest that triarylmethane dyes can have substantial interfacial affinity arising from interplay between electrostatic hydration and dispersion interactions. However, most prior work focuses on individual dyes one at a time rather than a systematically substituted series, which makes it difficult to isolate how chemical modifications control interfacial behavior. In particular, systematic comparisons across amine substitution under the same air–water conditions, and trajectory-resolved molecular-level analyses from large-scale MD, remain limited. Here we address this gap with extensive molecular dynamics simulations of a structurally controlled series of triarylmethane dyes (Figure 1) at the air–water interface, which allows a direct comparison across different systems. In this work, we address the following questions: (1) whether TAM dyes exhibit preferential interfacial orientation at the air–water interface; (2) how aryl substitutions control interfacial behavior of TAM molecules; (3) whether adsorption follows a common reorientation pathway; (4) how sensitive structural observables are to atom type assignments and water model choice in classical force fields; (5) how roughness of water surface affects the molecule orientation; and (6) how conformational ensemble differences influence bulk-versus-interface TDDFT spectra.

**Figure 1:**
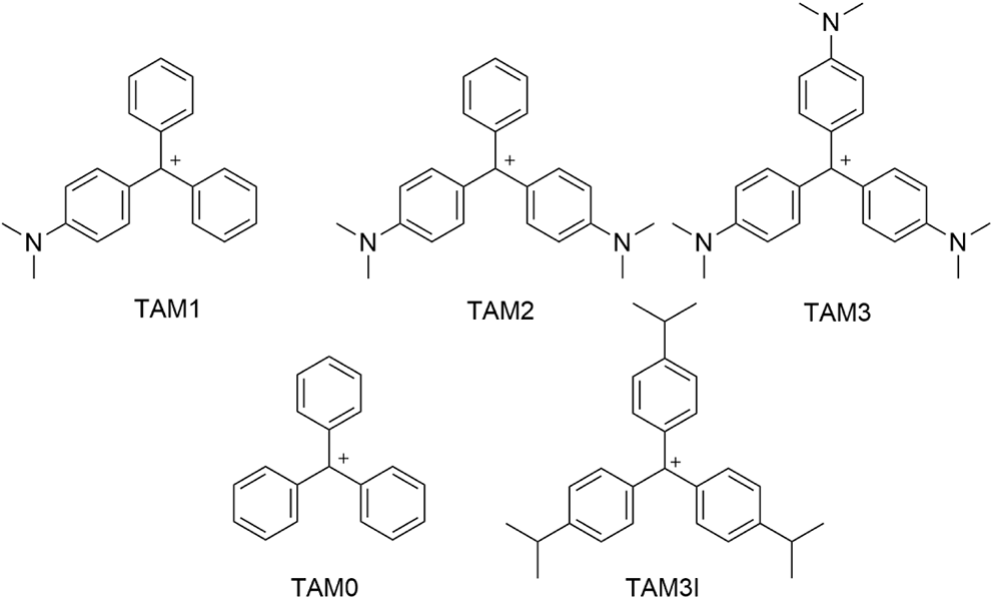
Chemical structures of the triarylmethane compounds considered in this work: unsubstituted triphenylmethane (TAM0), mono­(dimethylamino)-substituted analog (TAM1), malachite green (TAM2), crystal violet (TAM3), and the alkyl-substituted analog with three *p*-isopropyl groups (TAM3I).

## 2 Computational Methods

For brevity, we refer to the five molecules studied here as TAM0 (triphenylmethyl cation), TAM1 ((4-(dimethylamino)­phenyl)­diphenylmethyl cation), TAM2 (bis­(4-(dimethylamino)­phenyl)­(phenyl)­methyl cation), TAM3 (tris­(4-(dimethylamino)­phenyl)­methyl cation), and TAM3I (tris­(4-isopropylphenyl)­methyl cation) throughout the manuscript (Figure 1).

### 2.1 Electronic Structure Calculations

All electronic structure calculations were performed using the ORCA 6.1 software.^44^ Unless stated otherwise, TightSCF convergence criteria and the DEFGRID3 numerical integration grid were used to ensure numerical stability and convergence.

#### 2.1.1 Microhydration Analysis

To identify low-energy microhydrated structures of the dye cations, we used the DOCKER module in ORCA. This algorithm is based on particle swarm optimization (PSO)^45^ and has been shown to efficiently locate low-energy isomers. Initial structures of TAM0, TAM1, TAM2, and TAM3 were optimized at the GFN2-xTB level.^46^ Microhydrated clusters with 1–80 explicit water molecules were then generated.

#### 2.1.2 UV–Vis Spectra Calculations

To quantify how the solvation environment affects the UV–vis spectra, we performed TDDFT calculations at the ωB97X/def2-TZVP level of theory for TAM2 and TAM3 dyes. We extracted dye geometries from molecular dynamics trajectories (OPC3-FW 400 K simulations with both type A and B parametrization, see Section 2.2.2(i) for details) of the air–water interface and bulk water, selecting 50 evenly spaced frames from each trajectory. For each frame, we retained the 100 closest water molecules to the dye to define an explicit local environment. “Closest” waters were identified by comparing the distance between the water center of mass (COM) and the convex hull of the dye atoms; waters whose COM lay inside the hull were included automatically. This procedure yielded 400 snapshots total (50 bulk +50 interfacial geometries per dye per parametrization). For each snapshot, we computed the 5 lowest TDDFT transitions at ωB97X/def2-TZVP. Individual spectra were Lorentzian-broadened (fwhm = 0.20 eV), summed and averaged within each ensemble, and normalized such that the maximum absorbance equals 1.

#### 2.1.3 Ab Initio Charge Calculations

To prepare restrained electrostatic potential (RESP) charges for classical MD simulations, the geometries of TAM0–3 and TAM3I were optimized using DFT at the ωB97X-D4 ^47^/def2-TZVP^48^ level. RESP charges were then generated from single-point calculations on the optimized geometries in vacuum at the ωB97X-2 ^49^/def2-TZVP level. Atom-centered partial charges were obtained by fitting to the electrostatic potential, followed by RESP charge fitting as implemented in the Multiwfn software.^50^

#### 2.1.4 Dye Dimer Analysis

Dimer geometry observed in multidye slab MD simulations of TAM3 was extracted for electronic-structure analysis. Similar geometry was taken for TAM0–2 dimers by replacing corresponding −N­(CH_3_)_2_ groups in the TAM3 dimer structure. The dimers were then optimized using the ωB97X-2 double hybrid functional with def2-TZVP basis to account for dispersion interactions. Optimizations were performed with implicit solvation (SMD water).^51^ Interaction energies were estimated as E_int_ = E_dimer_ – 2E_monomer_ (negative values indicate stabilization).

#### 2.1.5 BOMD Simulations of TAM3 Microhydrated Cluster

For TAM3­(H_2_O)_80_ cluster additional BOMD simulation at GFN2-xTB level of theory was performed. The initial geometry was taken from the microhydrated-cluster structure search described in Section 2.1.1. The system was propagated in the NVT ensemble at 350 K using a 0.5 fs time step for a total of 20 ps. Temperature control was achieved with a Nose–Hoover chain thermostat with a 10 fs time constant.

### 2.2 Classical MD Simulations

All molecular dynamics simulations were performed using Amber 22 ^52^ and AmberTools 23.^53^ All Amber simulation systems were prepared with tleap in AmberTools. Periodic boundary conditions were applied in all simulations. Long-range electrostatic interactions were treated using the particle mesh Ewald (PME) method.^54,55^ The distance cutoff for all nonbonding interactions was set to 8 Å.

#### 2.2.1 Dye Parametrization

Dye parameters were generated with the Antechamber/parmchk2 workflow using the General AMBER Force Field 2 (GAFF2).^56^ Antechamber was used to assign GAFF2 atom types and bonded terms,^57^ while atomic partial charges were taken from the RESP procedure^58^ described in Section 2.1.3. We note that Antechamber commonly uses AM1-BCC charges^59^ by default; in this work, AM1-BCC was not used for production parametrization, and RESP charges were used throughout. During atom typing, we observed that default automated assignment labels one N-containing aryl ring in TAM1, TAM2, and TAM3 with a quinone-like pattern. Because this choice can affect torsional preferences and related observables, we used two parametrization types: type A with default Antechamber parametrization and type B with equivalent aromatic-rings parametrization and same central-carbon type across all the TAM series. In GAFF2 atom-type terms, in type A the central carbon is defined as “ce” and one amino-substituted aryl ring is typed with a quinone-like “cd”/“cc” pattern while the rest carbons in aryl rings are types as “ca”. In type B, the central carbon is defined as “c2” and all aryl-ring carbons are typed as “ca”. Both types were used in MD simulations.

#### 2.2.2 Water Parametrization and MD Simulation Setups

To assess the sensitivity of dye behavior to the solvent model and simulation conditions, we performed multiple MD simulations under several different setups, summarized below. To probe different diffusion and surface roughness regimes on accessible simulation time scales, we performed a set of simulations in which water intramolecular geometry was not constrained (flexible-water treatment), including for models commonly parametrized in rigid-water form. Throughout this manuscript, such runs are explicitly labeled with the suffix FW (e.g., TIP3P-FW, OPC3-FW, OPC3-pol-FW). To benchmark this approximation, we additionally compared intramolecular water-geometry distributions against a genuinely flexible reference model (SPC-FW). Specifically, the O–H bond-length and H–O–H angle distributions were analyzed and found to be comparable across all the flexible-water setups (SPC-FW, TIP3P-FW, OPC3-FW, OPC3-pol-FW) used here (see Figures S52, S53, Supporting Information).

##### 2.2.2.1 Single-Dye Bulk Water Systems (TIP3P­(−FW), OPC3­(−FW), OPC3-Pol­(−FW), SPC-FW; 300 and 400 K)

Bulk-water simulations were first performed to characterize dye behavior in fully solvated environments without interfacial effects (Table S1). Each dye (TAM0–3 and TAM3I) was solvated in a cubic box of explicit water (60 Å × 60 Å × 60 Å) using either nonpolarizable TIP3P,^60^ OPC3,^61^ SPC-FW^62^ or polarizable OPC3-pol^63^ water models. For OPC3, the water molecule counts were 5691 (TAM2, TAM3), 4459 (TAM0), 4811 (TAM1), and 5669 (TAM3I). For OPC3-pol, the corresponding water counts were 5693 (TAM3), 5695 (TAM2), 4395 (TAM0), 4772 (TAM1), and 5671 (TAM3I). For TIP3P, the water counts were 5655 (TAM3), 5664 (TAM2), 4295 (TAM0), 4757 (TAM1), and 5578 (TAM3I). For SPC-FW, the corresponding water counts were 4293 (TAM0), 4755 (TAM1), 5662 (TAM2), 5652 (TAM3), 5576 (TAM3I). Chloride counterions were added at a 1:1 stoichiometric ratio to neutralize the cationic dye. Each system underwent energy minimization, followed by heating to 300 K (or 400 K for accelerated sampling) over 100 ps. The systems were then equilibrated for 500 ps under NPT ensemble. Production runs were carried out for 30 ns, and coordinates were saved every 1 ps for analysis. Temperature was controlled using Langevin dynamics^64^ with a collision frequency of 2.0 ps^–1^, and pressure was maintained at 1 bar using an isotropic Berendsen barostat^65^ with a relaxation time of 1.0 ps. A time step of 0.5 fs was used throughout all simulation stages.

##### 2.2.2.2 Single-Dye Slab Systems (OPC3­(−FW), OPC3-Pol­(−FW), SPC-FW, TIP3P-FW; 300 and 400 K)

To investigate dye behavior at the air–water interface, single-dye slab systems were constructed in a rectangular periodic simulation box with dimensions 60 Å × 60 Å × 100 Å. Same number of water molecules as specified above was used. Each dye (TAM0–3, and TAM3I) was solvated in explicit water box (OPC3­(−FW), OPC3-pol­(−FW), TIP3P-FW, or SPC-FW), and vacuum gaps were introduced along the *z*-axis to create two equivalent air–water interfaces perpendicular to z. Chloride counterions were added at a 1:1 stoichiometric ratio to neutralize the cationic dye. For each system, simulations were initialized by energy minimization, followed by heating to 300 or 400 K over 100 ps. Equilibration was then performed for 500 ps under NVT using a 0.5 fs time step. Production trajectories were generated for 30 ns, with coordinates saved every 1 ps. Temperature was controlled using Langevin dynamics with a collision frequency of 2.0 ps^–1^.

To improve statistical sampling and assess reproducibility, we additionally performed replica simulations: for each system and temperature, we ran 100 independent trajectories following the same minimization/heating protocol and a short production segment. The complete protocol was repeated at both 300 K (OPC3-FW, OPC3-pol-FW, OPC3) and 400 K (OPC3-FW).

##### 2.2.2.3 Multidye Slab Systems (OPC3-FW; 400 K)

Multidye slab systems were constructed following the same protocol as the single-dye slab simulations, but with ten identical dye cations per system. Separate systems were prepared for TAM0–3. Initial 10-molecule configurations were generated using Packmol^66^ with random placement and a minimum intermolecular separation of 20 Å to minimize steric overlaps. The simulation box dimensions were 120 Å × 120 Å × 300 Å, providing sufficient lateral area and vacuum separation along the z direction to maintain two stable interfaces.

Each of the five multidye slab systems was simulated independently. Simulations began with energy minimization, followed by heating to 400 K over 100 ps. Temperature was controlled using Langevin dynamics with a collision frequency of 2.0 ps^–1^. The systems were then equilibrated for 500 ps under NVT using a 0.5 fs time step. Production trajectories were generated for 30 ns, with coordinates saved every 1 ps for analysis.

### 2.3 Analysis of MD Trajectories

Trajectory analysis was performed using custom Python scripts to quantify dye position, orientation, water structure, diffusion, and chloride ion behavior at the air–water interface.

#### 2.3.1 Density Profiles

For each trajectory frame, the COM of all water molecules and the dye molecule was calculated. To remove overall system drift, we recentered each frame by subtracting the COM of the full system from all component COM coordinates. The simulation box was then divided into uniform bins along the *z*-axis with a thickness of 0.25 Å, and the number of water molecules and dye molecules that fell within each bin was counted. These bin counts were averaged over all analyzed frames to obtain time-averaged number-density profiles. The air–water interface was defined as the z-position where the number-density decreases to 50% of the maximum number-density value.

#### 2.3.2 Molecular Orientation

The orientation of the dye molecule at the air–water interface was analyzed by tracking the orientation of its best-fit molecular plane relative to the simulation *z*-axis. For each trajectory frame after interfacial adsorption (i.e., once the dye COM entered and remained within the interfacial region defined from the water density profile), we used only dye non-hydrogen atoms to define a plane as follows: the atom Cartesian coordinates were first centered by subtracting their mean position, so that the molecular centroid coincided with the coordinate origin. The least-squares best-fit plane was then obtained by principal-component analysis (equivalently, SVD) of the centered coordinates. The plane normal was taken as the eigenvector corresponding to the smallest eigenvalue (the direction of minimal variance), and was normalized to unit length.

The orientation angle θ was then defined as the angle between this plane-normal vector **n** and the unit vector along the *z*-axis (***ẑ***) (Figure 2):

1 $$\theta =\cos^{-1}\left(\left|n\cdot ẑ\right|\right)$$

where the absolute value accounts for the **n** and −**n** degeneracy, restricting θ to 0–90°. The angle θ was recorded for each analyzed frame and compiled into histograms to obtain orientational distributions that report the preferred alignment of the dye plane at the interface over time.

**Figure 2:**
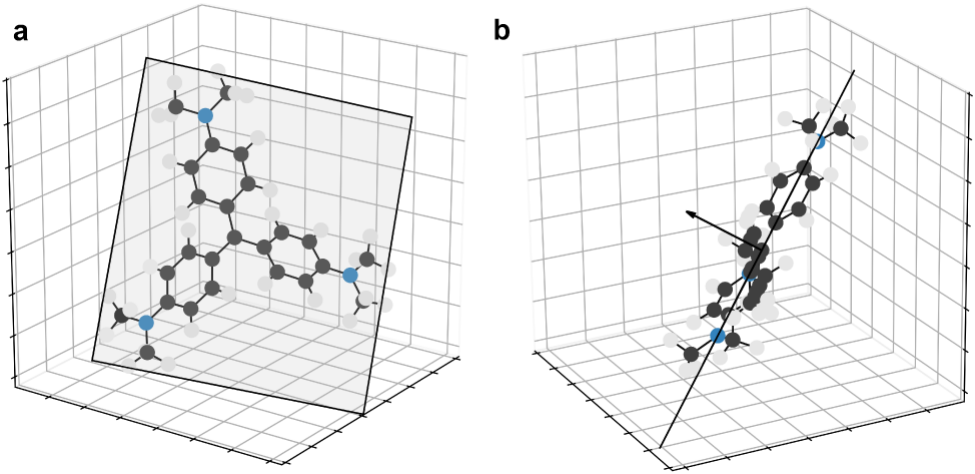
Best-fit plane to the TAM3 dye molecule shown from (a) a top view and (b) a side view. In panel b, the arrow indicates the unit normal vector to the fitted plane, used to define the molecular orientation relative to the interface.

#### 2.3.3 Water Diffusion Analysis

Water diffusion coefficients were obtained by linear least-squares fitting of the mean-square displacement (MSD) curves over the diffusive time window computed from water-molecule COM trajectories. To account for interfacial anisotropy, we evaluated diffusion separately parallel and perpendicular to the interface. The in-plane MSD was defined as

2 $$MSD_{xy}\left(t\right)={\langle [x\left(t\right)-x\left(0\right)]}^{2}+{[y\left(t\right)-y\left(0\right)]}^{2}\rangle$$

and the out-of-plane MSD as

3 $$MSD_{z}\left(t\right)={\langle [z\left(t\right)-z\left(0\right)]}^{2}\rangle$$

Diffusion coefficients were extracted from the line slopes using the Einstein relations:

4 $$D_{xy}=\frac{1}{4}\frac{dMSD_{xy}\left(t\right)}{dt},\;D_{z}=\frac{1}{2}\frac{dMSD_{z}\left(t\right)}{dt}$$

#### 2.3.4 Counterion Distribution

Counterion behavior was characterized by tracking the chloride ion position relative to both the dye and the air–water interface throughout the trajectory. For each analyzed frame, we recorded the drift-corrected chloride z-coordinate to obtain its spatial distribution across the slab. In addition, we computed the chloride-dye distance as the distance between the chloride ion and the dye center of mass. Chloride ion z-position trajectories, histogram distributions, and chloride-dye distances were then used to assess counterion localization with respect to the interface and the extent and dynamics of ion-dye association over time.

#### 2.3.5 Water Interfacial Roughness

Two complementary approaches were used to quantify the roughness of the air–water interface. The first approach is based on fitting the interfacial water number-density profile ρ­(z) with a sigmoid function,^67^ treating the top and bottom sides of the slab separately:

5 $$\rho_{top}\left(z\right)=\frac{\rho_{l}}{2}\left[1-\tanh\left(\frac{z-z_{G}^{top}}{\delta_{top}}\right)\right]$$

6 $$\rho_{bot}\left(z\right)=\frac{\rho_{l}}{2}\left[1+\tanh\left(\frac{z-z_{G}^{bot}}{\delta_{bot}}\right)\right]$$

where *ρ_l_* is the bulk liquid number density, *z_G_* is the Gibbs dividing surface position, and *δ* is an effective interfacial width that reflects the magnitude of the roughness caused by capillary waves.

The second approach is a more local measure of instantaneous fluctuations. In this approach we represented each interface by a pixelized height field z­(x,y) defined on a uniform xy grid. The simulation cross section was divided into square pixels of side length Δl (1.0 Å). For each frame, water atoms were mapped into this grid with periodic boundary conditions in x and y. The local surface height in each pixel was defined as the maximal (top) or minimal (bottom) z reached by the surface of the union of van der Waals spheres representing the water atoms (with radii r_O_ = 1.52 Å and r_H_ = 1.20 Å). Interfacial roughness was reported as the RMS fluctuation of this height field about its mean:

7 $$R_{q}=\sqrt{{{\langle (z\left(x,y\right)-{\left\langle z\right\rangle }_{x,y})}^{2}\rangle }_{x,y}}$$

where ⟨·⟩_*x*,_ *_y_* denotes averaging over pixels with defined surface height, and top and bottom interfaces were analyzed separately.

## 3 Results and Discussion

### 3.1 Microhydration

We began by characterizing microhydrated dye cations using low-energy cluster structures obtained from the DOCKER search. We explored the stoichiometries from TAM­(H_2_O) to TAM­(H_2_O)_80_ gradually increasing the number of water molecules. As expected, introduction of amine substituents increases the polarity of the chromophore and creates electrostatically favorable hydration sites. Thus, for the N-containing dyes (TAM1-TAM3), the first several water molecules preferentially coordinate to the amine nitrogens (Figure 3b–d). With increasing hydration number, additional waters aggregate into a nanodroplet in which the dye’s more hydrophobic aryl framework tends to remain partially not covered with water molecules.

**Figure 3:**
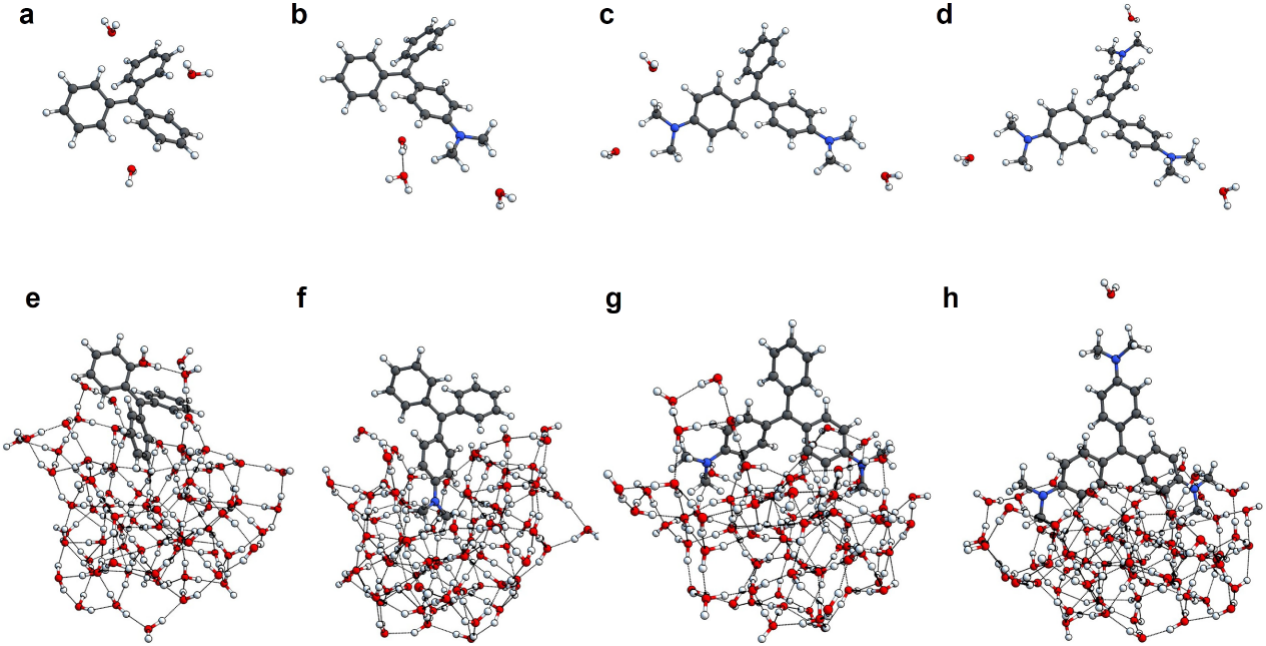
Representative low-energy microhydrated structures of the TAM dyes obtained from the DOCKER search. Panels a–d show TAM­(H_2_O)_3_ clusters for TAM0, TAM1, TAM2, and TAM3, respectively. Panels e–h show the corresponding TAM­(H_2_O)_80_ microhydrated clusters.

In the optimized 0 K microhydrated minima, the dyes typically adopt a configuration in which the chromophore is oriented approximately “vertically” relative to the growing water droplet (Figure 3f–h). TAM0 is an exception, which more readily adopts a “horizontal” orientation (Figure 3e). As shown below, this “vertical” preferred orientation is not retained once thermal fluctuations are included and the environment approaches a condensed-phase regime.

To probe temperature effects directly, we performed a short BOMD simulation for TAM3­(H_2_O)_80_ at the GFN2-xTB level starting from an optimized microhydrated 0 K minimum. Over 20 ps at 350 K, the dye is expelled from the interior of the microdroplet and relaxes to a surface-bound configuration in which the chromophore lies substantially flatter on the droplet exterior (Figure S51). This qualitative change indicates that the “vertical” orientation observed in 0 K cluster minima is primarily an enthalpic feature of optimized structures and can be destabilized by entropic contributions and thermal fluctuations. The resulting flatter configuration is consistent with larger contact area between the dye’s aromatic framework and the water cluster surface, increasing stabilization coming from dispersion interactions.

### 3.2 Classical MD Simulations of Bulk and Interfacial Solvation

We next analyzed dye behavior in explicit-solvent MD simulations in both bulk-water and slab (air–water interface) setups. Across the full simulation set (including multiple water models and temperatures), all TAM dyes exhibited clear interfacial accumulation once they reached the slab surface. To improve sampling of diffusion-limited approach to the interface within accessible trajectory lengths, we focus here on the OPC3-FW simulations at 400 K, which provide substantially faster water and dye diffusion while preserving the qualitative interfacial trends discussed below.

Figure 4 shows the z-resolved probability density profiles of the dye COM for the TAM series, averaged over the one hundred 10 ns trajectories and over all analyzed time frames. For reference, dashed vertical lines mark the formal interfacial positions determined from the water density profile (defined at ρ­(z)/ρ_max_ = 0.5; Section 2.3.1). The profiles exhibit pronounced maxima near the two air–water interfaces, confirming strong interfacial enrichment for all dyes.

**Figure 4:**
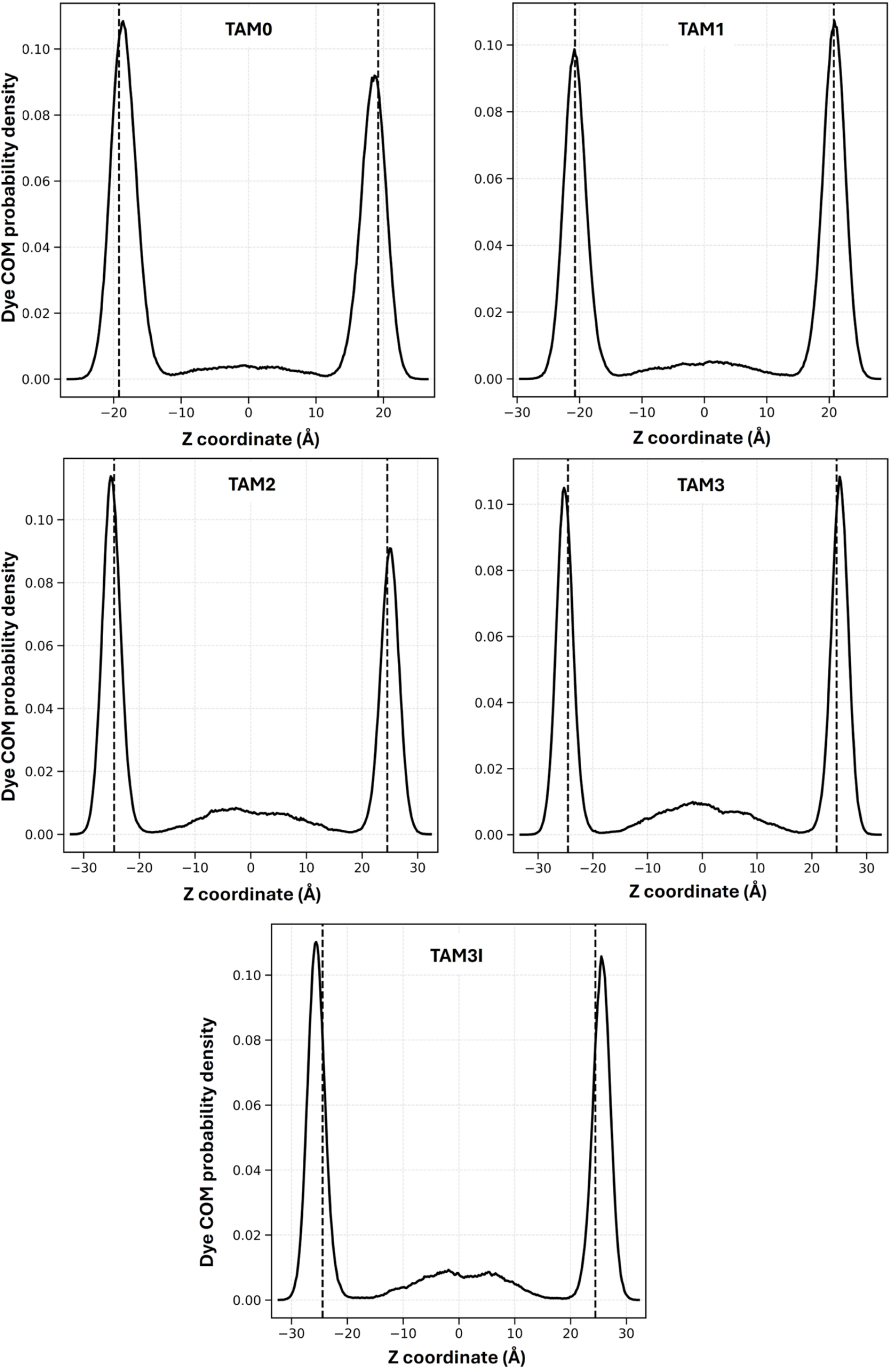
Normalized probability density profiles of the dye COM z-coordinate for TAM0–3 and TAM3I type B parametrization obtained from OPC3-FW slab simulations at 400 K, averaged over 100 independent trajectories and all frames. Vertical dashed lines indicate the formal air–water interfacial positions extracted from the water number-density profile using the ρ­(z)/ρ_max_ = 0.5 criterion (Section 2.3.1).

At the same time, the position and width of the interfacial maxima depend systematically on dye structure. TAM0 shows maxima located slightly deeper in the water phase (i.e., below the formal interface), indicating a more submerged average COM position. As aryl substitution increases across the series, the interfacial maxima shift toward the interface boundary. This trend is most pronounced for TAM3I, whose COM density maximum lies partially beyond the formal interface position, consistent with more hydrophobic framework.

In addition to the sharp interfacial peaks, all dyes exhibit a smaller but nonzero density in the water slab. It reflects the finite time required for diffusion from the bulk-like region to the interface.

To quantify how rapidly TAM dyes reach the interface, we tracked the time dependence of the dye COM coordinate relative to the water slab (|*z_COM_*,*_dye_* – *z_COM_*,*_water_* |) across the ensemble of independent trajectories. Representative ensemble-averaged displacements for TAM0–3 and TAM3I are shown in Figure 5. In all cases, the mean displacement increases monotonically toward a plateau corresponding to the interface location, consistent with a diffusion to the interface followed by interfacial “trapping”.

**Figure 5:**
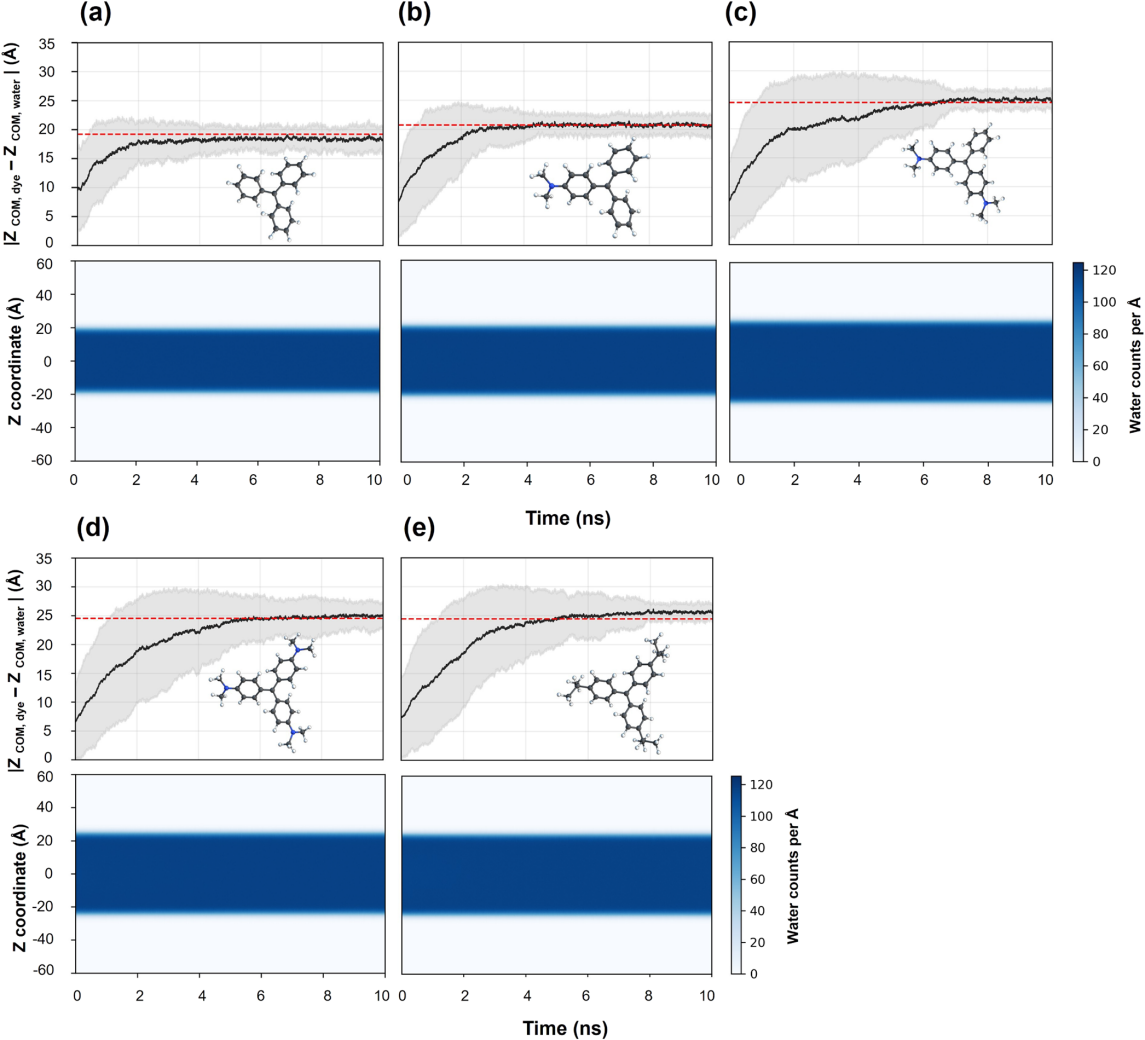
Ensemble-averaged COM_water_-corrected z-coordinates of COM_dye_ for (a) TAM0, (b) TAM1, (c) TAM2, (d) TAM3, and (e) TAM3I dyes type B parametrization (top panels). Solid black lines show the mean over independent trajectories; shaded regions indicate the standard deviation across trajectories. The horizontal red dashed line marks the formal air–water interfacial positions. Bottom panels show corresponding time-resolved water counts per Å maps.

A key qualitative observation is that once the dye reaches the interfacial region it almost never returns to the bulk on the time scale of our simulations. Across the full trajectory set, only a few clear events when dye solves back into the bulk water was observed (TAM0 in OPC3-pol, OPC3 at 300 K, TIP3P-FW at 400 K, Figures S33, S35, and S36). This behavior is consistent with strong interfacial affinity and suggests that the dominant long-time kinetics are controlled by the first-passage process of reaching the interface, rather than repeated adsorption–desorption equilibria within the sampled time window.

The shaded gray bands in Figure 5 report the standard deviation of COM_dye_ displacement. The apparent decrease of the bandwidth at long times reflects convergence as an increasing fraction of trajectories have reached the interfacial plateau. To quantify the rate of interfacial deposition, we fit the ensemble-averaged curves to a single-exponential relaxation,

8 $$\left\langle z\left(t\right)\right\rangle =z_{\infty }-\left(z_{\infty }-z_{0}\right)e^{-t/\tau }$$

where *z*_∞_ is the long-time plateau and τ is an effective time constant summarizing the rate of dye deposition on the interface under a given simulation setup (Table 1). The resulting τ values are on the order of ∼1–2 ns and increase systematically across the series, consistent with slower diffusion for larger dyes.

**Table 1:** Exponential-Fit Parameters for Ensemble-Averaged Z-Coordinates of COM for TAM0-3 and TAM3I Dyes and Their Effective Collision Diameters σ

Dye/Parametrization	*z*_∞_ [Å]	τ [ns]	σ [Å]
TAM0/B	18.4	0.84	8.59
TAM1/B	20.8	1.11	9.23
TAM1/A	20.8	0.97	9.23
TAM2/B	25.0	1.95	10.10
TAM2/A	24.9	1.77	10.10
TAM3/B	25.2	1.97	10.93
TAM3/A	25.8	2.12	10.93
TAM3I/B	25.8	1.91	11.17

Importantly, the fitted time constants show only modest sensitivity to the atom parametrization (type A vs B, Figure S48 and Table 1) for TAM1-TAM3, indicating that the approach kinetics are primarily controlled by size and diffusion, whereas the parametrization choice more strongly impacts intramolecular dihedral angle ensembles (discussed below). Within the present data set, the polarity differences among substituents do not produce large changes in the time constants.

We next examined the coupled distribution of dye orientation and position relative to the water slab by correlating the dye-plane angle θ with the dye COM displacement (Figure 6). Here θ is defined as the angle between the dye plane normal and the *z*-axis. Thus, small θ corresponds to a flatter or “horizontal” interfacial configuration (molecular plane approximately parallel to the interface), whereas large θ corresponds to an upright or “vertical” configuration.

**Figure 6:**
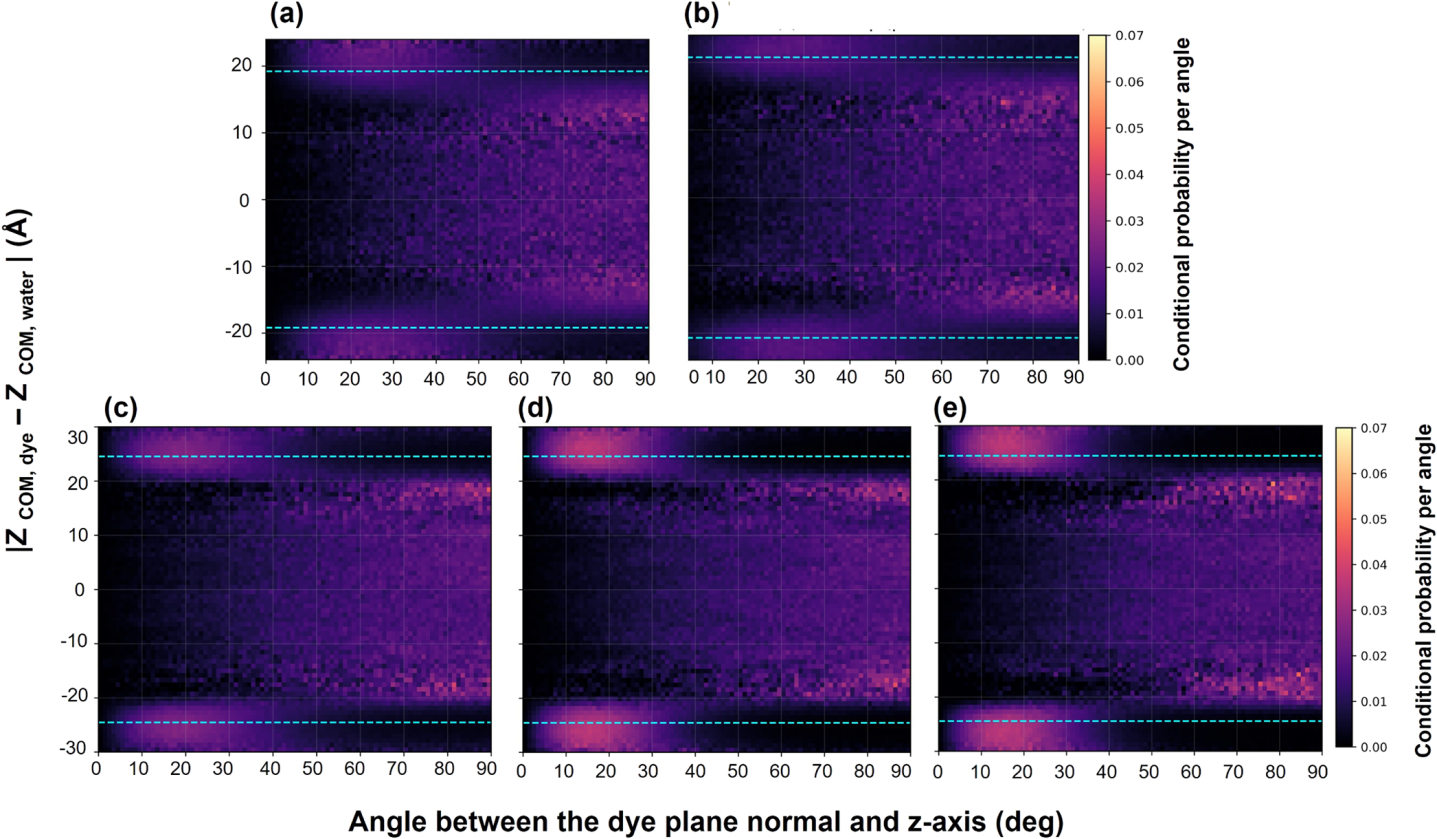
Two-dimensional conditional probability per angle maps correlating dye orientation and position relative to the water slab for (a) TAM0, (b) TAM1, (c) TAM2, (d) TAM3, and (e) TAM3I dyes (type B parametrization) in OPC3-FW slab simulations at 400 K. The horizontal axis is the angle θ between the dye-plane normal and the *z*-axis (small θ: “horizontal” molecule orientation; large θ: “vertical” molecule orientation). The vertical axis is the COM_dye_ displacement. Dashed horizontal lines mark the formal interfacial positions.

For all TAM dyes, the highest-probability interfacial region (region near the interface lines) is dominated by small θ, indicating that the dyes preferentially adopt a flat orientation once reach the interface. The sharpness of this orientational preference increases with dye size. Smaller dyes (e.g., TAM0) display broader angular distributions, while larger dyes (TAM3 and TAM3I) show a more pronounced bias toward certain small angle θ (see discussion below), consistent with stronger dispersion-driven stabilization of interfacial contact for larger organic molecules. In bulk water, the dye shows no preferred orientation; accordingly, the angle distribution follows the expected sin­(θ) form for isotropically oriented molecules (Figures S8, S10, S12, S14, and S16).

In addition to the adsorbed state, Figure 6 reveals a distinct, more upright population at intermediate |z| during the approach to the interface. Specifically, a higher-probability band at large angles (θ ∼ 60°–90°) emerges slightly below the formal interface line, indicating that dyes often approach the interface in a vertical configuration and then reorient to a flatter geometry upon adsorption. This observation supports a two-step adsorption pathway: (1) diffusion toward the interfacial region with preferential upright/tilted configurations when the molecule is close to the interface boundary, followed by (2) rotational relaxation into a flat, surface-bound state.

The physical origin of this behavior is the progressive asymmetric reorganization of the dye solvation shell as the molecule enters the interfacial region. For the clarity of further discussion, we will approximate the dye as a rigid disk. At a fixed dye COM position, the set of all disk orientations spans a sphere. Because the slab of water is isotropic in x and y directions, rotations about these axes are equivalent, so it is sufficient to consider rotations of the disk about a single axis (here we will pick *y*-axis). In the xz projection (see top panel of Figure 7 for clarity), each disk configuration is represented by a line of fixed length.

**Figure 7:**
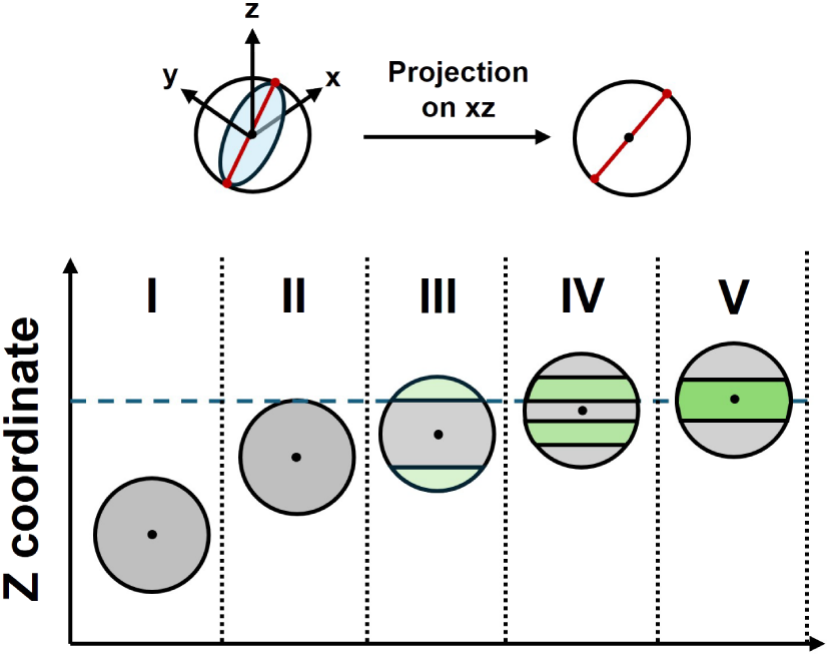
Schematic illustration of an orientation dependent stabilization mechanism near the interface using a disk model. Top: example of projection of a disk rotated around *y*-axis on xz plane. Circle represents all possible disk orientations. Bottom: qualitative stages of interfacial solvation (I–V) as a function of the disk COM z-coordinate. Green shading denotes energetically more favorable disk orientations; a greener color indicates a more stable orientation.

Within this model, we can consider distinct stages of molecule approaching the interface (Figure 7). Stages I and II formally correspond to bulk solvation, where the dye remains surrounded by essentially intact hydration shell, and no strong orientational preference is expected. As the dye approaches the interface (stage III), configurations start appearing where the upper part of the hydration shell partially reorganizes. Water molecules that would otherwise solvate the dye are released and become a part of the hydrogen bond network of the liquid, which is energetically more favorable compared to maintaining dye-water contacts. This process stabilizes “vertical” orientations, resulting in an orientation bias. As the COM moves closer to the interface (stage IV), very vertical orientations are no longer optimal because tilting increases favorable dispersion-driven contact of the dye with the interface. Finally, when the COM is at the interface (stage V), flatter configurations maximize interfacial contact area and are therefore the most stabilized. We note that energetic favorability of preferred orientations increases as we go from stage II to stage V. Consistent with this picture, this progressive stabilization produces an energy gradient that pushes dye toward interface, resulting in a lower density (concentration) region immediately beneath the formal interface boundary (Figure 4).

We next quantified the orientational distributions after interfacial adsorption by creating histograms of the plane-angle θ (Figure 8). All TAM dyes show a clear orientational bias toward small θ, confirming that the adsorbed state is dominated by flat configurations (molecular plane approximately parallel to the interface). The strength of this bias depends systematically on dye size: smaller dyes exhibit substantially broader distributions, whereas larger dyes (TAM3 and TAM3I) show a sharper peak at low angles, consistent with stronger stabilization of extended interfacial contact for larger dye molecules.

**Figure 8:**
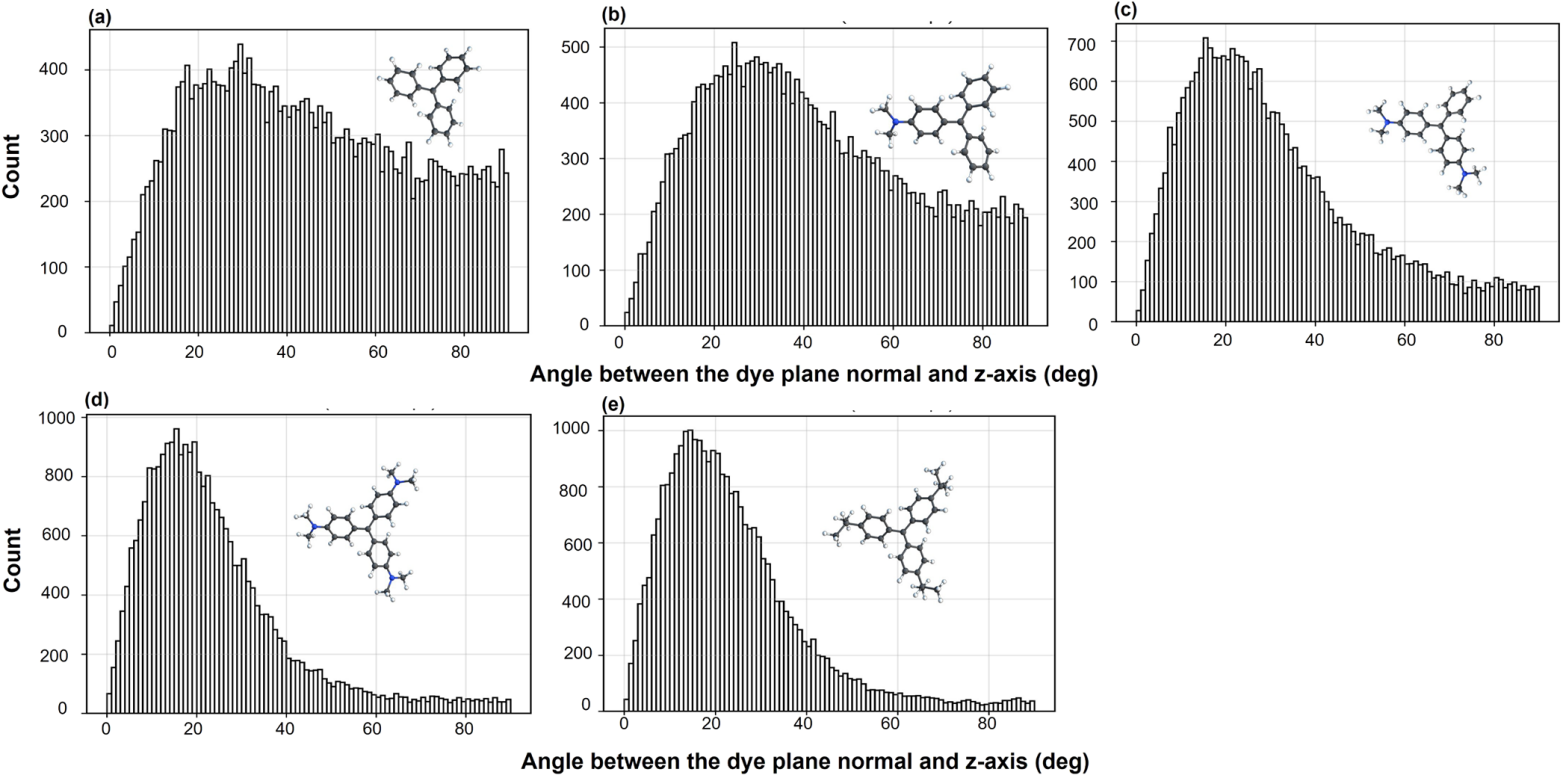
Orientational distributions of the TAM dyes (a) TAM0, (b) TAM1, (c) TAM2, (d) TAM3, and (e) TAM3I from single dye slab MD simulations at 400 K using the OPC3-FW water model. Only trajectory frames after interfacial adsorption were included in the analysis.

Across the simulation set, this qualitative trend is robust with respect to water model, temperature, and dye parametrization type. Temperature primarily modulates diffusion and interfacial roughness, which can broaden the low-angle peak and accelerate convergence of the interfacial ensemble, but it does not change the ordering across the TAM series.

In addition to the dominant low-angle population, all histograms exhibit a high-angle tail extending toward high θ values. Comparison with the joint (|z|,θ) distributions (Figure 5) indicates that this tail is associated with configurations in which the dye samples more subsurface or bulk-like positions, where upright/tilted orientations are more accessible. The tail is therefore most pronounced for smaller dyes, which more readily explore configurations deeper in the aqueous phase, and it becomes less prominent for larger dyes that remain more strictly surface-bound.

The counterion z-coordinate probability distributions show that chloride ions remain confined to the aqueous phase with peak populations centered within the bulk water region, confirming strong ion hydration (Figures S42 and S43). The distance distributions between the chloride ion and the dye COM exhibit is broad with a maximum around 20–35 Å, indicating that chloride ions remain predominantly solvent separated from the dye and no persistent contact ion pairing under the present conditions.

To probe concentration effects beyond the single dye limit, we additionally performed multidye slab simulations containing 10 dye cations per unit cell. In these runs, the dyes again accumulate at the air–water interface, consistent with the single dye results. In contrast to the dilute regime, we frequently observe persistent dye–dye aggregates, including dimers, trimers, and tetramers forming in the bulk for both parametrization types (Figure S54). For most trajectories, once formed, these aggregates do not dissociate over the full simulation window (30 ns), indicating slow dissociation kinetics. Though for some trajectories we observe a more dynamical behavior (growth and dynamical exchange of TAM dyes) of aggregates after their formation.

But how energetically plausible are these aggregates? To answer this, we extracted representative dimer geometries from the trajectories and carried out electronic-structure calculations. Dimer structures were optimized at the ωB97X-2/def2-TZVP level with implicit solvation (SMD, water). The resulting interaction energies indicate substantial stabilization upon dimer formation (TAM0: E_int_ = −13.6 kcal/mol; TAM1: E_int_ = −17.9 kcal/mol; TAM2: E_int_ = −21.4 kcal/mol; TAM3: E_int_ = −24.3 kcal/mol). The magnitude of stabilization increases with dye size across the TAM series, consistent with stronger attractive dispersion interactions for larger organic molecules. These results suggest that, despite electrostatic repulsion between cationic dyes, short-range dispersion interactions can dominate and yield stable aggregates.

### 3.3 Water-Model Dependence: Diffusion and Interfacial Roughness

It is important to assess how sensitive the dynamics and interfacial structure are to the water model used. In addition to standard rigid-water models (TIP3P, OPC3, OPC3-pol), we performed a set of simulations in which the intramolecular geometry of these models was not constrained (denoted “FW”). This choice introduces vibrational degrees of freedom to water molecules and can alter effective global properties like boiling temperature, viscosity and surface roughness, thereby changing the rate at which dyes reach the interface and potentially change orientation behavior of dye on the interface.

To quantify the interfacial structure, we characterized surface roughness using two complementary metrics. First, we fit the one-dimensional water number-density profile ρ­(z) to a sigmoid form (eqs 5 and eq 6) and extracted the interfacial width parameter δ, which provides a global measure of the density transition region. Second, we computed a roughness metric R_q_ from local surface height maps (Figure 9), defined as the root-mean-square fluctuation of the instantaneous interfacial height about its mean.

**Figure 9:**
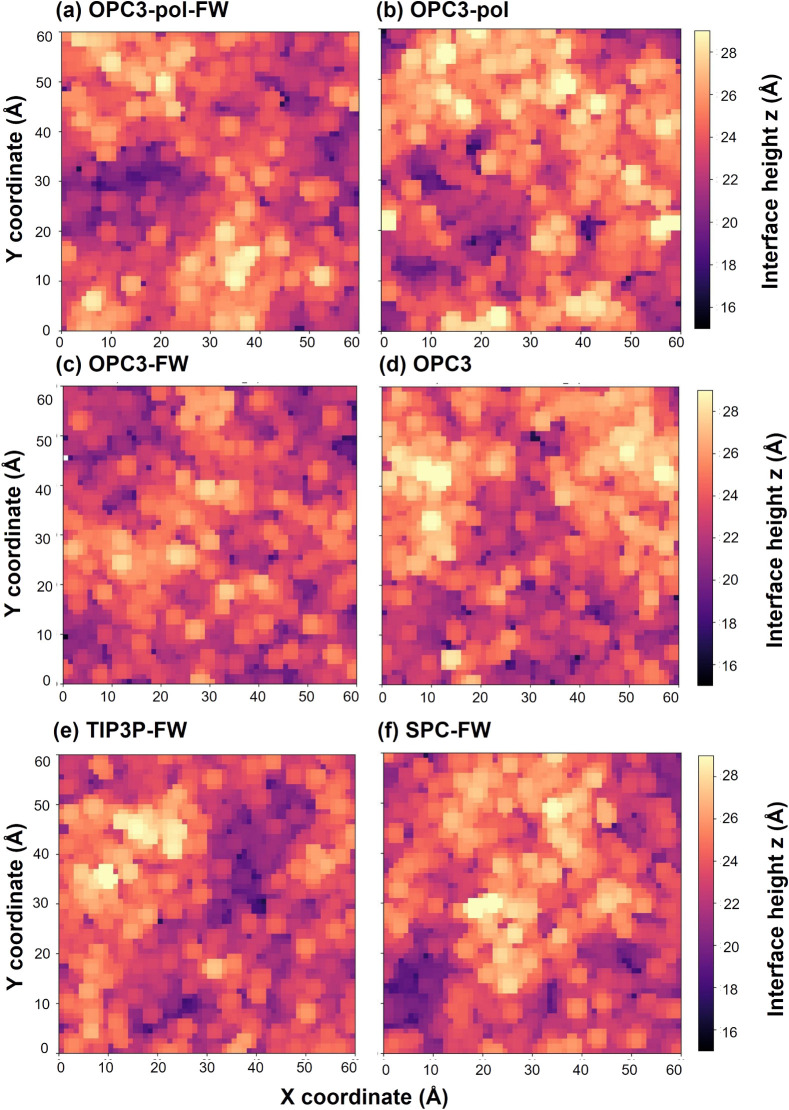
Surface height maps for random snapshots of MD simulation for different water models. Heat maps show the local interfacial height z­(x,y) (top surface) computed from the instantaneous outermost extent of nearby water molecules (see Section 2.3.5).

As summarized in Table 2, both the interfacial structure and water mobility depend strongly on the chosen model and temperature. Increasing the temperature from 300 to 400 K in OPC3-FW leads to a marked increase in water diffusion (D = 0.065→0.447 Å^2^/ps) accompanied by a broader and rougher interfacial region (δ = 1.58→2.30 Å; R_q_ = 1.78→2.32 Å). In contrast, allowing intramolecular flexibility in water models that are commonly used in rigid form substantially reduces translational mobility at 300 K: for OPC3, diffusion coefficient decreases from 0.191 Å^2^/ps (rigid) to 0.0654 Å^2^/ps (FW), while for OPC3-pol it decreases from 0.157 Å^2^/ps (rigid) to 0.100 Å^2^/ps (FW). The corresponding roughness metrics in the OPC3 family are smaller in the FW models at 300 K (δ ≈ 1.6 Å, R_q_ ≈ 1.8 Å) compared to the rigid counterparts (δ ≈ 1.8 Å; R_q_ ≈ 2.0 Å). Overall, these results show that both diffusion-controlled kinetics and subtle differences in interfacial roughness can vary appreciably across model choices, motivating the cross-model comparisons of dye adsorption and orientational statistics.

**Table 2:** Interfacial Width, Roughness, and Water Mobility for the Water Models Used in This Work

Model/Temp.	δ [Å]	R_q_[Å]	D [Å^2^/ps]
OPC3-pol-FW/300 K	1.64	1.79 ± 0.12	0.100
OPC3-pol/300 K	1.83	1.97 ± 0.16	0.157
OPC3-FW/300 K	1.58	1.78 ± 0.14	0.065
OPC3-FW/400 K	2.30	2.32 ± 0.17	0.447
OPC3/300K	1.80	1.96 ± 0.16	0.191
TIP3P-FW/300 K	1.96	2.01 ± 0.18	0.270
SPC-FW/300 K	1.87	1.97 ± 0.15	0.189

These water-model trends help rationalize differences observed in dye orientational statistics. In particular, conditions associated with smoother and less mobile interfaces (lower temperature/higher effective viscosity) tend to suppress the high-angle tail in the dye-plane histograms, consistent with reduced access to deeper, bulk-like configurations on the sampled time scales. Conversely, rougher, more mobile interfaces (i.e., lower viscosity) facilitate broader positional and orientational sampling during adsorption, leading to more pronounced “vertical” populations. Thus, at 300 K, the higher roughness and lower viscosity of OPC3-pol yield a non-negligible high angle tail, while the lower roughness and higher viscosity of OPC3-pol-FW at the same temperature largely eliminate it (Figure 10).

**Figure 10:**
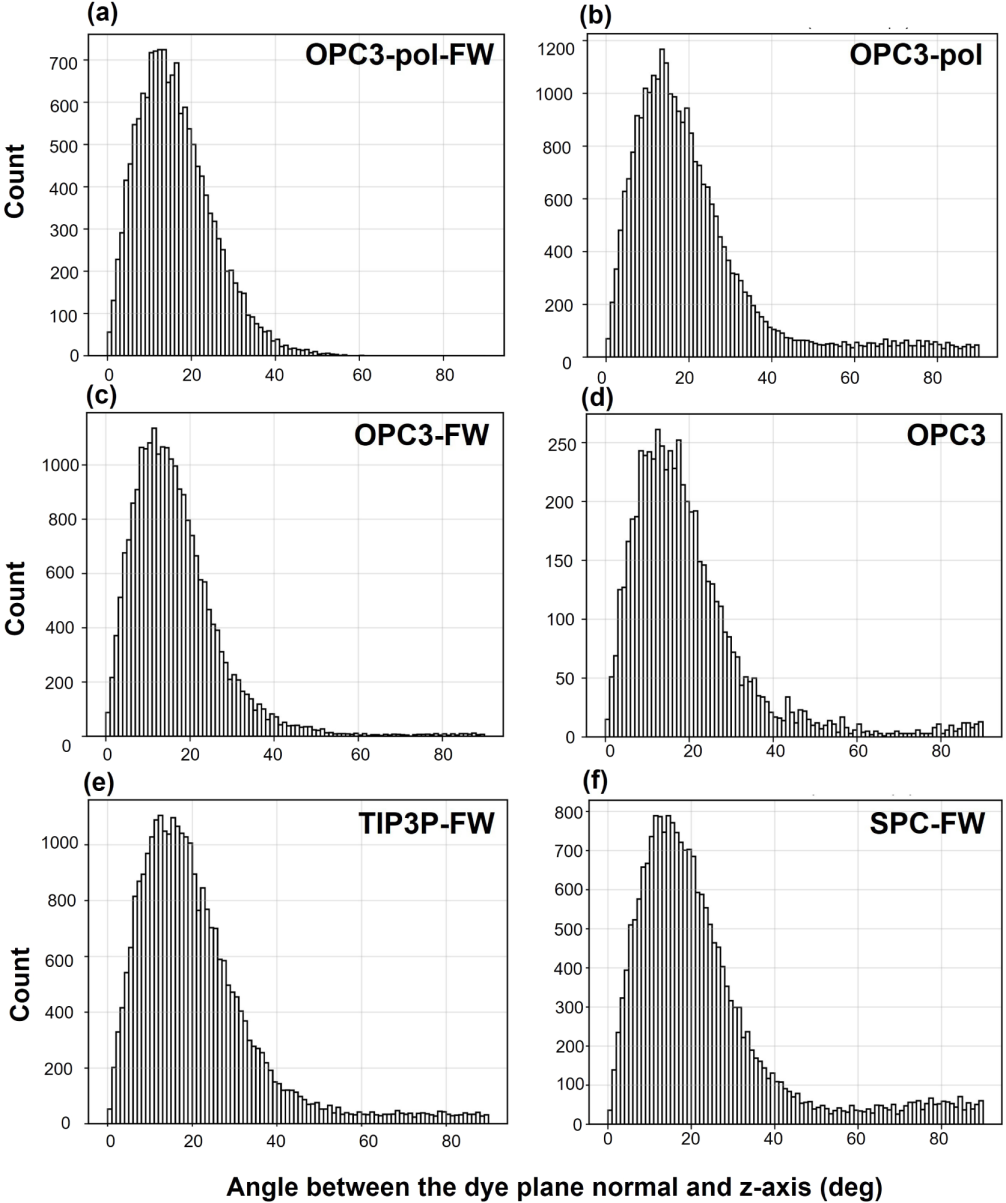
Orientational distributions of TAM3 obtained from single-dyed slab MD simulations at 300 K using different water models (a) OPC3-pol-FW, (b) OPC3-pol, (c) OPC3-FW, (d) OPC3, (e) TIP3P-FW, (f) SPC-FW.

### 3.4 Optical Spectra and Dihedral Angle Distributions

Optical spectra of triarylmethane dyes are expected to depend on the aryl torsions, as this degree of freedom effectively controls the conjugation between π-system. Since TAM dyes are conformationally flexible, UV–vis spectra would depend on ensemble distribution of dihedral angles corresponding to aryl rings rotation. We therefore first analyzed the distributions of the three corresponding dihedral angles (Figure S38) in the bulk and at the air–water interface (Figure 11, Figures S40, S41).

**Figure 11:**
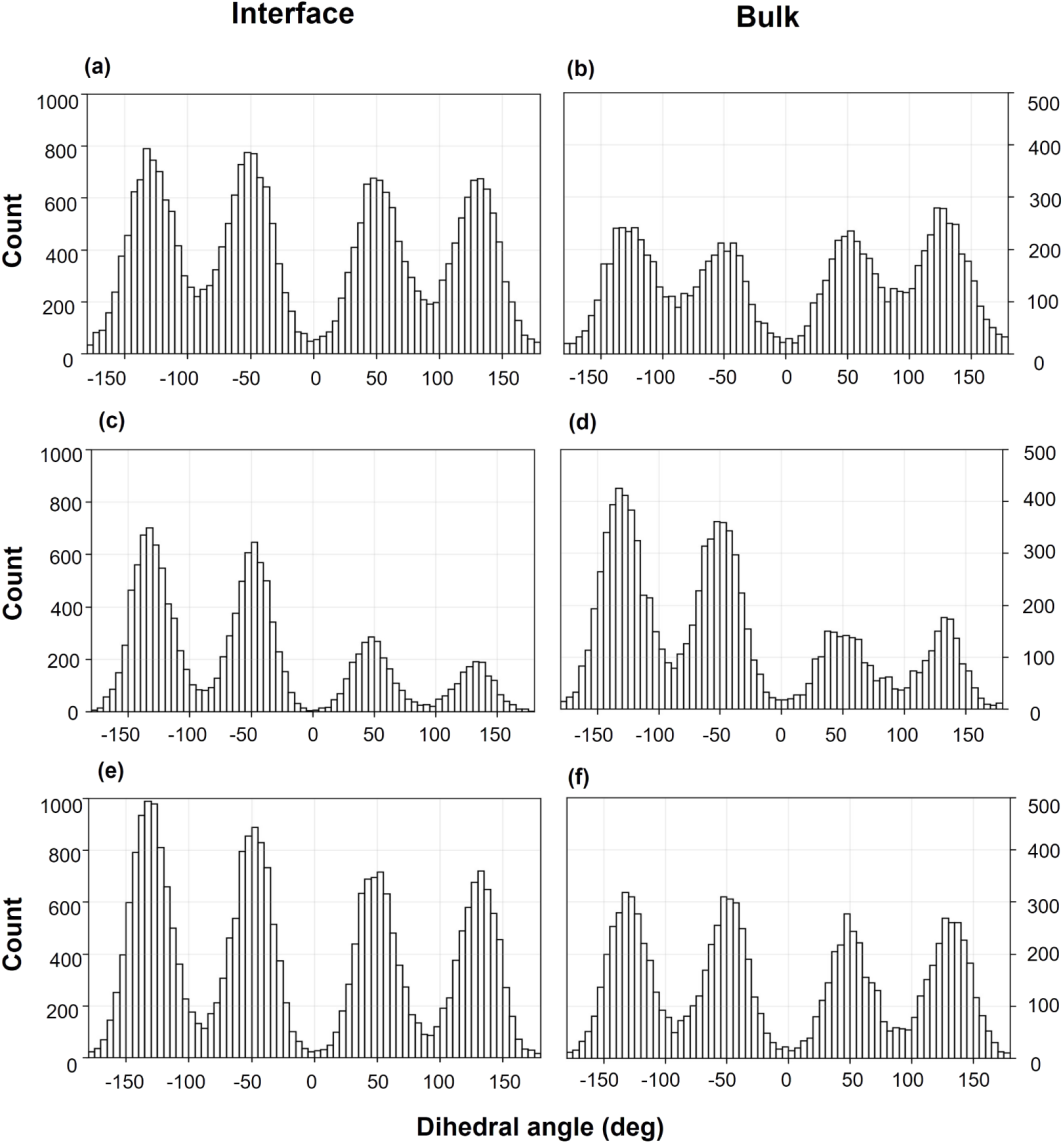
Dihedral angle distribution of the nonsubstituted aryl ring of TAM2 type B parametrization obtained from single-dyed slab and bulk MD simulations. (a, b) corresponds to simulations at 400 K with OPC3-FW, (c, d) 300 K with FW, and (e, f) 300 K with OPC3. Left panels (a, c, e) show slab simulations, and the right panels (b, d, f) show bulk water simulations.

Expectedly, the dihedral angle ensembles depend on dye parametrization type. In the type A, one ring is assigned a quinone-like structure, which biases its torsional profile (it has one local minima with mean value closer to 0° angle, Figure S41). The other two rings feature two peaks in the angle distribution and sample values from −150° to −15°. In contrast, symmetric type B assigns equivalent aromatic types to all three rings and yields to a similar dihedral angle distribution with four peaks. Different count distribution for different peaks is associated with the different energy barriers to change local minima configuration with certain dihedral angle. Thus, at lower temperatures and higher viscous solutions the population of right two peaks is smaller (Figure 11c,d) as we initially start with the conformation that correspond to −44° angle. At higher temperatures and/or lower viscosity, states separated with high energy barrier from the initial orientation become more evenly sampled (Figure 11a,b).

Independent of parametrization type, across the TAM series, the molecules at the air–water interface exhibit narrower (more peaked) dihedral angle distributions than the corresponding molecules in the bulk (Figure 11, S41, S40). Thus, intermediate torsional states between the preferred local minima are populated more for TAM solvated in the bulk. This difference in conformational distributions is a consequence of TAM interfacial binding. Once at the interface, the dye maximizes interfacial contact and experiences an anisotropic solvation environment that penalizes large torsional moves of aryls.

To connect conformational ensembles to spectroscopy, we computed TDDFT spectra for bulk and interfacial solvation using explicit water environments (100 nearest water molecules, Figure S55) extracted from MD simulations (Section 2.1.2). Figure 12 compares the ensemble-averaged absorption profiles for representative dyes. It is clear that two different parametrizations give different spectra. Interestingly, under the type B parametrization the bulk and interfacial spectra are broadly similar, and no systematic spectral shift was observed. Thus, the non-negligible spectral shifts observed only in quinone-like parametrization (type A).

**Figure 12:**
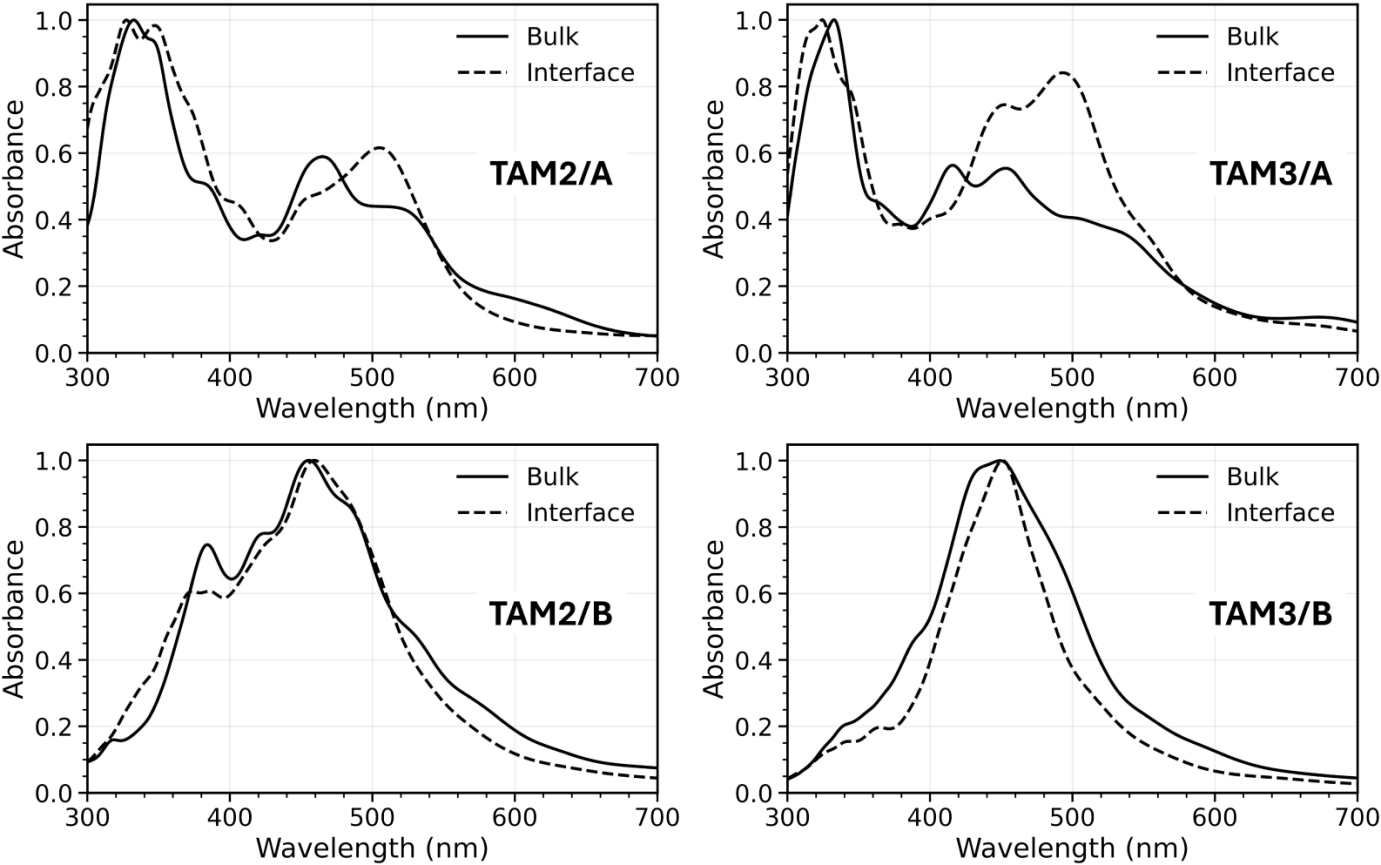
Ensemble-averaged TDDFT absorption spectra of TAM2 and TAM3 from MD-derived bulk and interfacial environments. Solid lines show bulk spectra and dashed lines show interfacial spectra, computed by averaging TDDFT results over 50 MD snapshots with explicit local solvation (100 nearest water molecules). Top row: type A parametrization; bottom row: type B parametrization.

## 4 Conclusions

In this work, we combined microhydration analysis, classical molecular dynamics, and TDDFT calculations to clarify how a structurally controlled series of triarylmethane dyes (TAM0-TAM3I) behaves in bulk water and at the air–water interface. Low-energy 0 K microhydrated minima capture the expected trend with water molecules initially bind at amine nitrogens in TAM1-TAM3, but they do not reliably predict finite-temperature orientation. In particular, a short BOMD trajectory for TAM3­(H_2_O)_80_ shows that thermal fluctuations drive the dye toward a surface-bound state where it substantially lies flatter.

Across slab MD simulations, all TAM dyes display strong interfacial affinity. In the OPC3-FW/400 K ensemble used for efficient sampling, for a 40–50 Å water slab, adsorption proceeds on nanosecond time scales and the ensemble-averaged COM_dye_ plots are well fitted by a single-exponential relaxation with τ ∼ 1–2 ns that increases with dye size. Once a dye reaches the interfacial region it almost never returns to the bulk within the simulated windows, indicating that repeated adsorption–desorption events are rare. The preferred interfacial COM_dye_ position shifts systematically across the series: TAM0 resides slightly deeper in the aqueous phase, whereas progressively substituted dyes move closer to (and for TAM3I partially beyond) the formal interface boundary. Joint angle-position maps further reveal a common adsorption pathway in which dyes often enter the interfacial region in “vertical” orientation and then relax into a “horizontal”, surface-bound orientation. After adsorption, all dyes show a strong bias toward flat configurations, with the orientational distributions narrowing notably as dye size increases.

Multidye slab simulations reveal persistent aggregation in the bulk, with dimers, trimers, and tetramers forming and mostly remaining intact over 30 ns. Electronic-structure calculations of TAM dimers with implicit solvation showed a significant interaction energies that strengthens with dye size, demonstrating that short-range attractive interactions can overcome electrostatic repulsion between cationic dyes and support stable aggregates. Water-model comparisons show that diffusion and interfacial roughness are sensitive to temperature and to whether nominally rigid models are run with flexible water regime (which can substantially slow translational mobility). But these parameters do not change the qualitative picture of interfacial accumulation and preference toward “horizontal” orientation of TAM dyes at the interface. Additionally, we computed ensemble-averaged TDDFT spectra from MD snapshots using explicit local solvation shells. We observed that the spectra are strongly dependent on dye parametrization type. Under the symmetric parametrization, bulk and interfacial spectra are broadly similar and show no systematic shift, whereas the non-negligible bulk-interface shifts appear with the quinone-like parametrization.

## Supplementary Material



## Notes

Additional analysis and simulation data; a complete summary of all single-dyed simulations performed in this study (Table S1); distributions of the orientation angles between the dye molecular plane normal and the surface normal (Figure S1–S16); MSD curves (Figures S17–S29); and density profiles (Figures S30–S36); dihedral analyses using defined atomic numbering and comparison of bulk vs. interface (Figures S37–S41), along with counterion-dye distance and counterion spatial probability distributions (Figures S42–S43); ensemble-averaged COM water-corrected z-coordinate distributions over 100 trajectories for dyes using OPC3-pol-FW/A (300 K), OPC3-FW/A (300 K), OPC3-FW/A (400 K), OPC3/B (300 K), and fits (Figures S44–S49); normalized probability distributions of the H–O–H bond angle and O–H bond length­(Figures S52 and S53); representative snapshots from XTB microhydration and multidyed slab simulation (Figures S51 and S54) (PDF)
