# Supplementary material for: Microhydration and Interfacial Activity of Triarylmethane Dyes at the Air–Water Interface
Source: J Phys Chem B. 2026 May 19;130(22):5734–48. doi: 10.1021/acs.jpcb.6c01352 (PMC13244440; doi:10.1021/acs.jpcb.6c01352)
Supplement: Supplementary file 1 [file jp6c01352_si_001.pdf]

Supporting Information for Microhydration and Interfacial Activity of Triarylmethane Dyes at the Air-Water Interface

S. K. A. Ishini Madushika Sooriyabandara,[a] *Nikolay V. Tkachenko*[*,a,b]

[a] Department of Chemistry and Biochemistry, University of Oklahoma, Norman, Oklahoma 73019, USA.

[b] Materials Science and Engineering Program, University of Oklahoma, Norman, Oklahoma 73019, USA.

Table S1. Summary table of single dye slab systems simulated in this work.

Systems	Water model	Production (ns)	Temperature (K)	Replica
**Slab**<br><br>TAM0<br>TAM1<br>TAM2<br>TAM3<br>TAM3I	OPC3 -FW	30<br>10<br>15	400, 300<br>400<br>300	3<br>100<br>100
	OPC3	30<br>10	300	1<br>100
	OPC3-pol	30	300	1
	OPC3-pol-FW	30<br>15	300	1<br>100
	TIP3P -FW	30	400, 300	1
	SPC-FW	30	300	1
**Bulk**<br><br>TAM0<br>TAM1<br>TAM2<br>TAM3<br>TAM3I	OPC3 - FW	10	400, 300	3
	OPC3	10	300	2
	OPC3-pol - FW	10	300	1
	OPC3-pol	10	300	1
	TIP3P - FW	10	400, 300	1
	SPC-FW	10	300	1

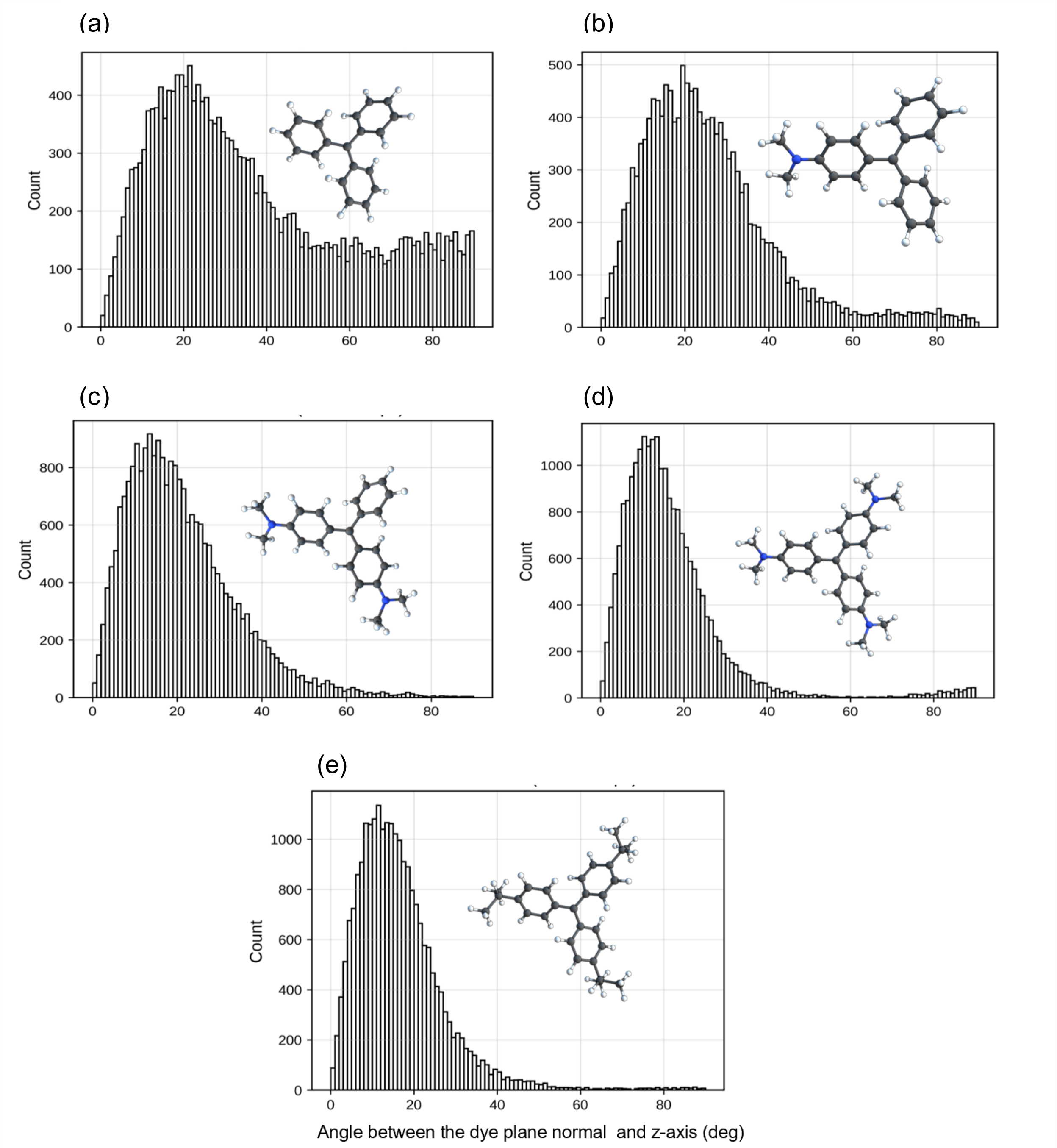

Figure S1. Probability distributions of the orientation angle between the dye molecular plane normal and the surface normal (z-axis) for (a) TAM0, (b) TAM1, (c) TAM2, (d) TAM3, and (e) TAM3I obtained from single-dye slab MD simulations at 300 K using the OPC3-FW. Only trajectory frames after interfacial adsorption were included in the analysis windows.

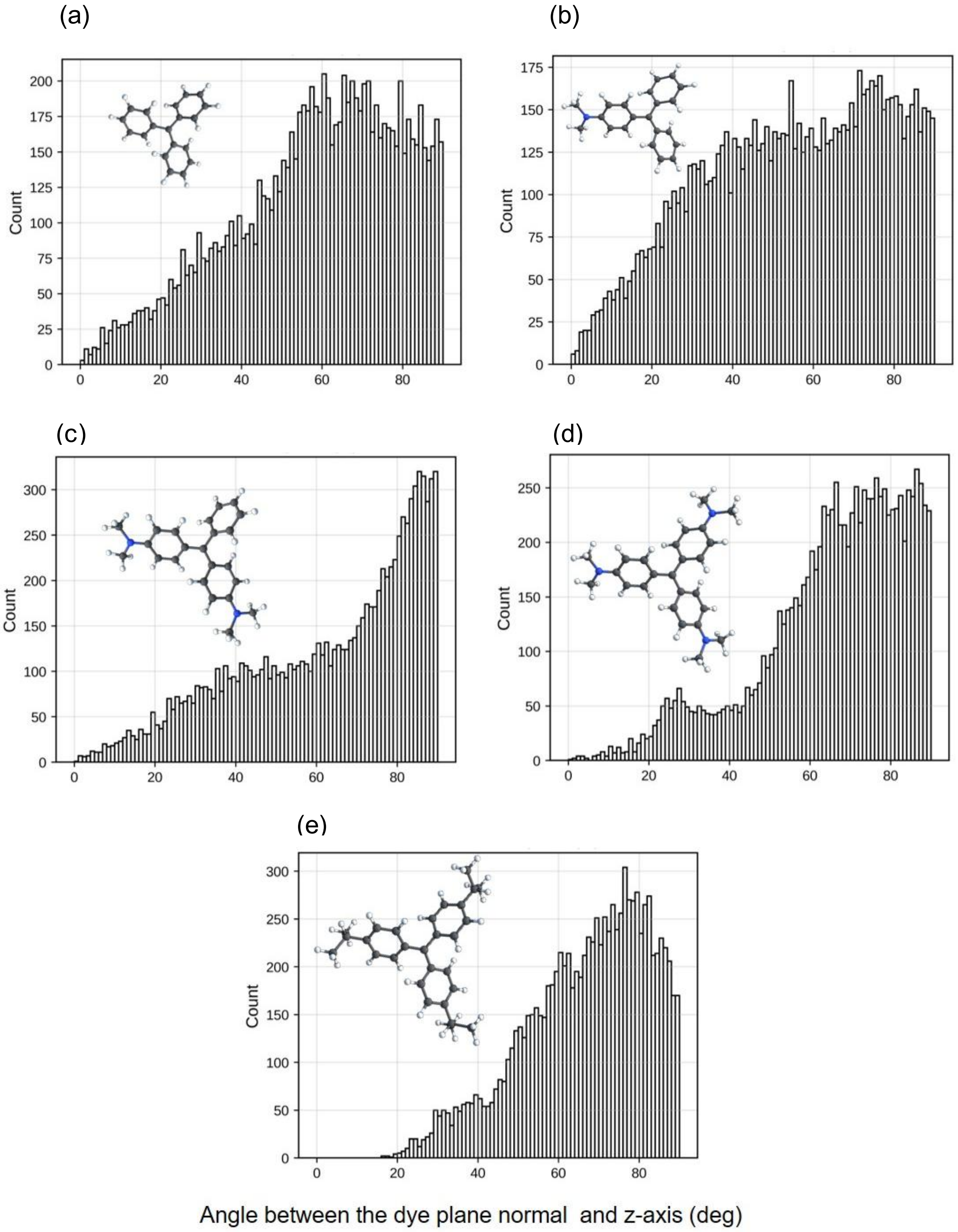

Figure S2. Probability distributions of the orientation angle between the dye molecular plane normal and the surface normal (z-axis) for (a) TAM0, (b) TAM1, (c) TAM2, (d) TAM3, and (e) TAM3I obtained from single-dye bulk MD simulations at 300 K using the OPC3-FW.

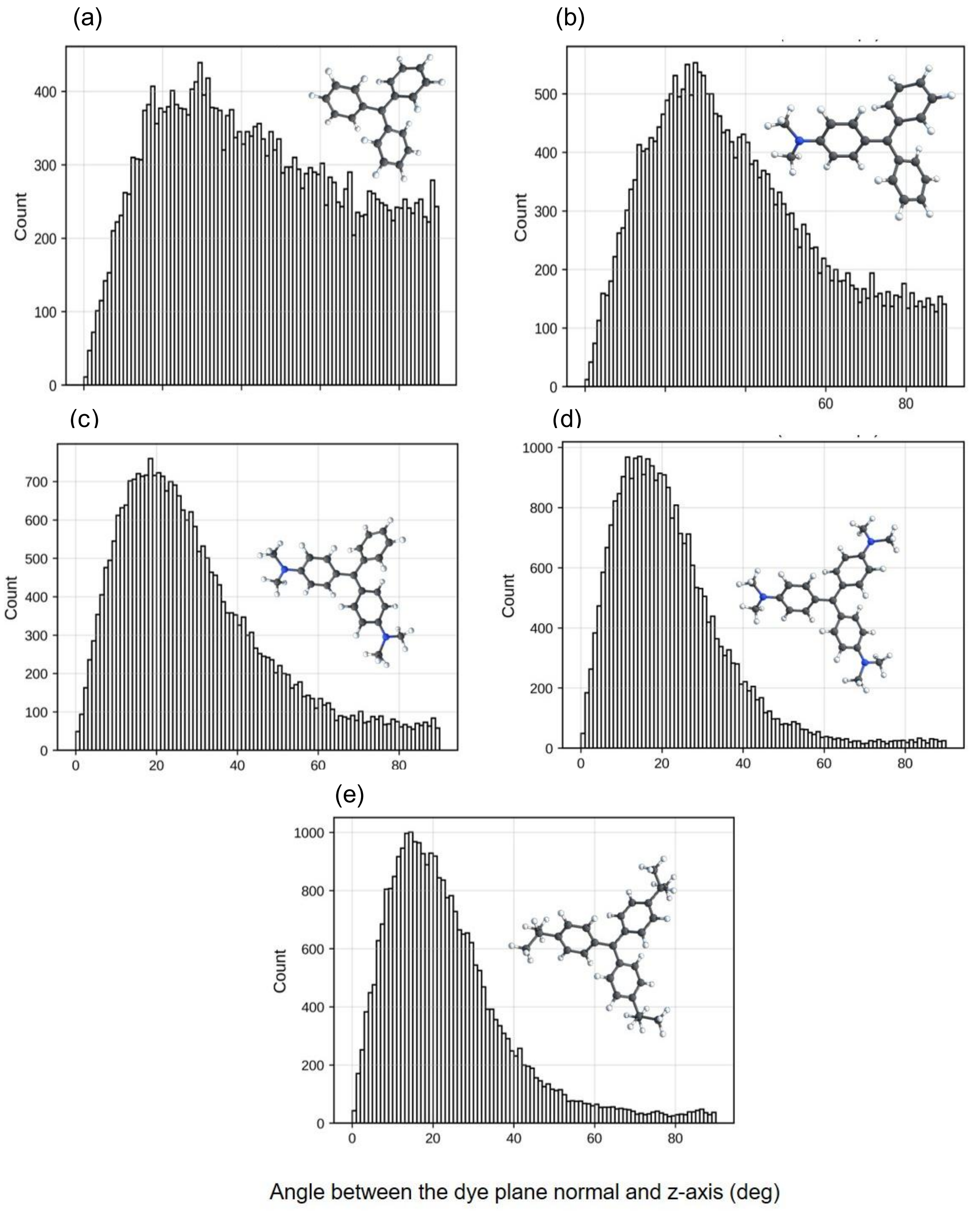

Figure S3. Probability distributions of the orientation angle between the dye molecular plane normal and the surface normal (z-axis) for (a) TAM0, (b) TAM1, (c) TAM2, (d) TAM3, and (e) TAM3I obtained from single-dye slab MD simulations at 400 K using the OPC3-FW. Only trajectory frames after interfacial adsorption were included in the analysis windows.

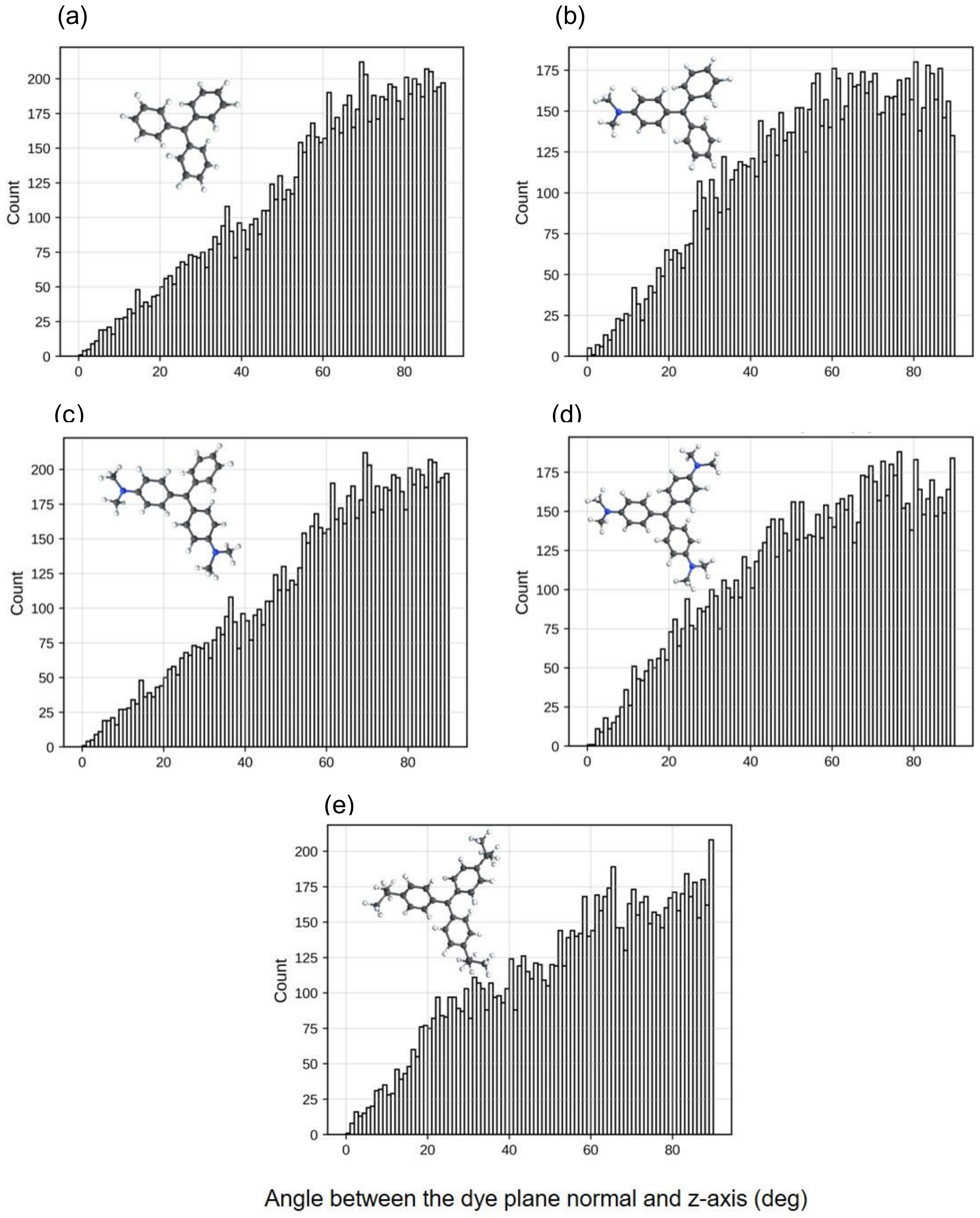

Figure S4. Probability distributions of the orientation angle between the dye molecular plane normal and the surface normal (z-axis) for (a) TAM0, (b) TAM1, (c) TAM2, (d) TAM3, and (e) TAM3I obtained from single-dye bulk MD simulations at 400 K using OPC3-FW.

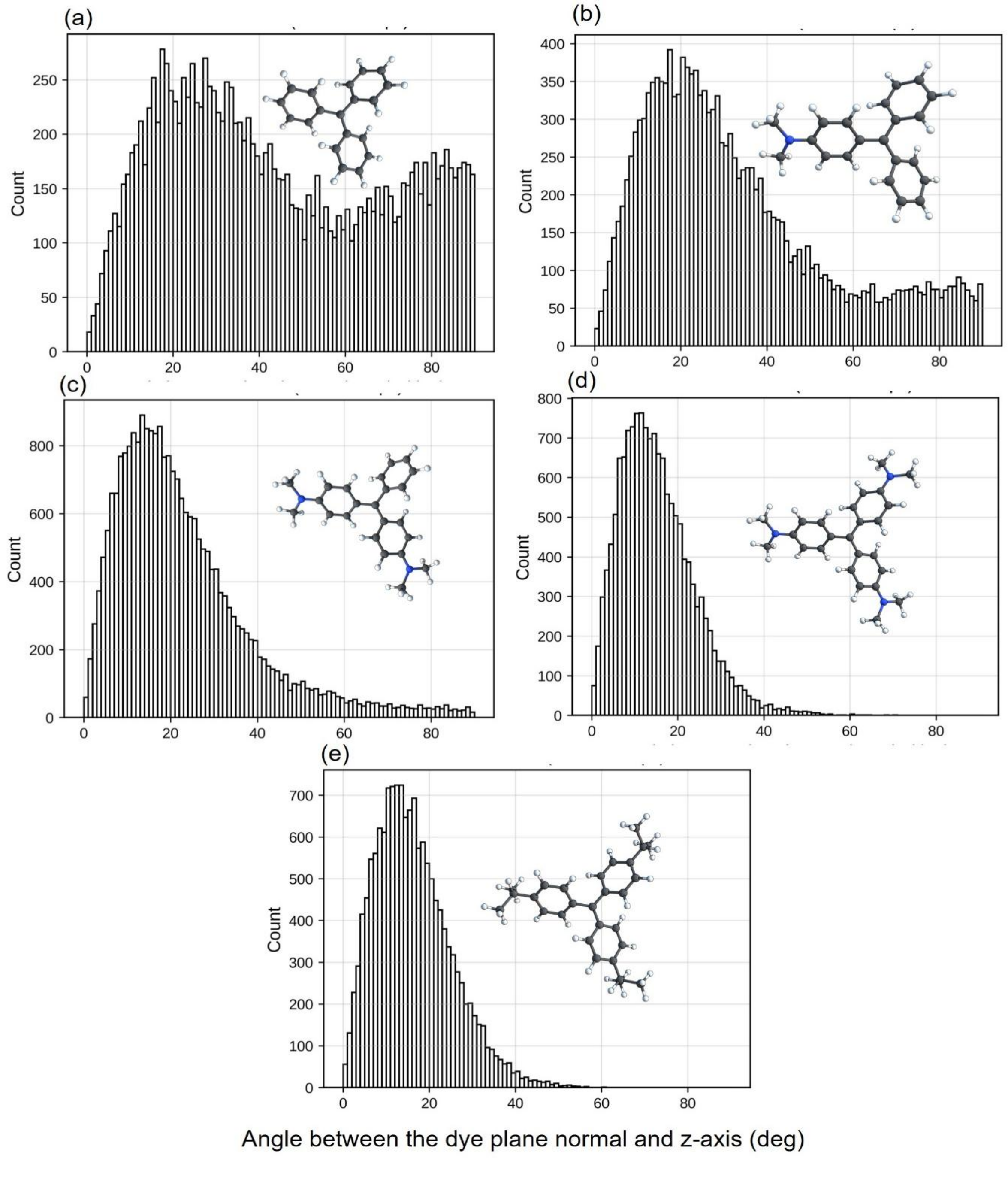

Figure S5. Probability distributions of the orientation angle between the dye molecular plane normal and the surface normal (z-axis) for (a) TAM0, (b) TAM1, (c) TAM2, (d) TAM3, and (e) TAM3I obtained from the single-dye slab at 300 K using OPC3-pol-FW. Only trajectory frames after interfacial adsorption were included in the analysis windows.

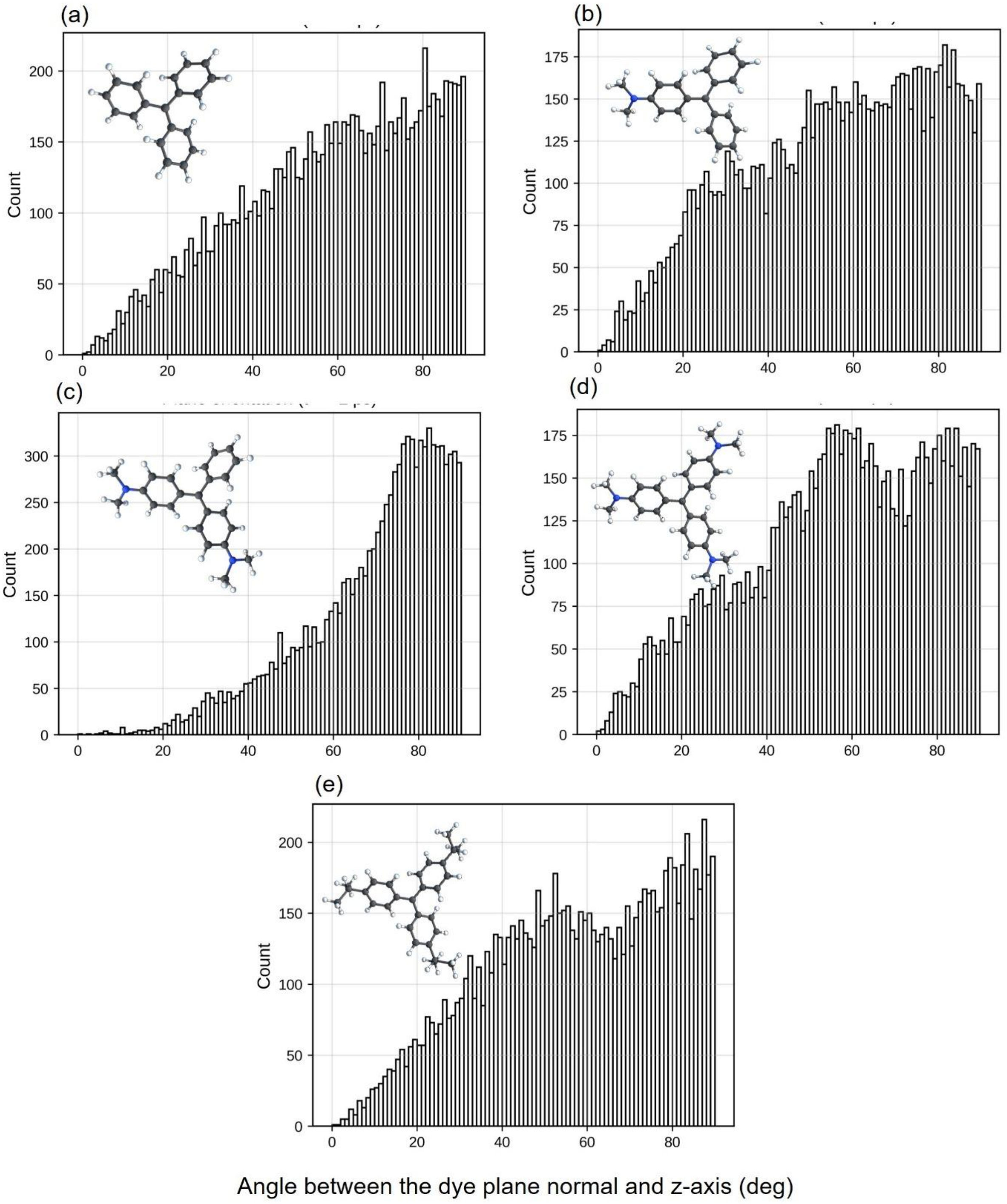

Figure S6. Probability distributions of the orientation angle between the dye molecular plane normal and the surface normal (z-axis) for (a) TAM0, (b) TAM1, (c) TAM2, (d) TAM3, and (e) TAM3I obtained from single-dye bulk MD simulations at 300 K using the OPC3-pol-FW.

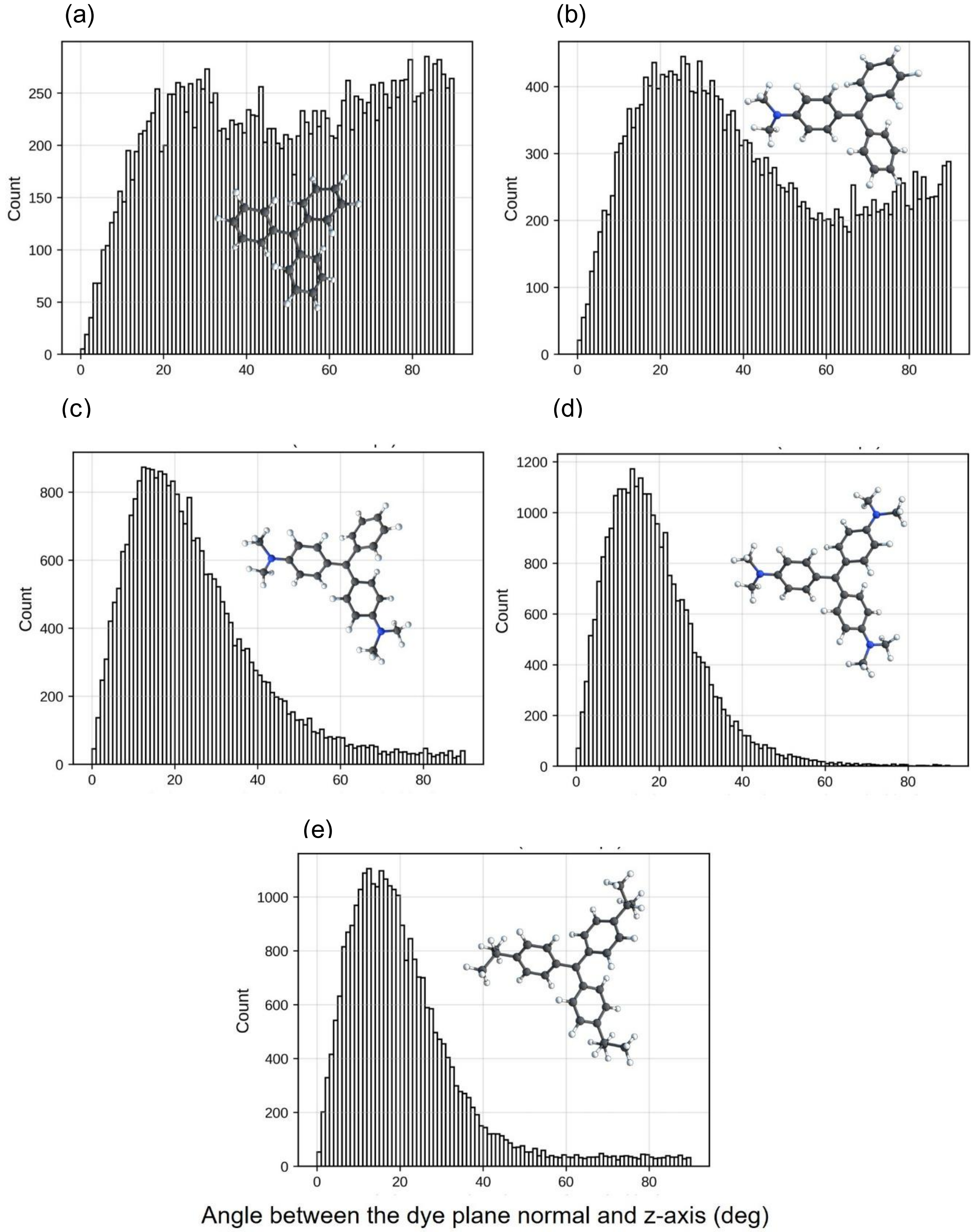

Figure S7. Probability distributions of the orientation angle between the dye molecular plane normal and the surface normal (z-axis) for (a) TAM0, (b) TAM1, (c) TAM2, (d) TAM3, and (e) TAM3I obtained from single-dye slab MD simulations at 300 K using the TIP3P-FW. Only trajectory frames after interfacial adsorption were included in the analysis windows

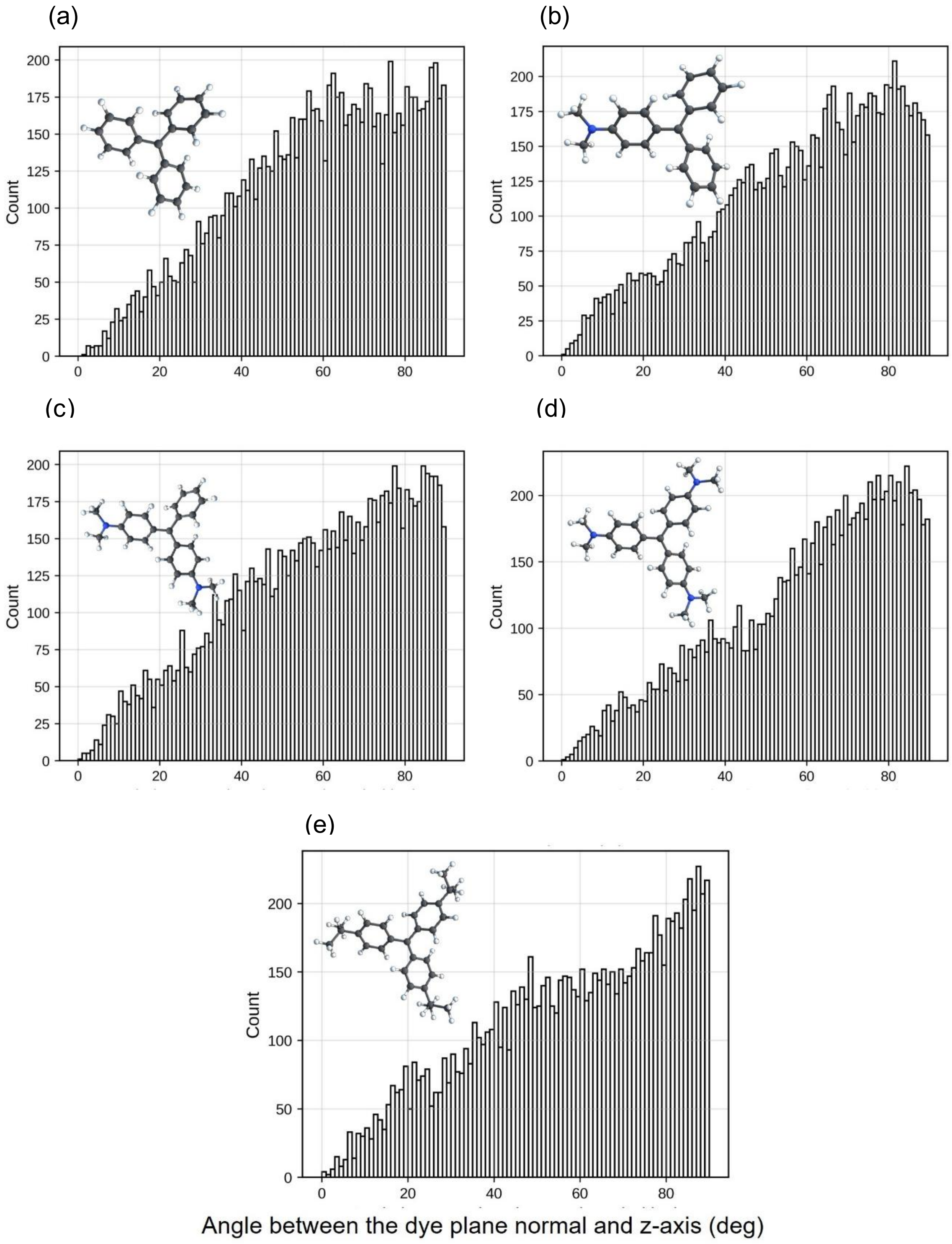

Figure S8. Probability distributions of the orientation angle between the dye molecular plane normal and the surface normal (z-axis) for (a) TAM0, (b) TAM1, (c) TAM2, (d) TAM3, and (e) TAM3I obtained from single-dye bulk MD simulations at 300 K using the TIP3P-FW.

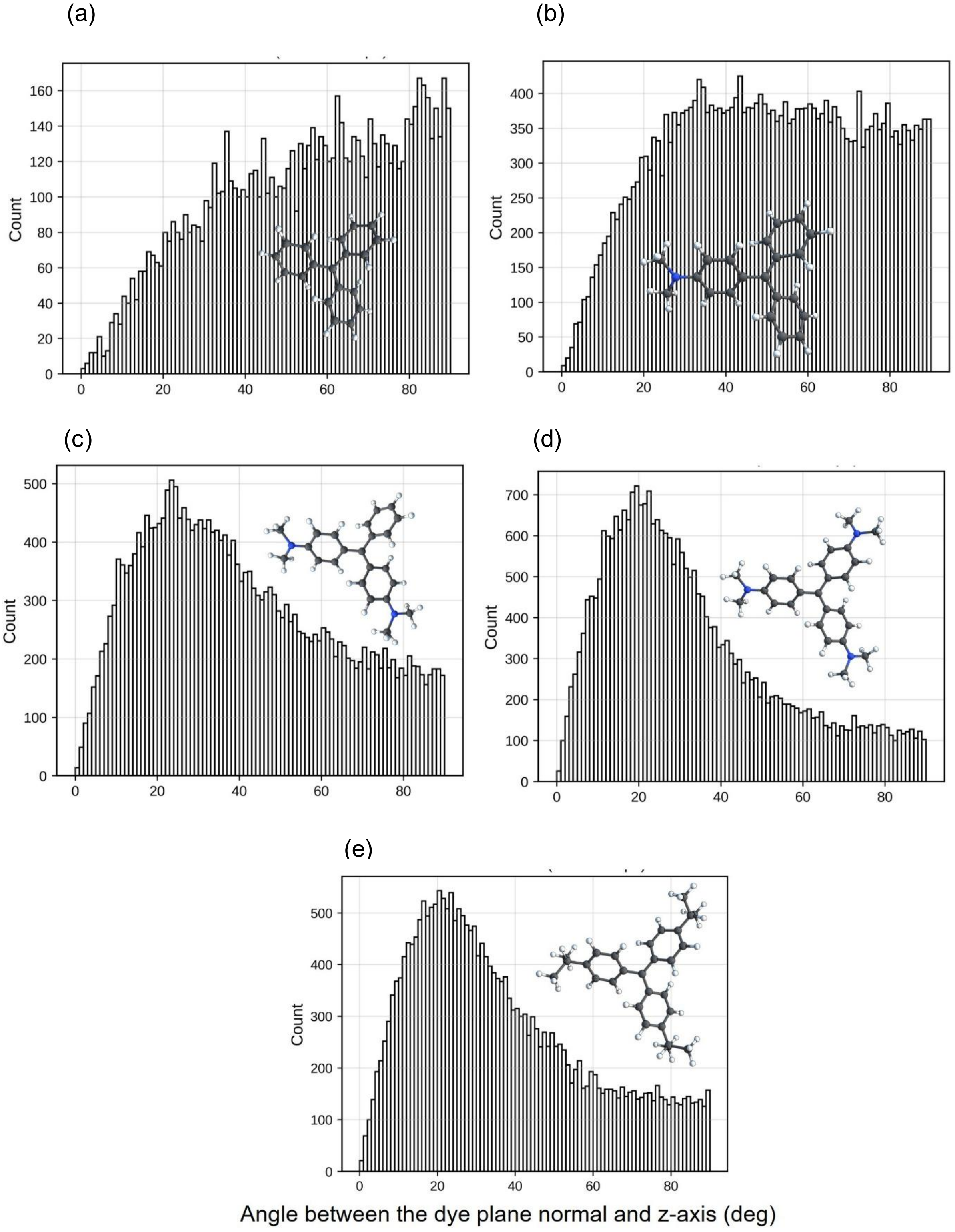

Figure S9. Probability distributions of the orientation angle between the dye molecular plane normal and the surface normal (z-axis) for (a) TAM0, (b) TAM1, (c) TAM2, (d) TAM3, and (e) TAM3I obtained from single-dye slab MD simulations at 400 K using the TIP3P-FW. Only trajectory frames after interfacial adsorption were included in the analysis windows

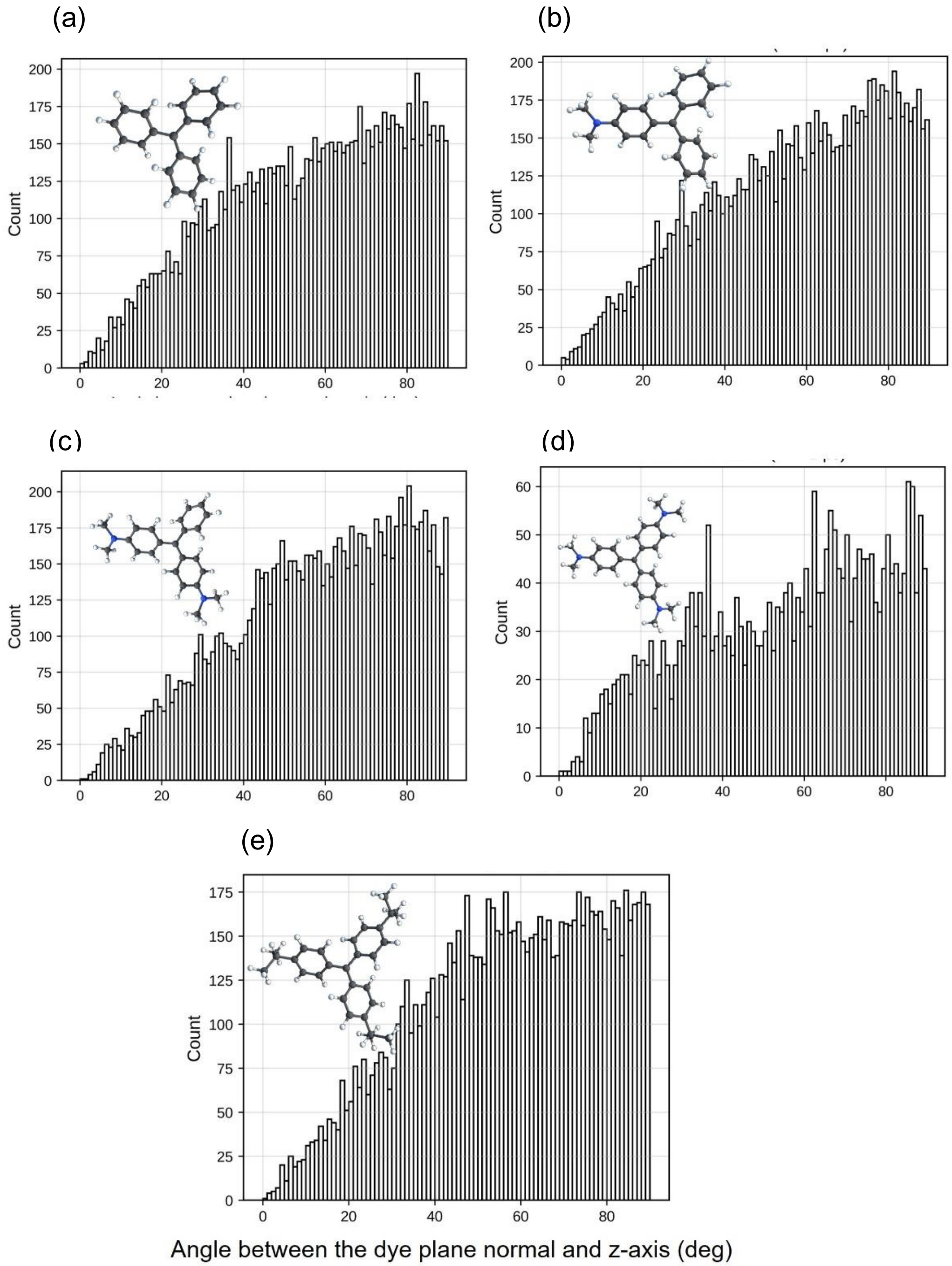

Figure S10. Probability distributions of the orientation angle between the dye molecular plane normal and the surface normal (z-axis) for (a) TAM0, (b) TAM1, (c) TAM2, (d) TAM3, and (e) TAM3I obtained from single-dye bulk MD simulations at 400 K using the TIP3P-FW.

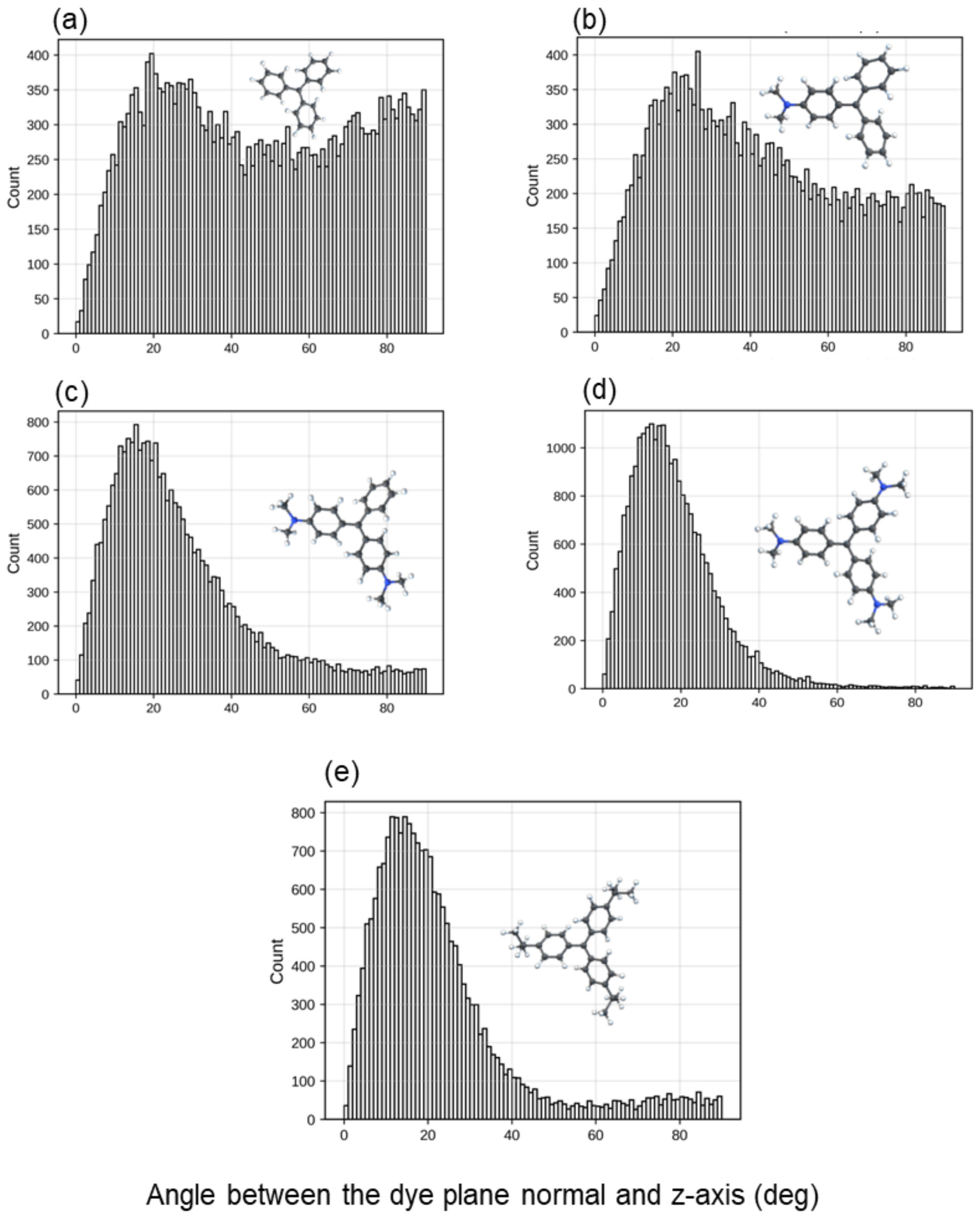

Figure S11. Probability distributions of the orientation angle between the dye molecular plane normal and the surface normal (z-axis) for (a) TAM0, (b) TAM1, (c) TAM2, (d) TAM3, and (e) TAM3I obtained from single-dye slab MD simulations at 300 K using the SPC-FW. Only trajectory frames after interfacial adsorption were included in the analysis windows

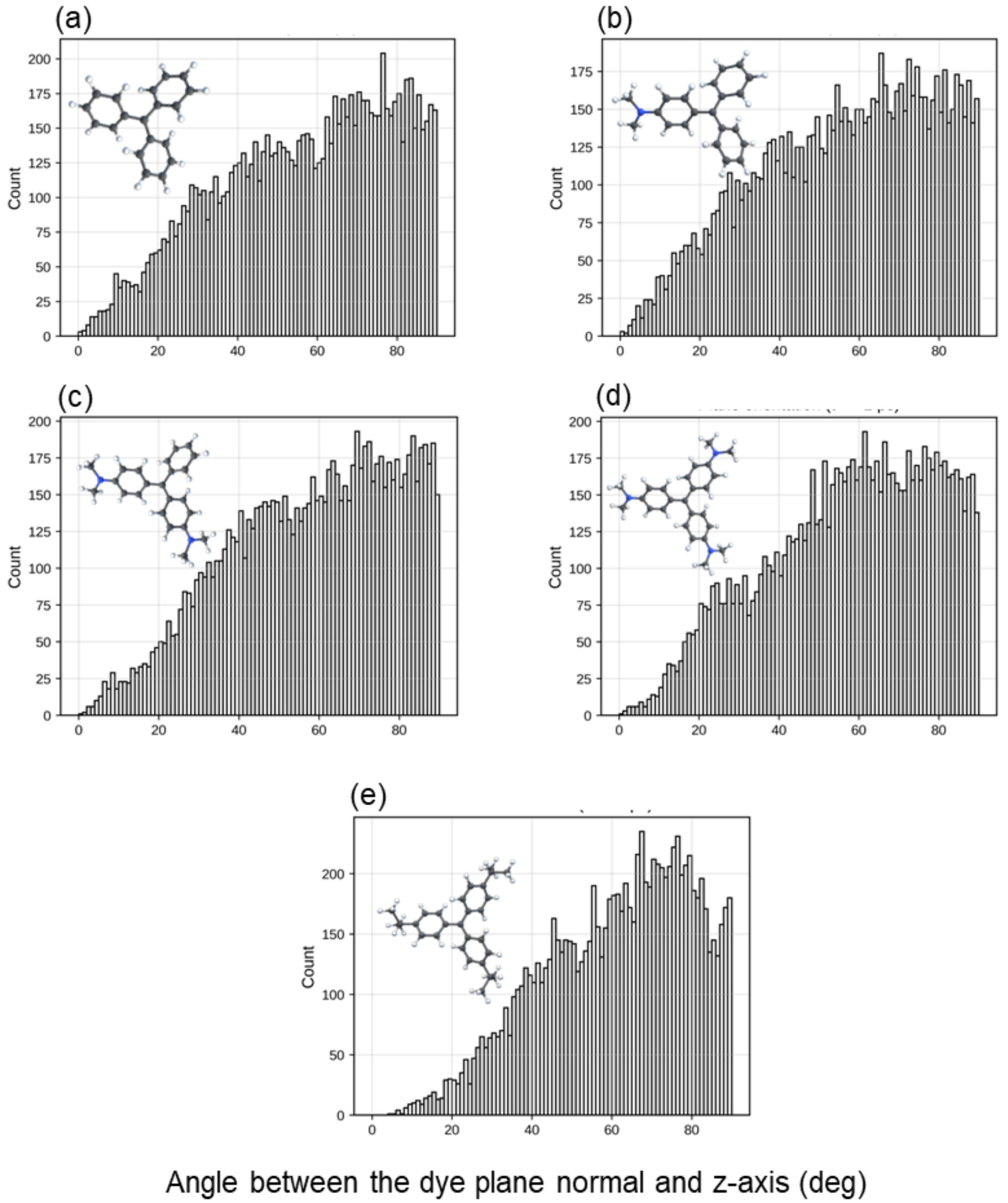

Figure S12. Probability distributions of the orientation angle between the dye molecular plane normal and the surface normal (z-axis) for (a) TAM0, (b) TAM1, (c) TAM2, (d) TAM3, and (e) TAM3I obtained from single-dye bulk MD simulations at 300 K using the SPC-FW.

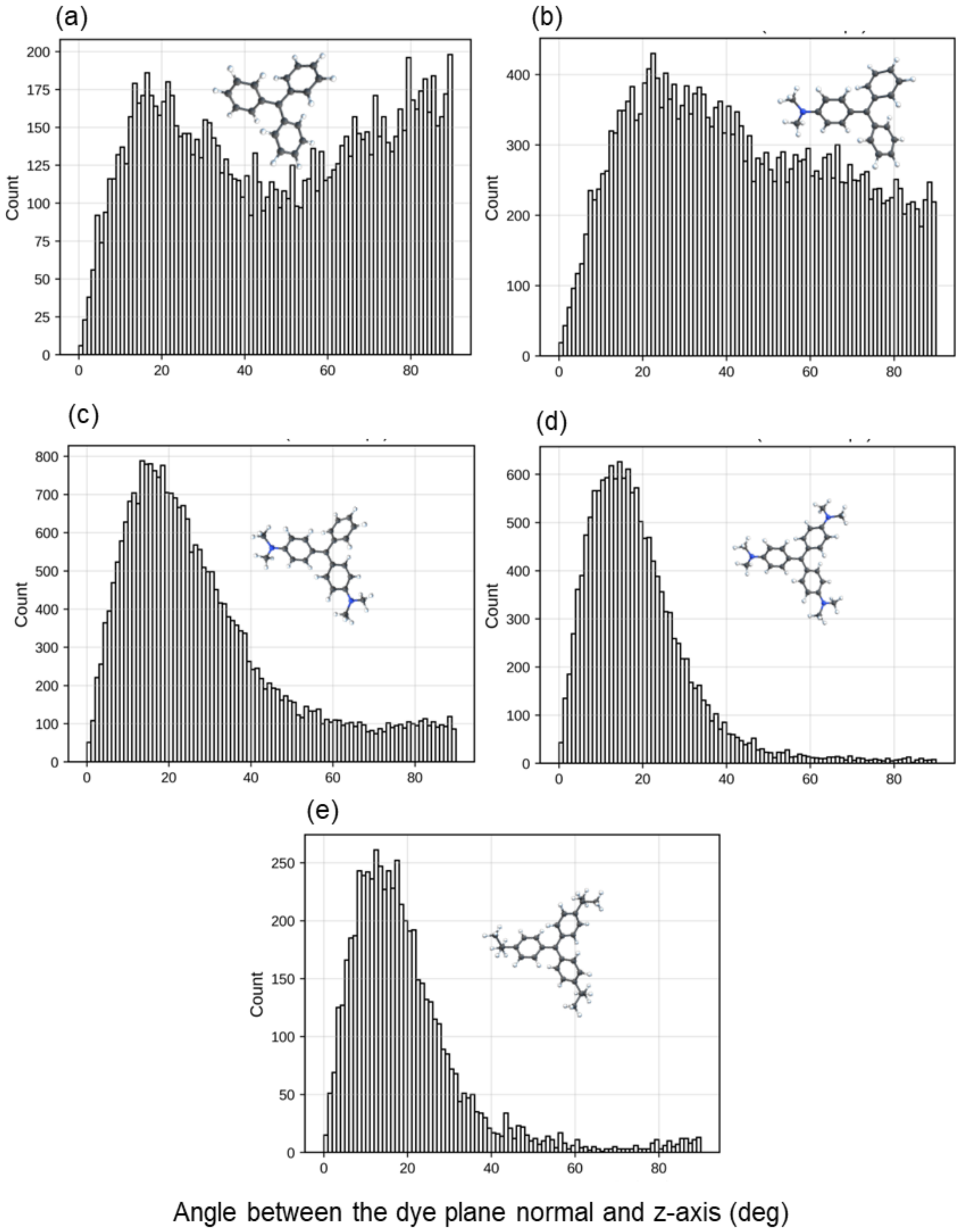

Figure S13. Probability distributions of the orientation angle between the dye molecular plane normal and the surface normal (z-axis) for (a) TAM0, (b) TAM1, (c) TAM2, (d) TAM3, and (e) TAM3I obtained at 300 K using the OPC3. Only trajectory frames after interfacial adsorption were included in the analysis windows

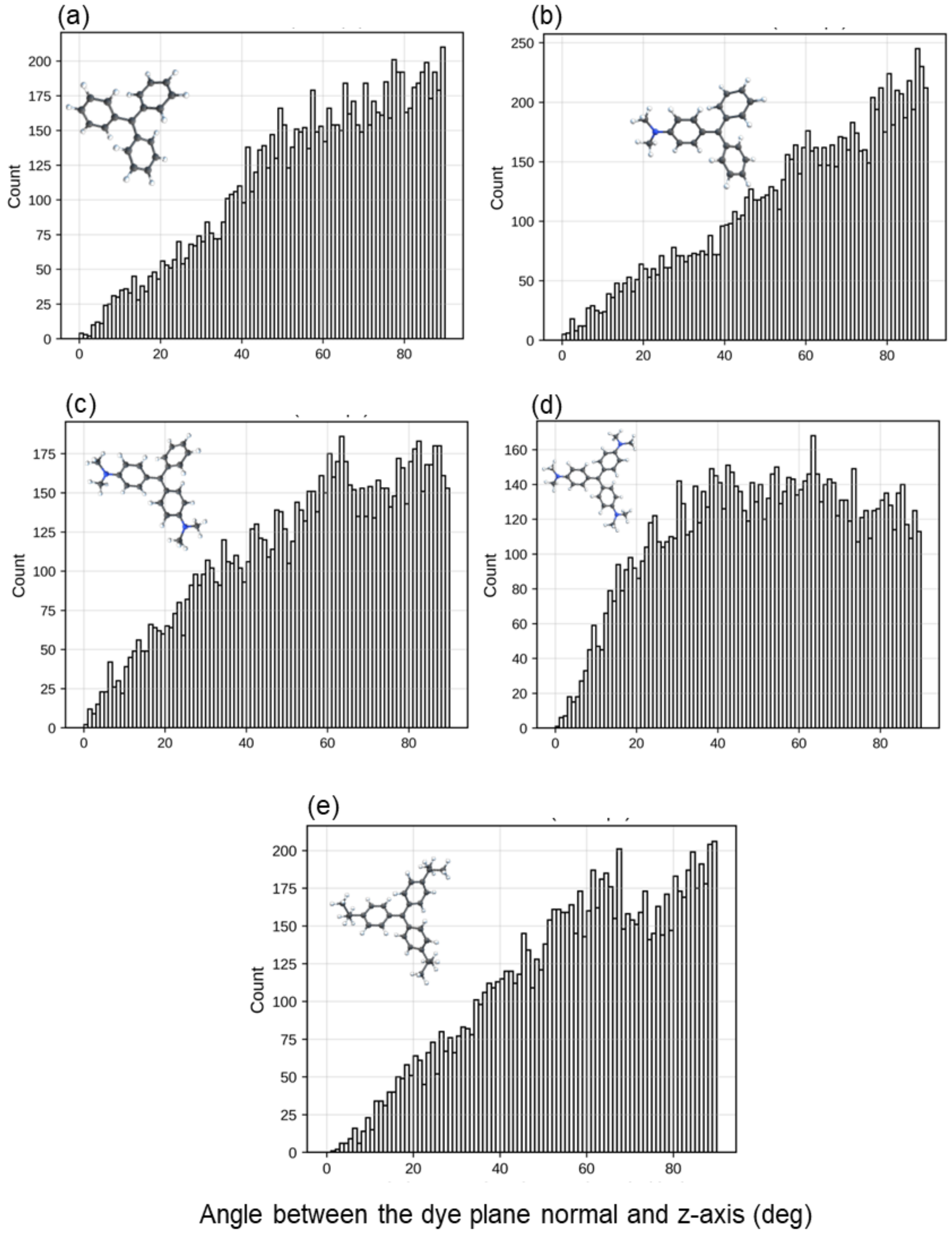

Figure S14. Probability distributions of the orientation angle between the dye molecular plane normal and the surface normal (z-axis) for (a) TAM0, (b) TAM1, (c) TAM2, (d) TAM3, and (e) TAM3I obtained from single-dye bulk MD simulations at 300 K using the OPC3.

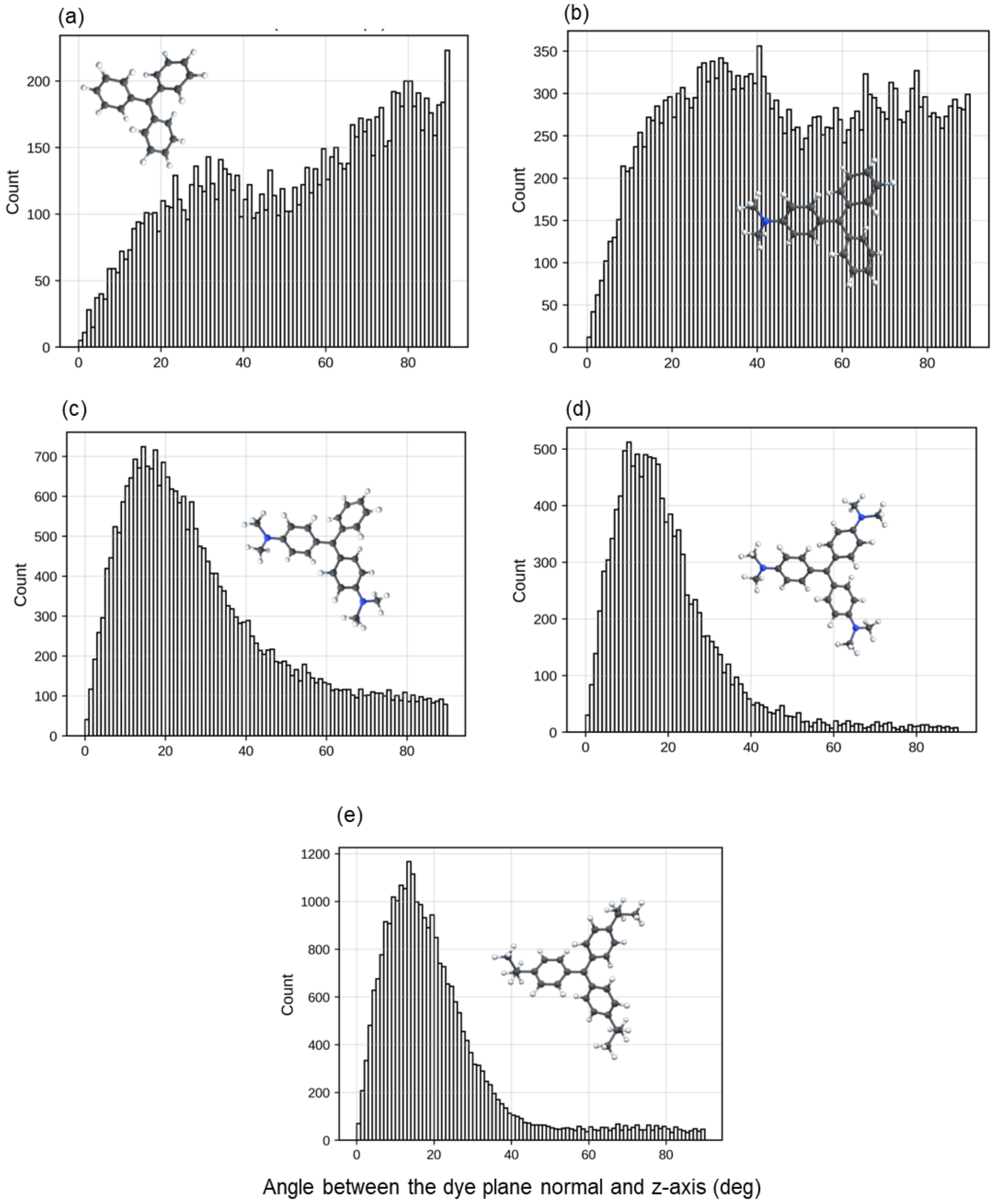

Figure S15 Probability distributions of the orientation angle between the dye molecular plane normal and the surface normal (z-axis) for (a) TAM0, (b) TAM1, (c) TAM2, (d) TAM3, and (e) TAM3I obtained from single-dye slab MD simulations at 300 K using the OPC3-pol. Only trajectory frames after interfacial adsorption were included in the analysis.

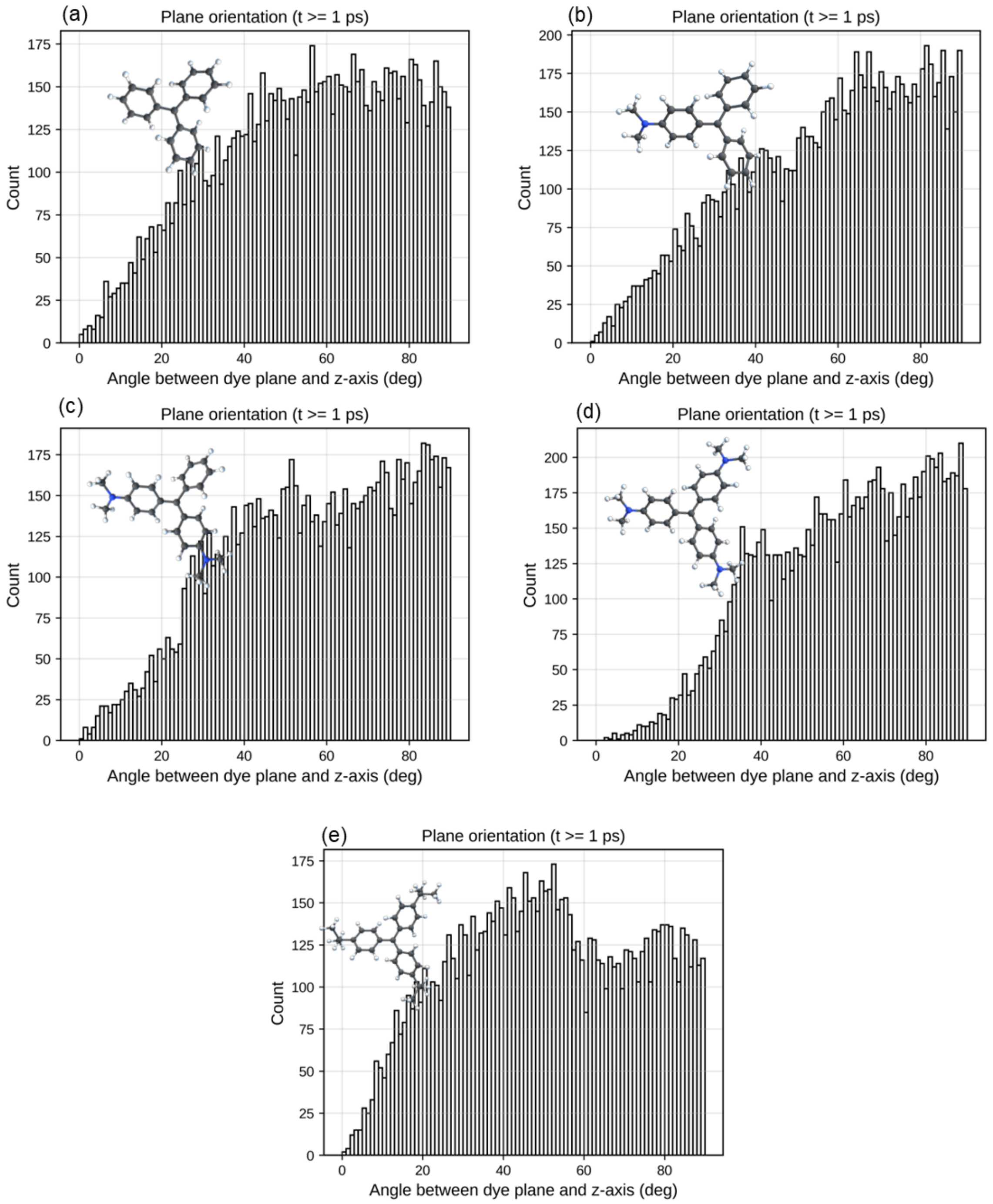

Figure S16. Probability distributions of the orientation angle between the dye molecular plane normal and the surface normal (z-axis) for (a) TAM0, (b) TAM1, (c) TAM2, (d) TAM3, and (e) TAM3I obtained from single-dye bulk MD simulations at 300 K using the OPC3-pol.

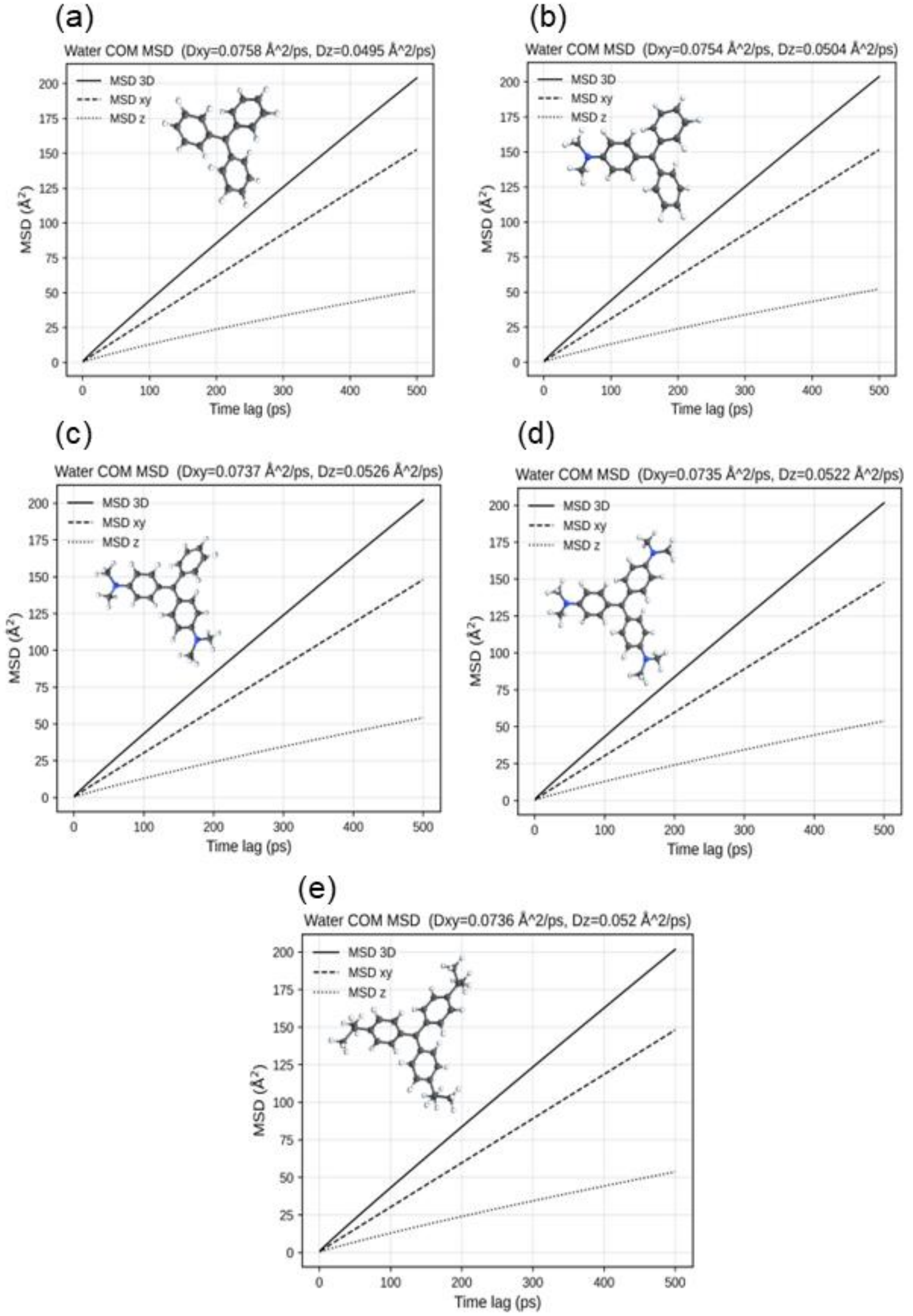

Figure S17. MSD curves over the diffusive time window computed from water-molecule center-of-mass trajectories of the dyes (a) TAM0, (b) TAM1, (c) TAM2, (d) TAM3, and (e) TAM3I from single-dye slab MD simulations at 300 K using the OPC3-FW.

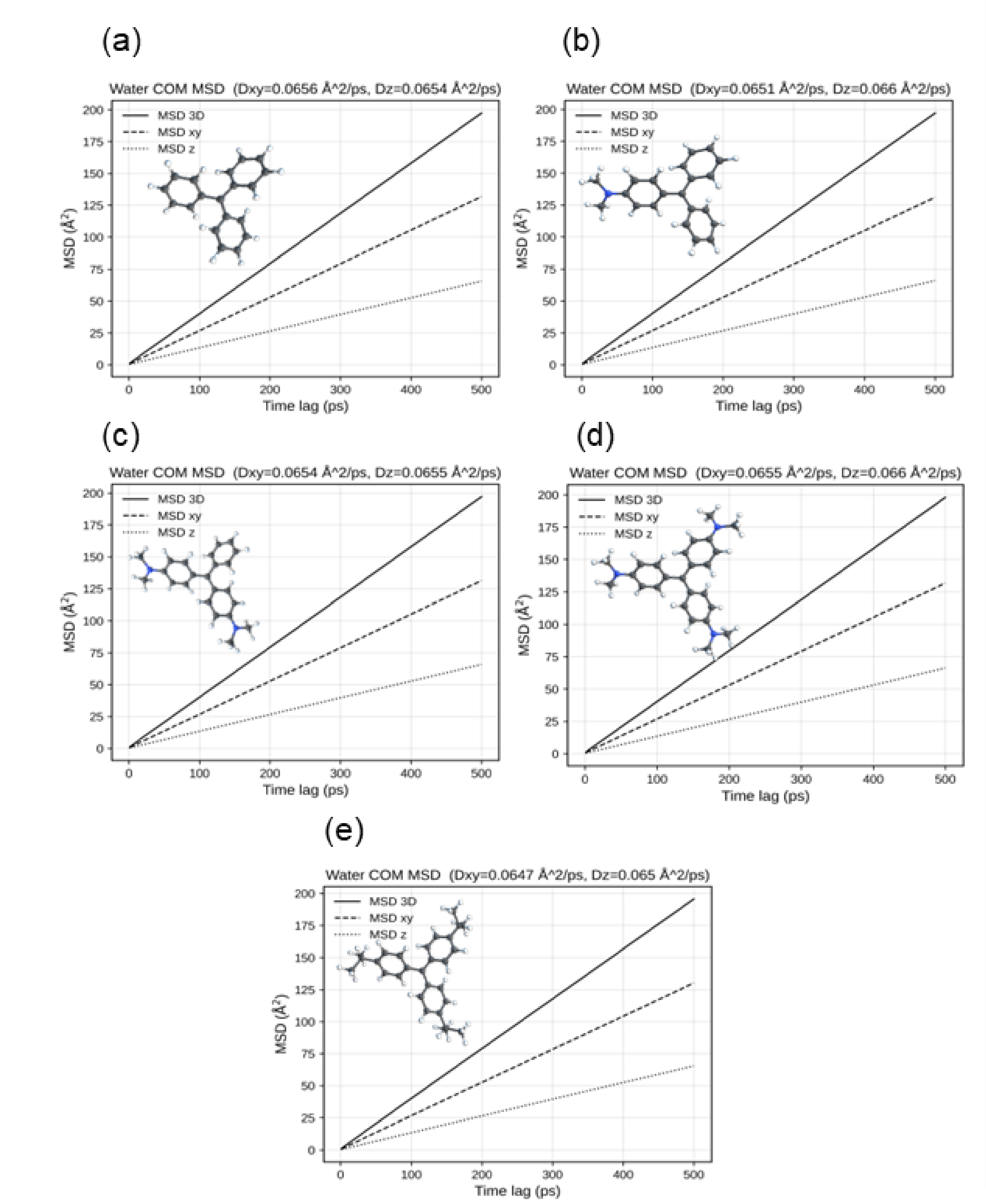

Figure S18. MSD curves over the diffusive time window computed from water COM trajectories of the dyes (a) TAM0, (b) TAM1, (c) TAM2, (d) TAM3, and (e) TAM3I from single-dye bulk MD simulations at 300 K using the OPC3-FW.

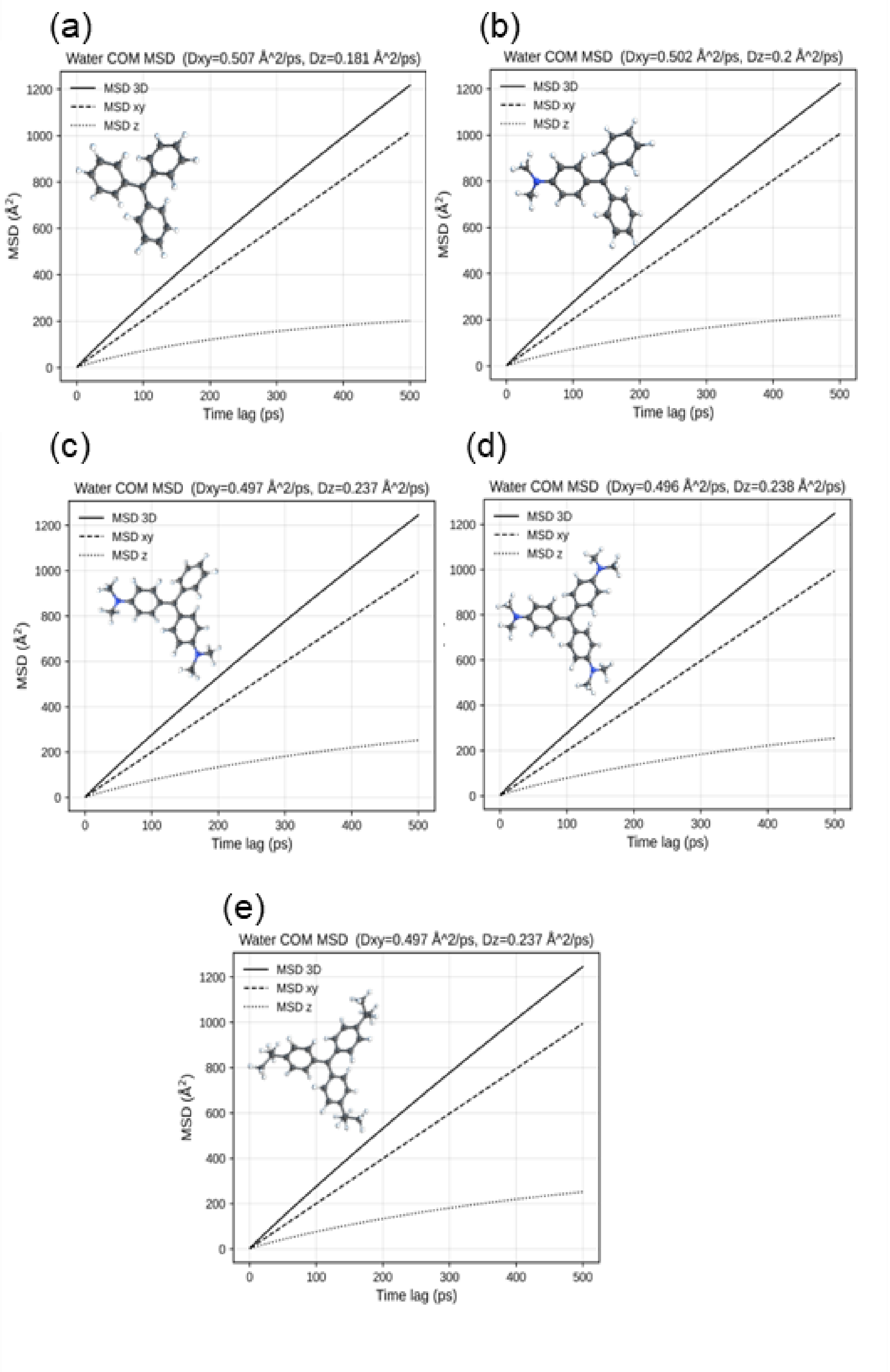

Figure S19. MSD curves over the diffusive time window computed from water COM trajectories of the dyes (a) TAM0, (b) TAM1, (c) TAM2, (d) TAM3, and (e) TAM3I from single-dye slab MD simulations at 400 K using the OPC3-FW.

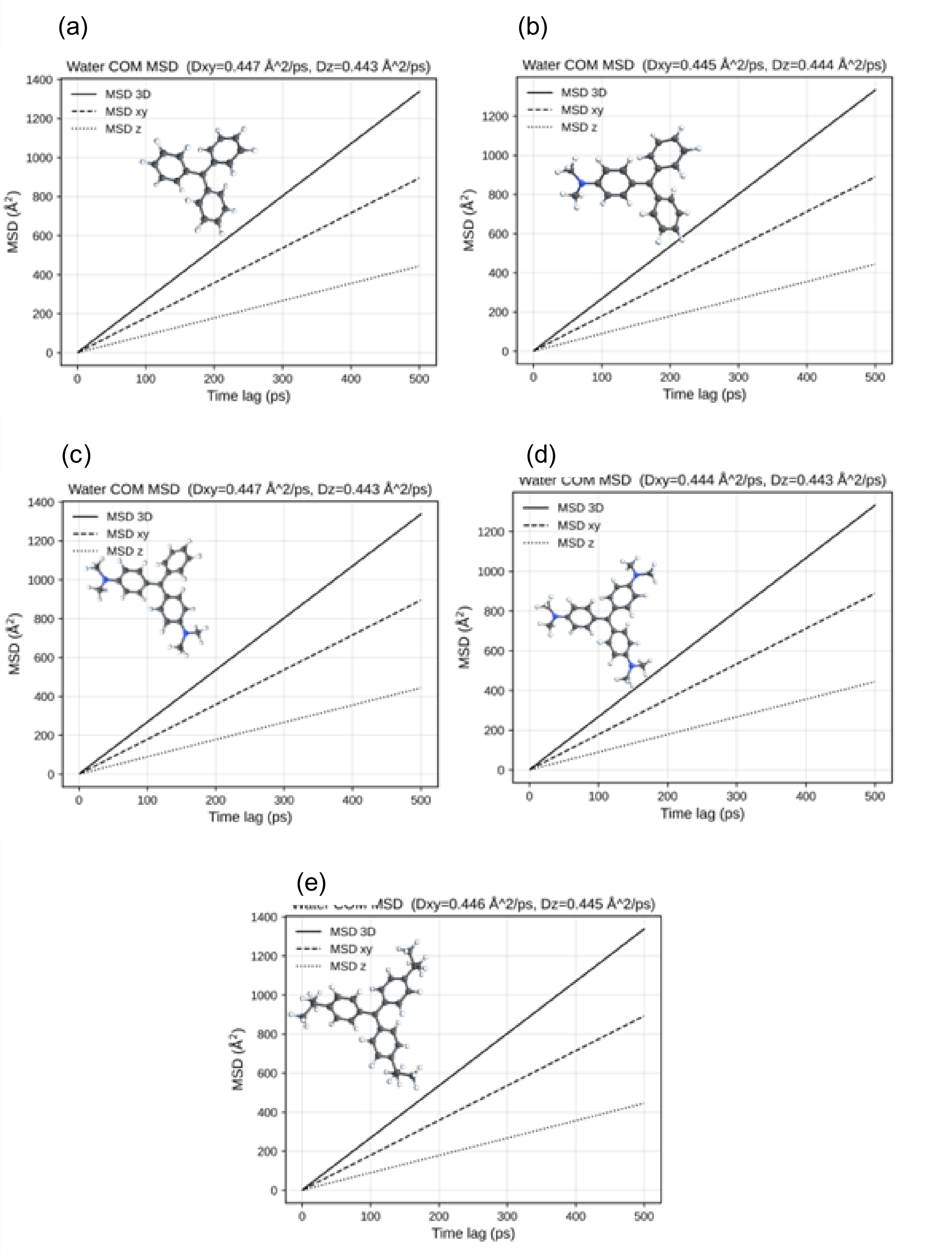

Figure S20. MSD curves over the diffusive time window computed from water COM trajectories of the dyes (a) TAM0, (b) TAM1, (c) TAM2, (d) TAM3, and (e) TAM3I from single-dye bulk MD simulations at 400 K using the OPC3-FW

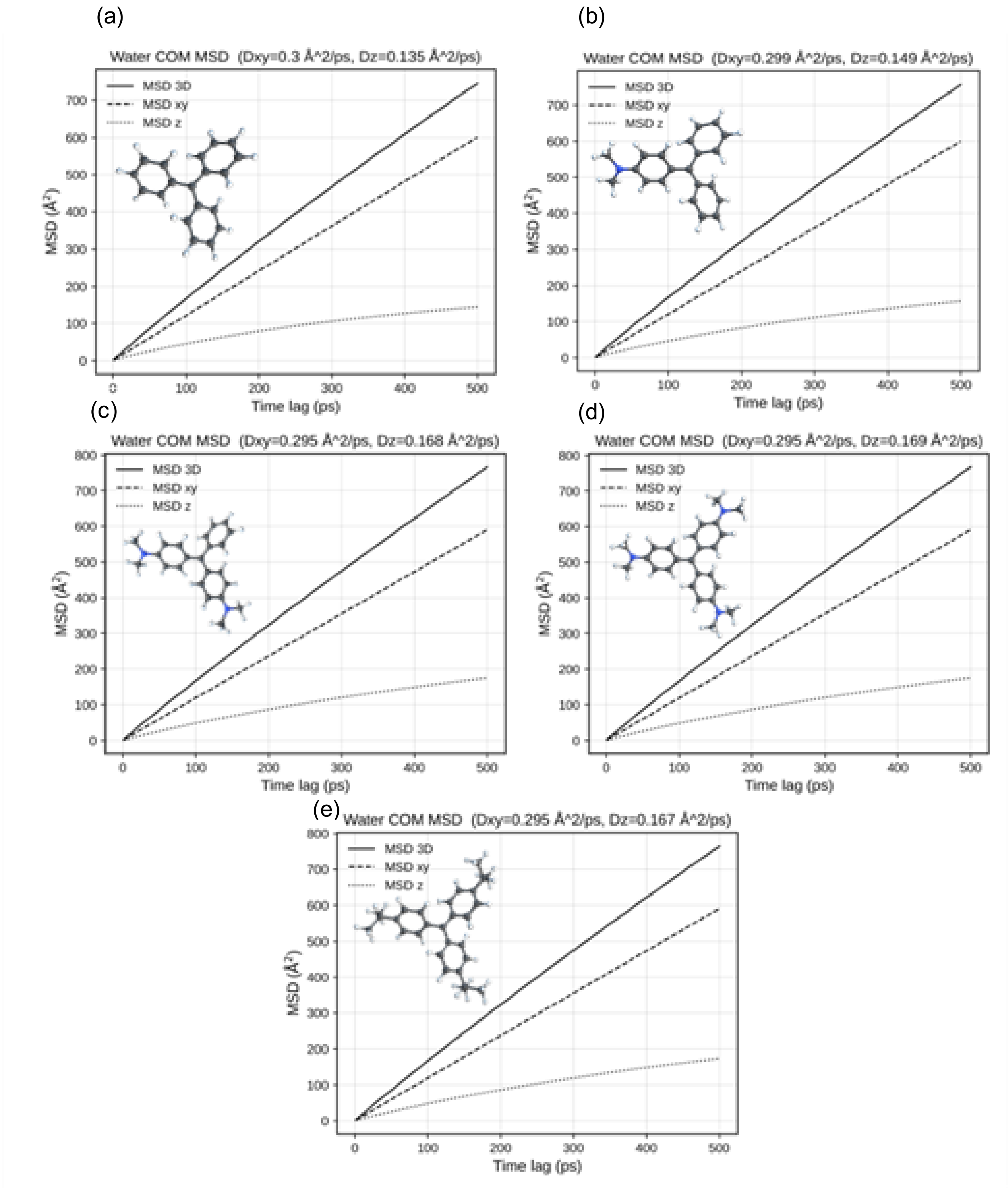

Figure S21. MSD curves over the diffusive time window computed from water COM trajectories of the dyes (a) TAM0, (b) TAM1, (c) TAM2, (d) TAM3, and (e) TAM3I from single-dye slab MD simulations at 300 K using the TIP3P-FW

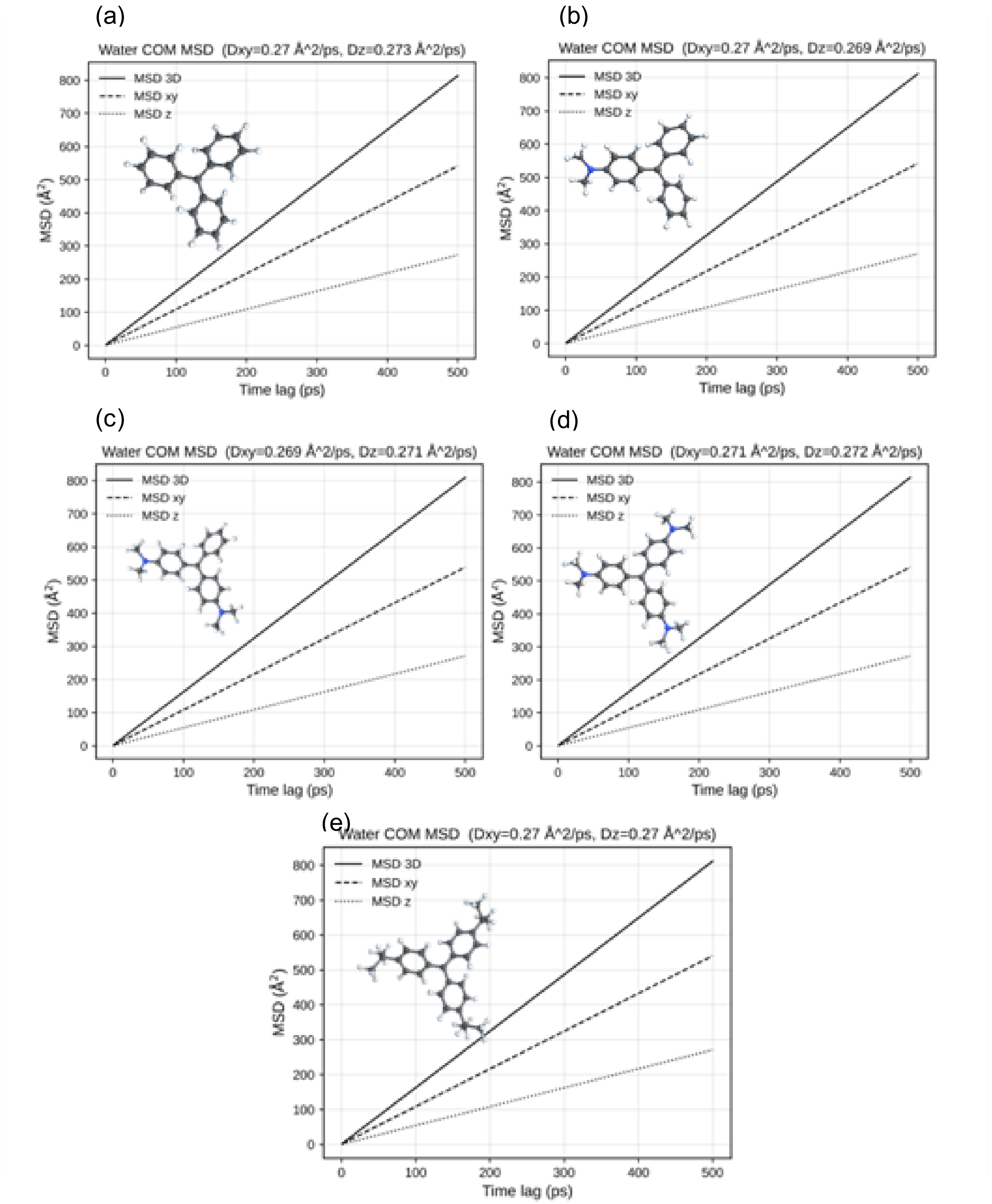

Figure S22. MSD curves over the diffusive time window computed from water COM trajectories of the dyes (a) TAM0, (b) TAM1, (c) TAM2, (d) TAM3, and (e) TAM3I from single-dye bulk MD simulations at 300 K using the TIP3P-FW

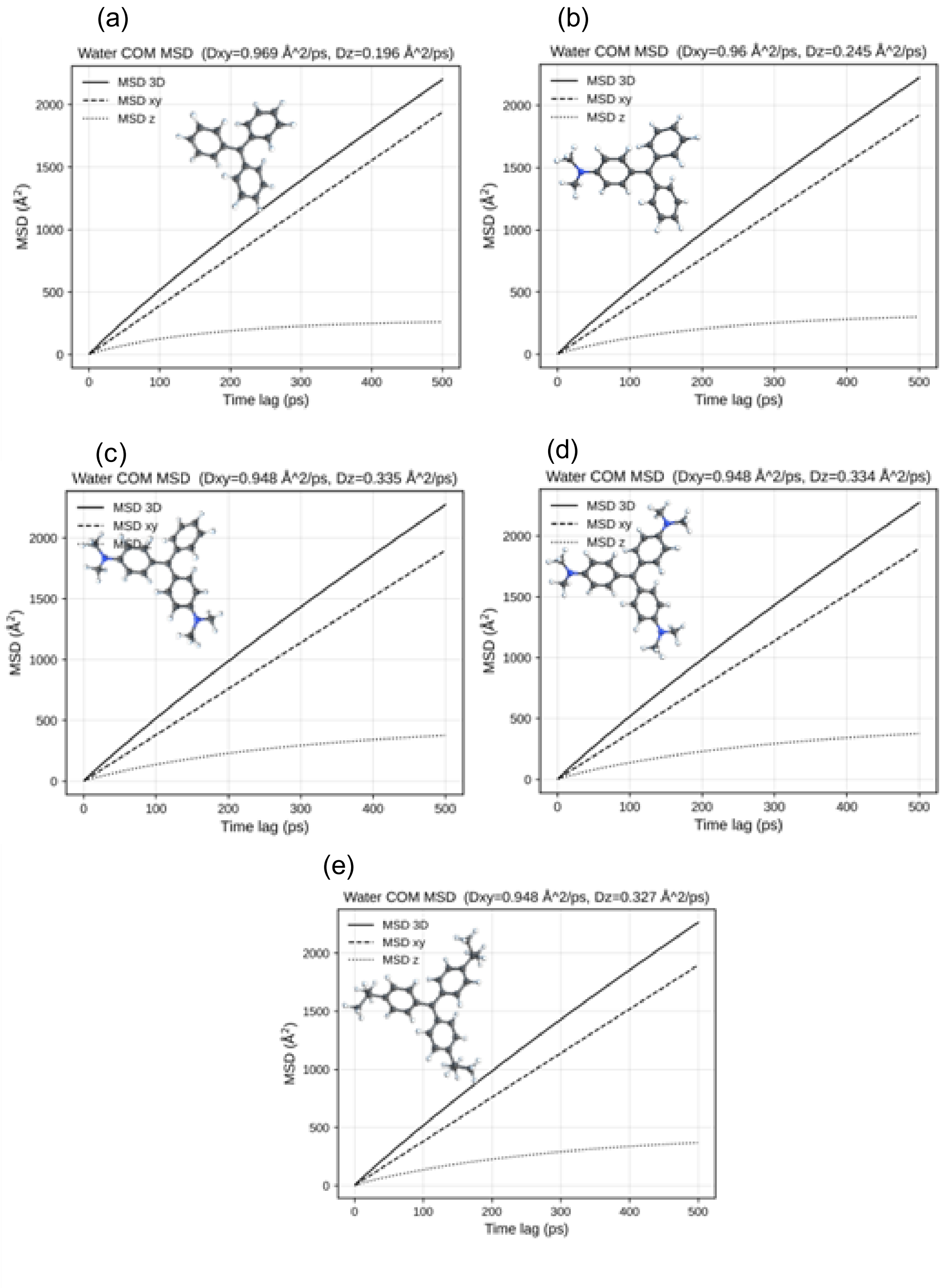

Figure S23. MSD curves over the diffusive time window computed from water COM trajectories of the dyes (a) TAM0, (b) TAM1, (c) TAM2, (d) TAM3, and (e) TAM3I from single-dye slab MD simulations at 400 K using the TIP3P-FW

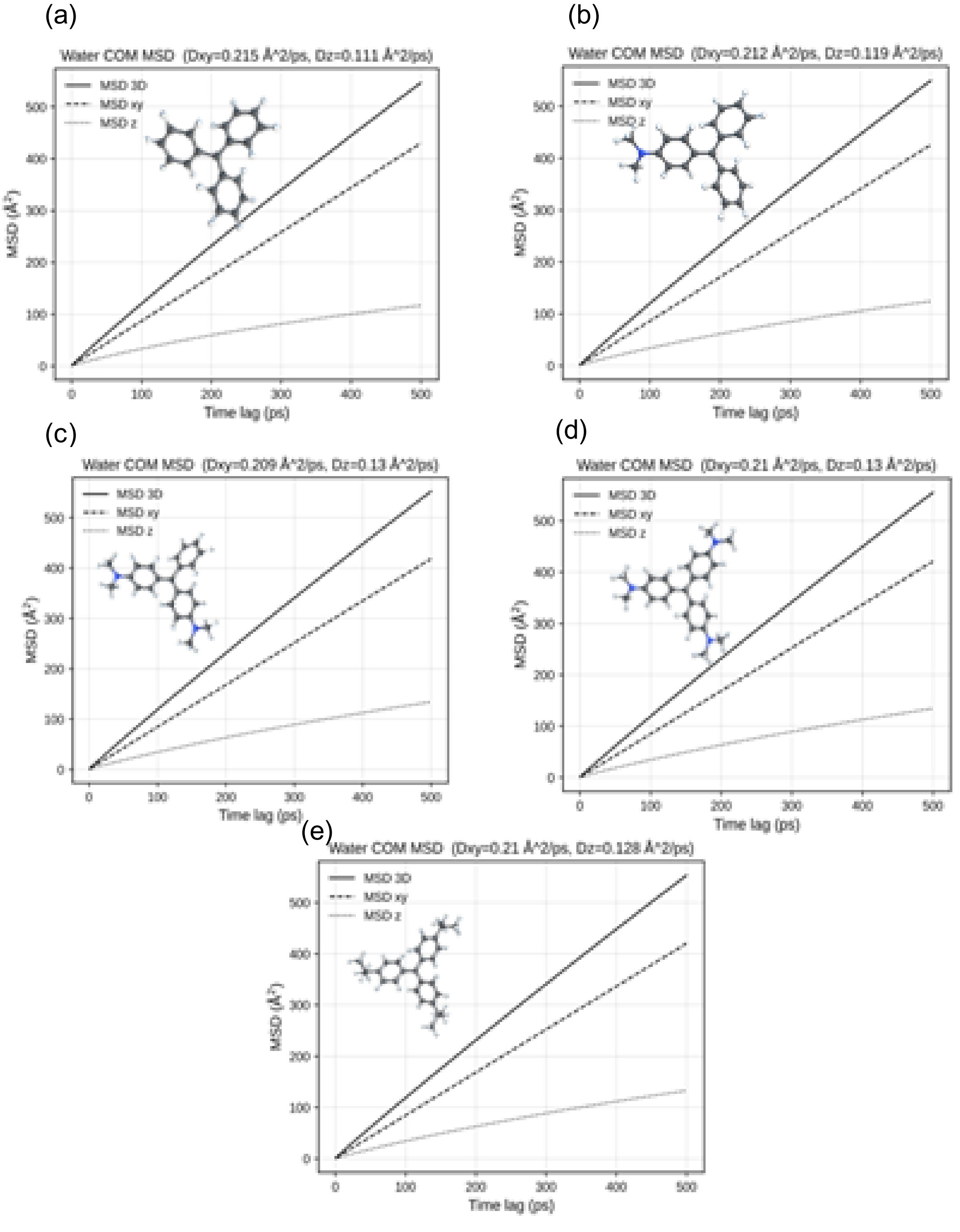

Figure S24. MSD curves over the diffusive time window computed from water COM trajectories of the dyes (a) TAM0, (b) TAM1, (c) TAM2, (d) TAM3, and (e) TAM3I from single-slab bulk MD simulations at 300 K using the SPC-FW

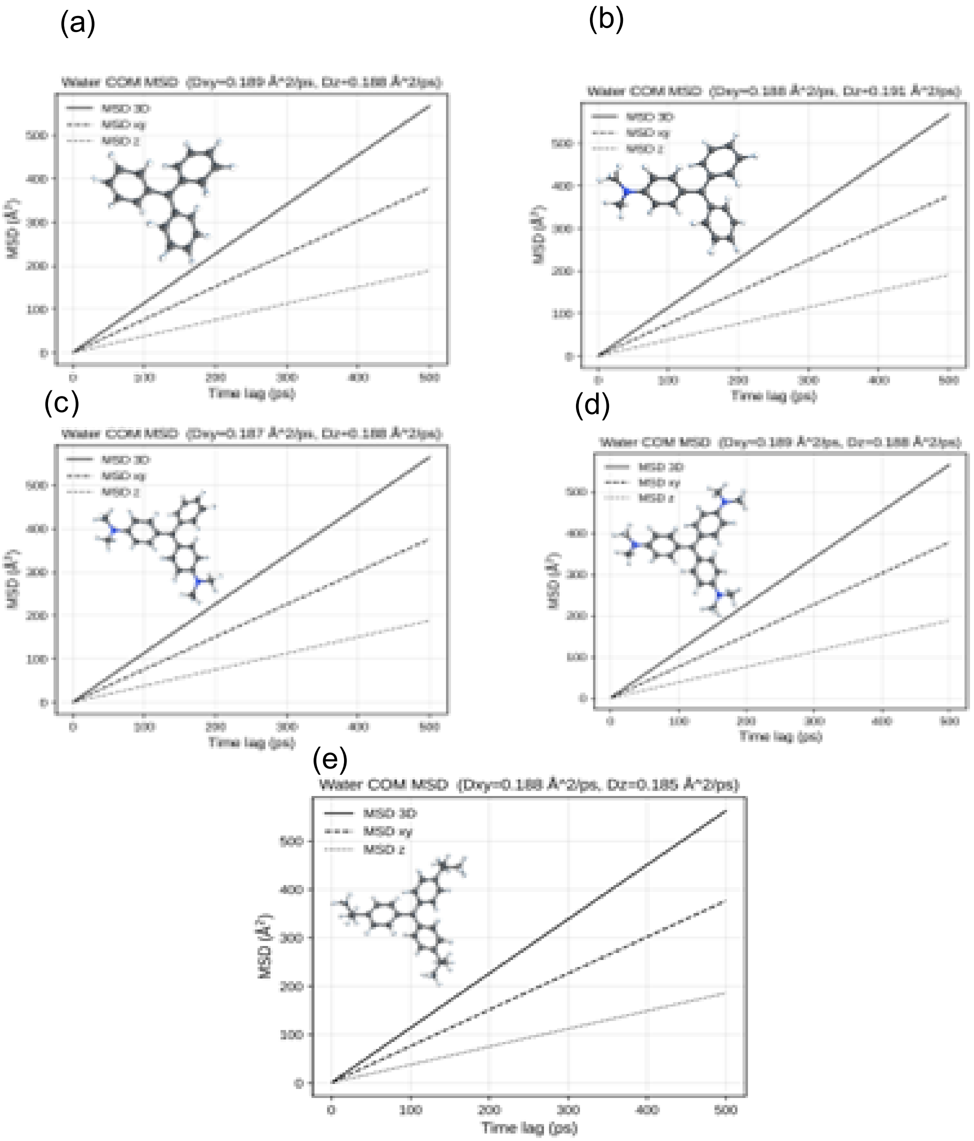

Figure S25. MSD curves over the diffusive time window computed from water COM trajectories of the dyes (a) TAM0, (b) TAM1, (c) TAM2, (d) TAM3, and (e) TAM3I from single-slab bulk MD simulations at 300 K using the SPCFW-FW

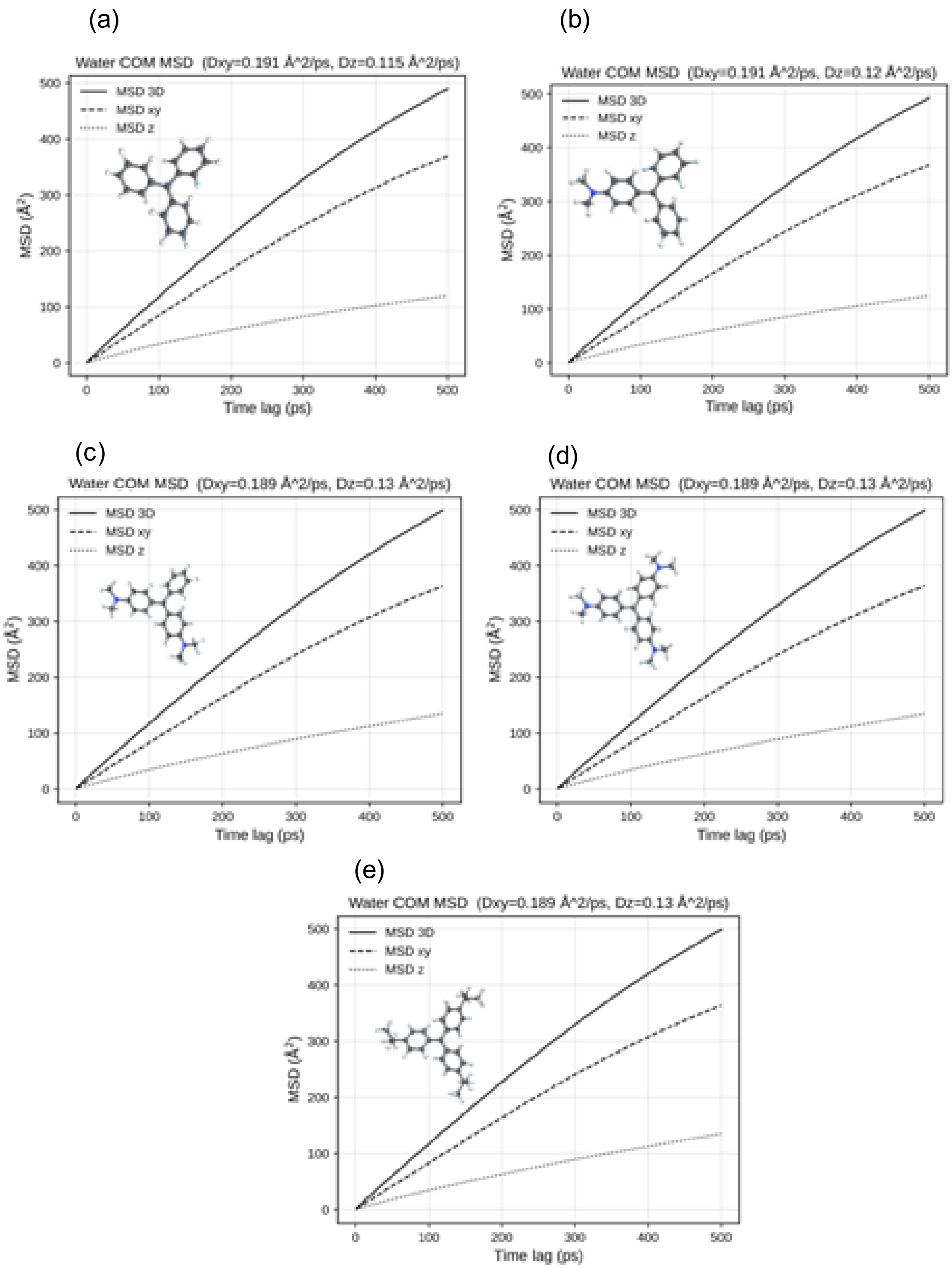

Figure S26. MSD curves over the diffusive time window computed from water COM trajectories of the dyes (a) TAM0, (b) TAM1, (c) TAM2, (d) TAM3, and (e) TAM3I from single-slab slab MD simulations at 300 K using the OPC3

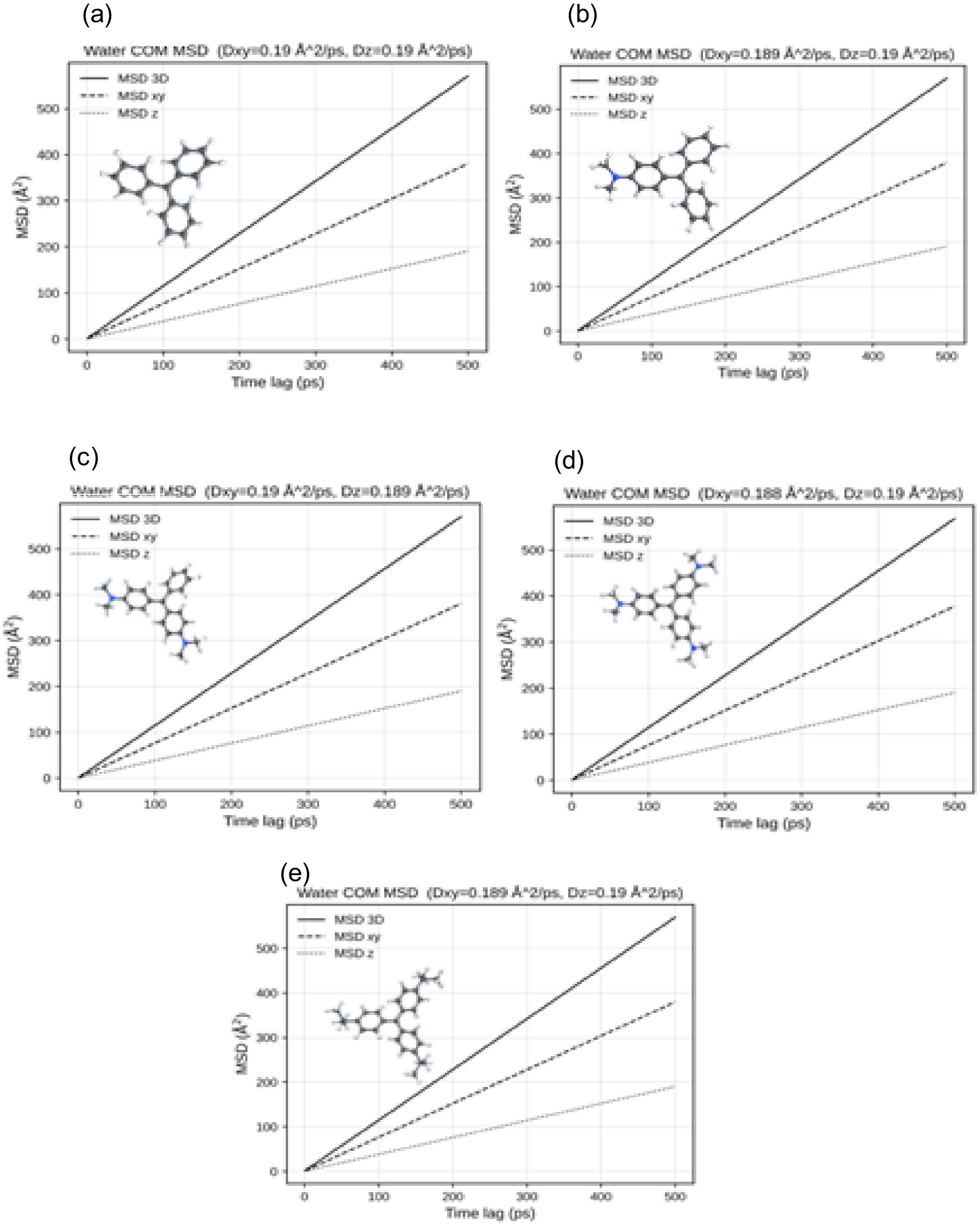

Figure S27. MSD curves over the diffusive time window computed from water COM trajectories of the dyes (a) TAM0, (b) TAM1, (c) TAM2, (d) TAM3, and (e) TAM3I from single-dye bulk MD simulations at 300 K using the OPC3

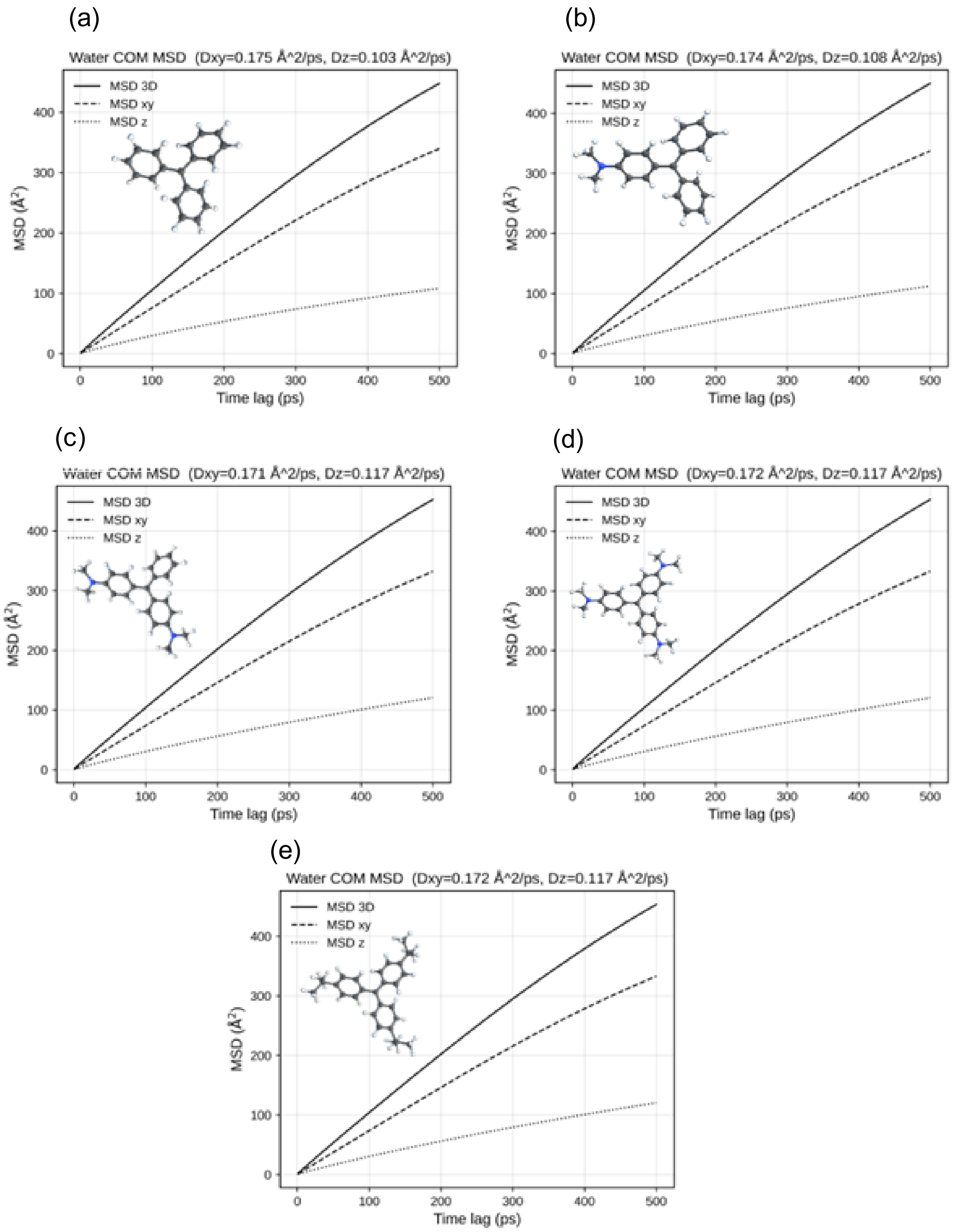

Figure S28. MSD curves over the diffusive time window computed from water COM trajectories of the dyes (a) TAM0, (b) TAM1, (c) TAM2, (d) TAM3, and (e) TAM3I from single-dye slab MD simulations at 300 K using the OPC3POL

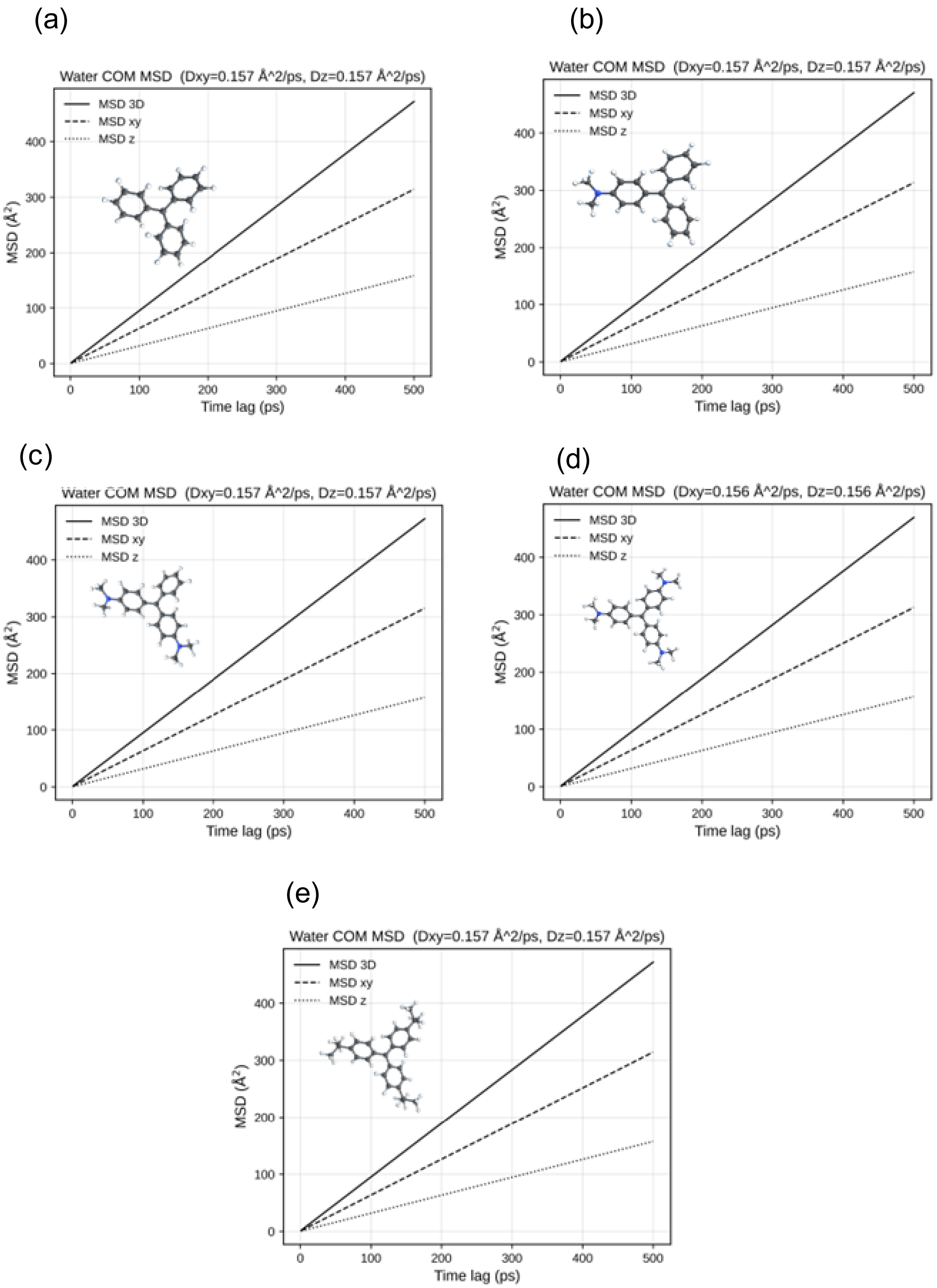

Figure S29. MSD curves over the diffusive time window computed from water COM trajectories of the dyes (a) TAM0, (b) TAM1, (c) TAM2, (d) TAM3, and (e) TAM3I from single-dye bulk MD simulations at 300 K using the OPC3POL

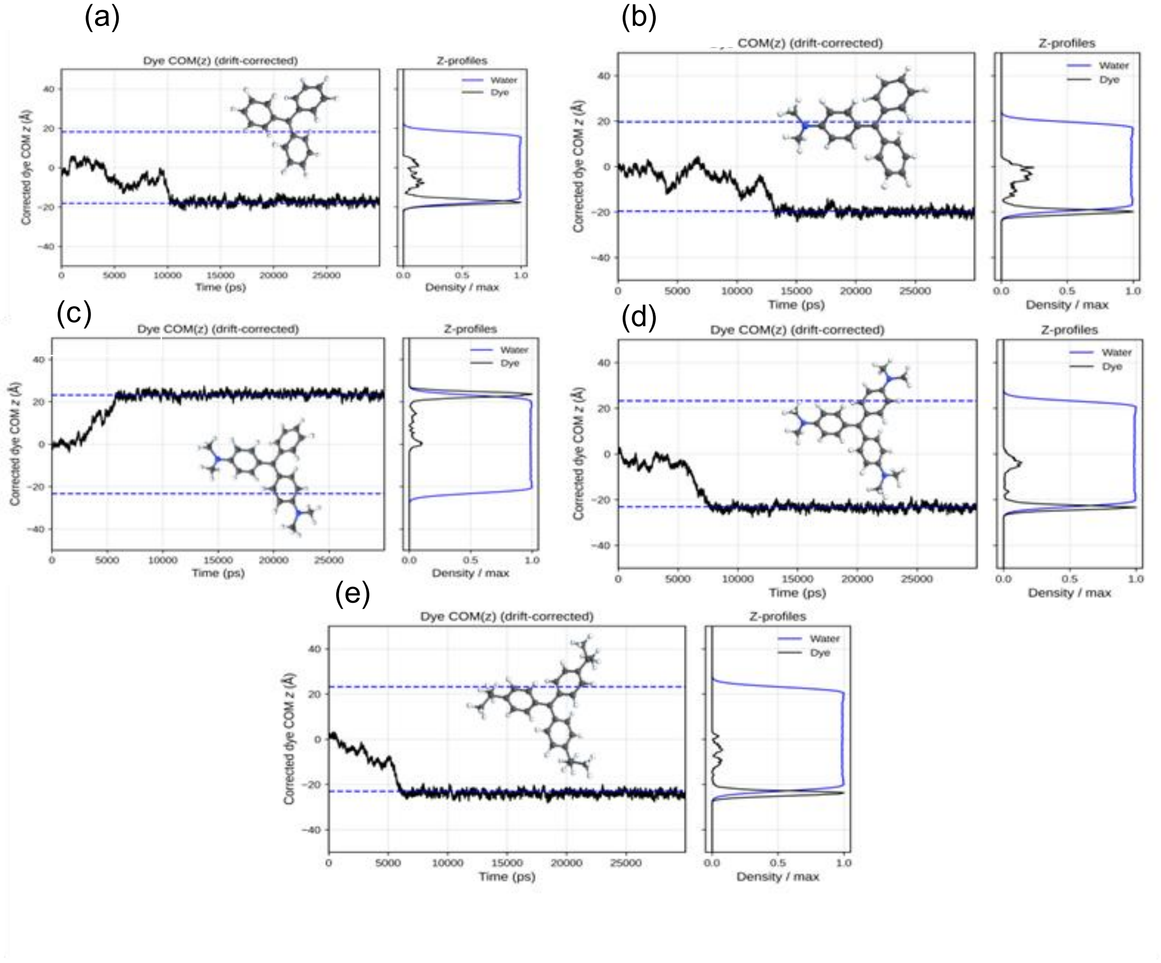

Figure S30. Drift-corrected COM position along the surface normal (z) as a function of time for the dyes (a) TAM0, (b) TAM1, (c) TAM2, (d) TAM3, and (e) TAM3I obtained from single-dyed slab MD simulations at 300 K using the OPC3-FW water model (Left panels). The drift-corrected coordinate is defined as $\Delta z = z\_COM(dye) - z\_COM\ (water)$ (Å), representing the dye position relative to the center of the water slab. Blue dashed lines indicate the positions of the air-water interfaces.

Normalized number-density profiles along the z direction for water (blue) and dye (black), illustrating the spatial distribution of each dye relative to the interfacial region (Right panels).

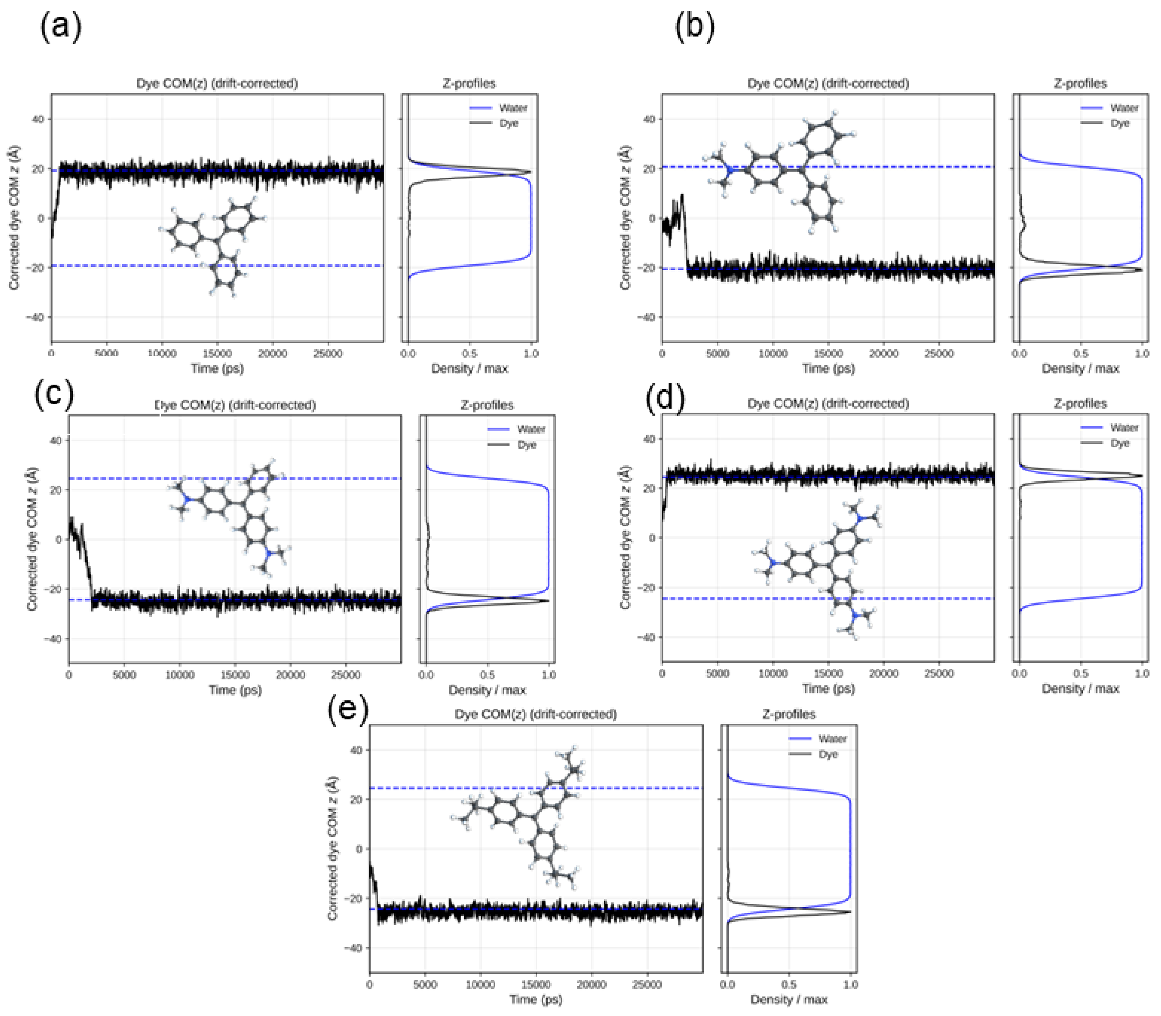

Figure S31. Drift-corrected COM position along the surface normal (z) as a function of time (Left panels) for the dyes (a) TAM0, (b) TAM1, (c) TAM2, (d) TAM3, and (e) TAM3I obtained from single-dyed slab MD simulations at 400 K using the OPC3-FW water model (Left panels). The drift-corrected coordinate is defined as Δz = z_COM(dye) − z_COM(water) (Å), representing the dye position relative to the center of the water slab. Blue dashed lines indicate the positions of the air-water interfaces.

Normalized number-density profiles along the z direction for water (blue) and dye (black), illustrating the spatial distribution of each dye relative to the interfacial region (Right panels)

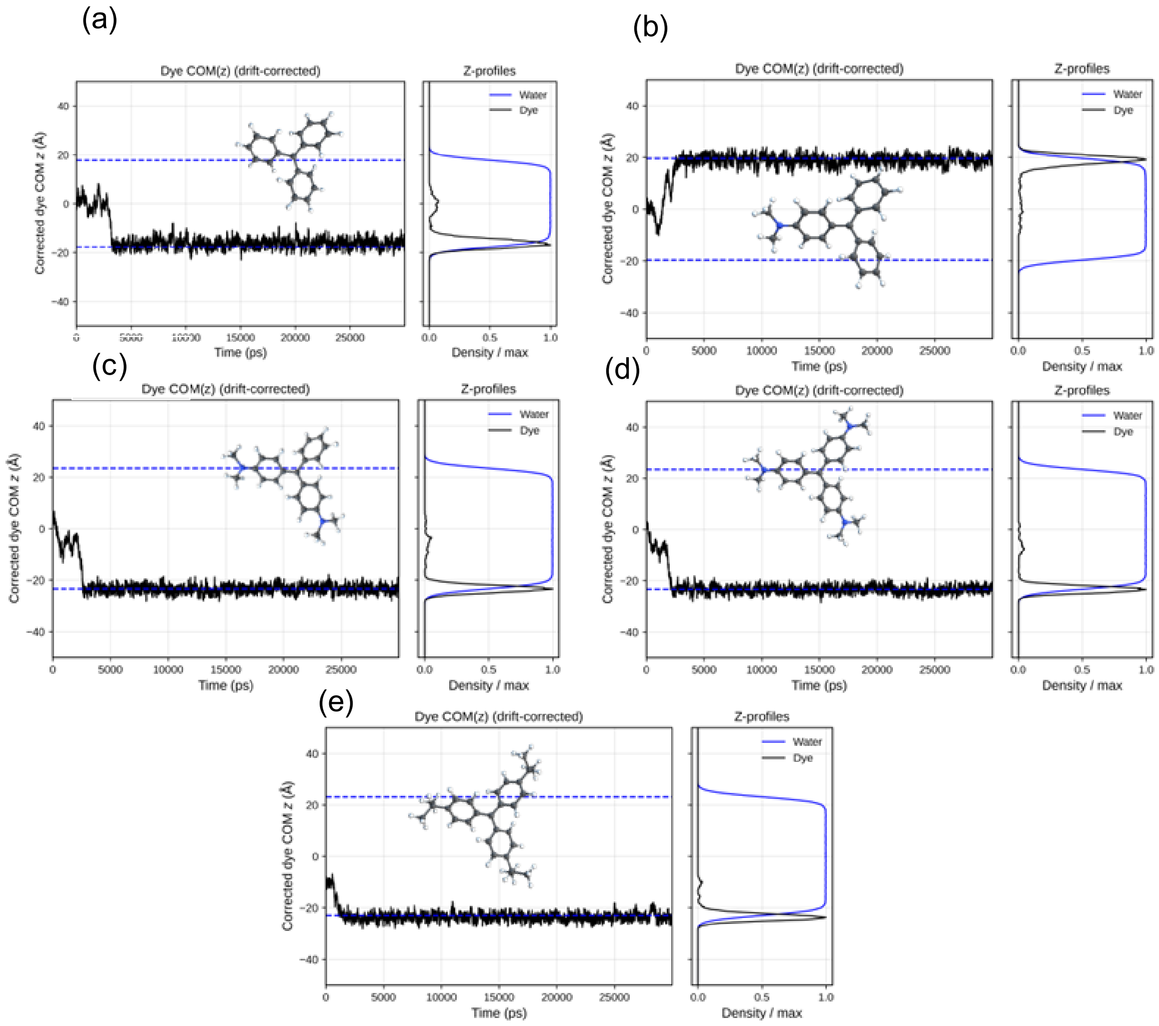

Figure S32. Drift-corrected COM position along the surface normal (z) as a function of time for the dyes (a) TAM0, (b) TAM1, (c) TAM2, (d) TAM3, and (e) TAM3I obtained from single-dyed slab MD simulations at 300 K using the TIP3P-FW water model (Left panels). The drift-corrected coordinate is defined as Δz = z_COM(dye) − z_COM(water) (Å), representing the dye position relative to the center of the water slab. Blue dashed lines indicate the positions of the air–water interfaces.

Normalized number-density profiles along the z direction for water (blue) and dye (black), illustrating the spatial distribution of each dye relative to the interfacial region (Right panels).

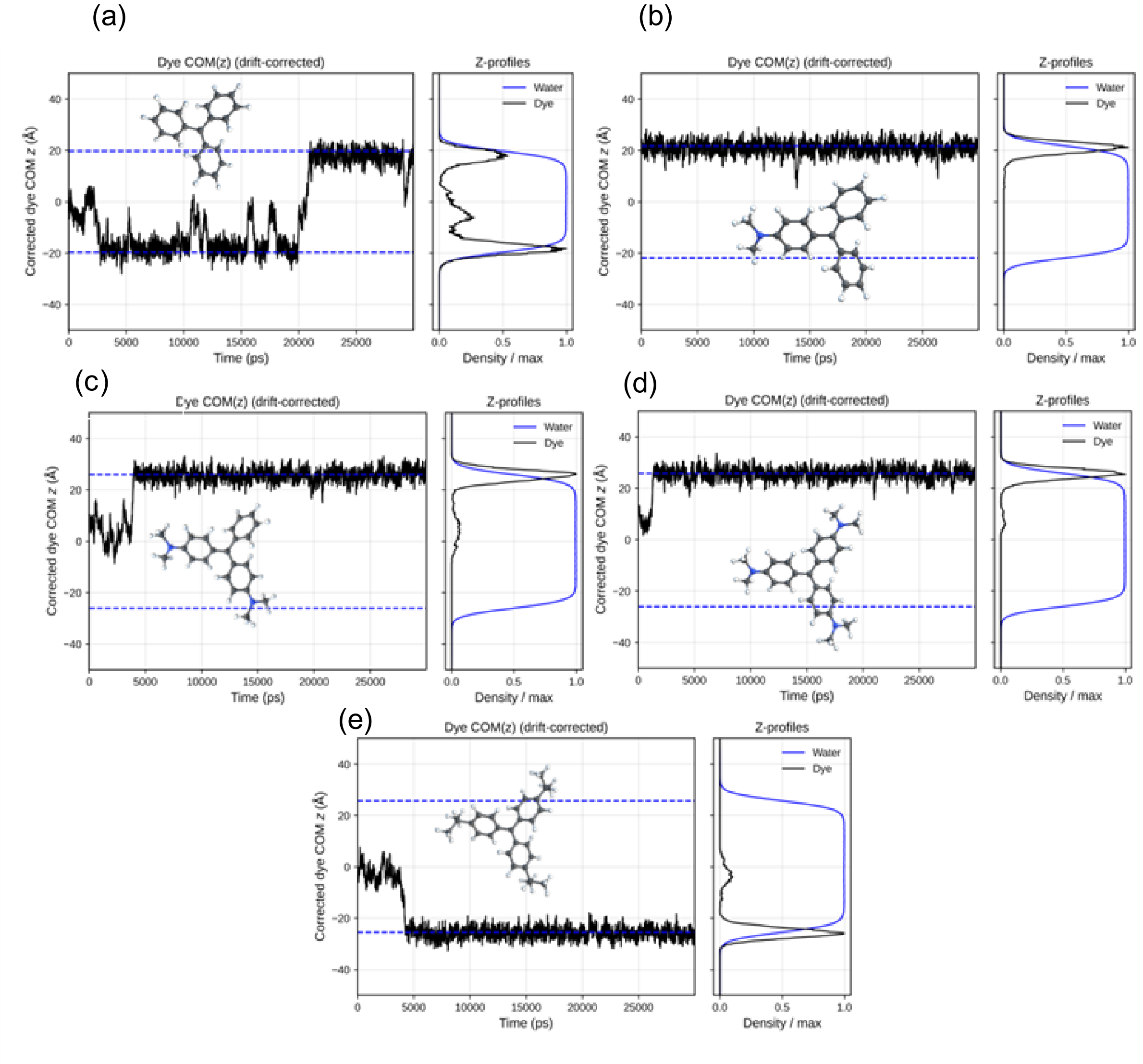

Figure S33. Drift-corrected COM position along the surface normal (z) as a function of time for the dyes (a) TAM0, (b) TAM1, (c) TAM2, (d) TAM3, and (e) TAM3I obtained from single-dyed slab MD simulations at 400 K using the TIP3P-FW water model (Left panels). The drift-corrected coordinate is defined as Δz = z_COM(dye) − z_COM(water) (Å), representing the dye position relative to the center of the water slab. Blue dashed lines indicate the positions of the air-water interfaces.

Normalized number-density profiles along the z direction for water (blue) and dye (black), illustrating the spatial distribution of each dye relative to the interfacial region (Right panels)

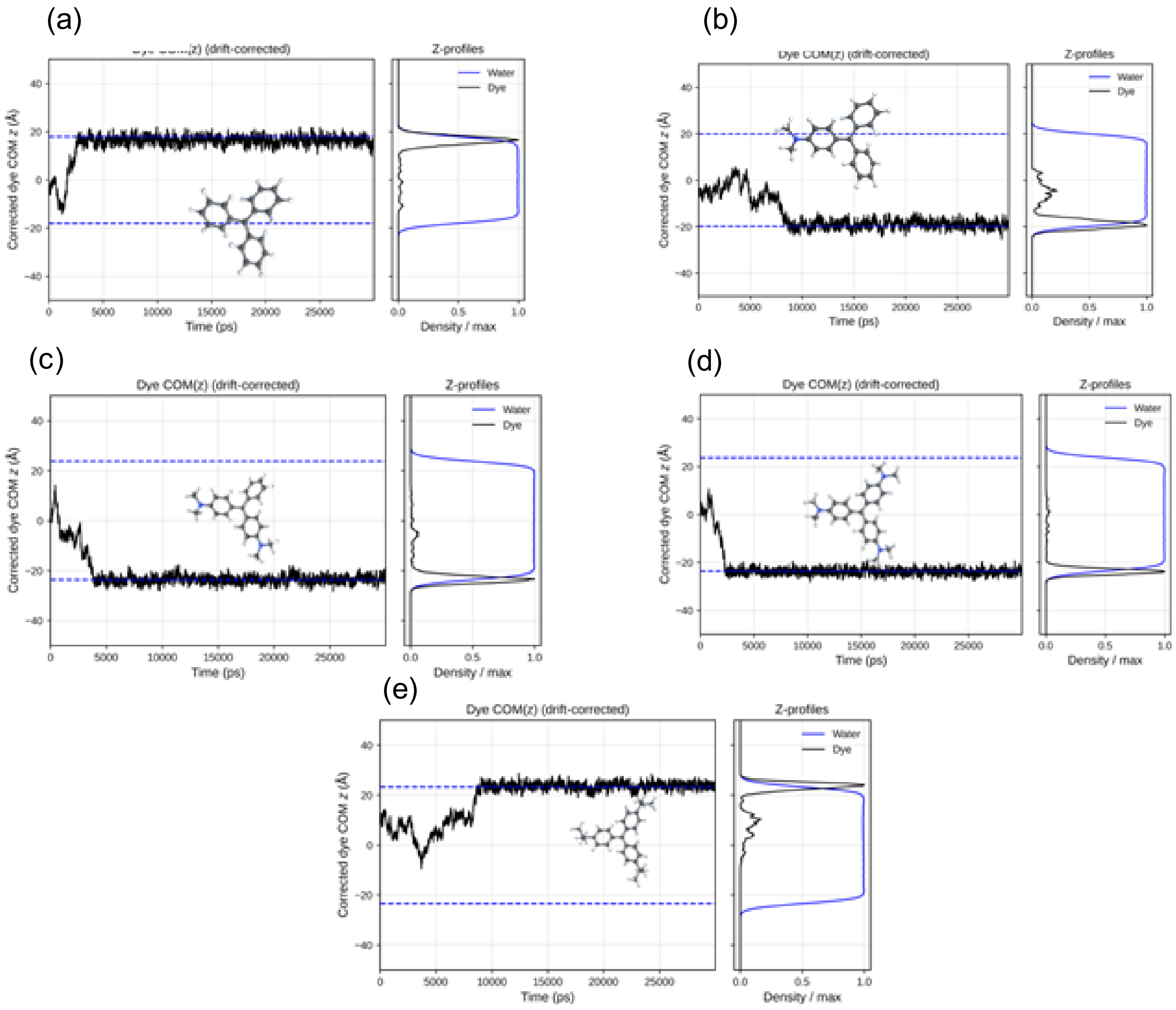

Figure S34. Drift-corrected COM position along the surface normal (z) as a function of time for the dyes (a) TAM0, (b) TAM1, (c) TAM2, (d) TAM3, and (e) TAM3I obtained from single-dyed slab MD simulations at 300 K using the SPC-FW water model (Left panels). The drift-corrected coordinate is defined as Δz = z_COM(dye) − z_COM (water) (Å), representing the dye position relative to the center of the water slab. Blue dashed lines indicate the positions of the air-water interfaces.

Normalized number-density profiles along the z direction for water (blue) and dye (black), illustrating the spatial distribution of each dye relative to the interfacial region (Right panels).

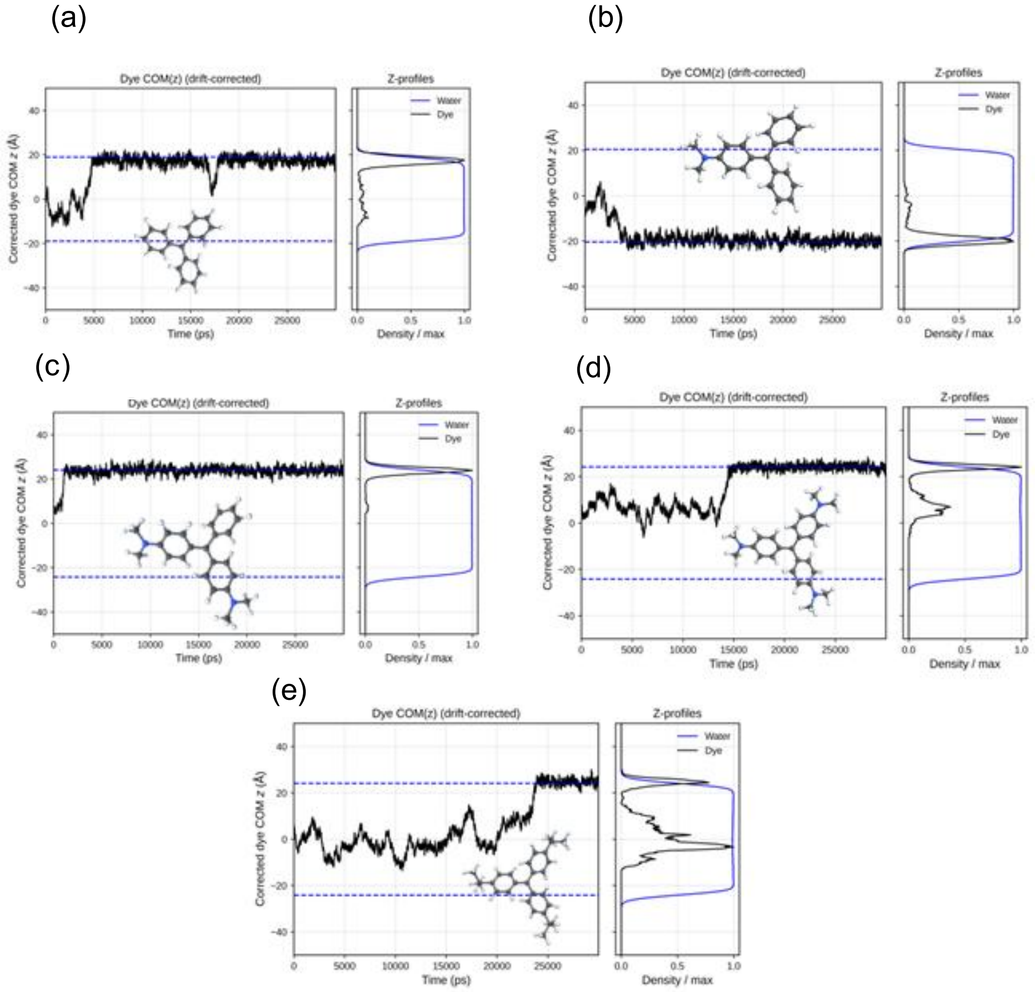

Figure S35. Drift-corrected COM position along the surface normal (z) as a function of time for the dyes (a) TAM0, (b) TAM1, (c) TAM2, (d) TAM3, and (e) TAM3I obtained from single-dyed slab MD simulations at 300 K using the OPC3 water model (Left panels). The drift-corrected coordinate is defined as Δz = z_COM(dye) − z_COM(water) (Å), representing the dye position relative to the center of the water slab. Blue dashed lines indicate the positions of the air-water interfaces.

Normalized number-density profiles along the z direction for water (blue) and dye (black), illustrating the spatial distribution of each dye relative to the interfacial region (Right panels)

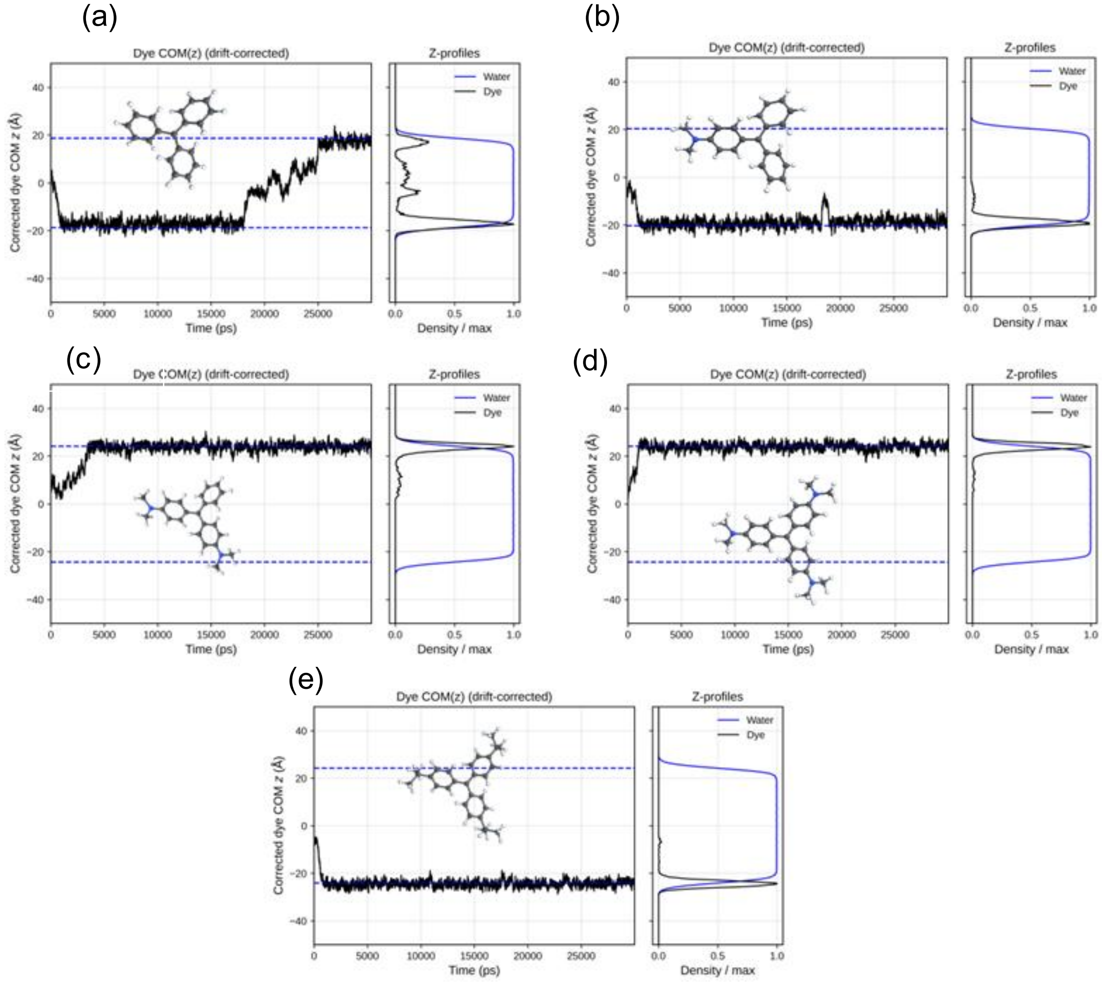

Figure S36. Drift-corrected COM position along the surface normal (z) as a function of time for the dyes (a) TAM0, (b) TAM1, (c) TAM2, (d) TAM3, and (e) TAM3I obtained from single-dyed slab MD simulations at 300 K using the OPC3-pol water model (Left panels). The drift-corrected coordinate is defined as Δz = z_COM(dye) − z_COM (water) (Å), representing the dye position relative to the center of the water slab. Blue dashed lines indicate the positions of the air-water interfaces.

Normalized number-density profiles along the z direction for water (blue) and dye (black), illustrating the spatial distribution of each dye relative to the interfacial region (Right panels).

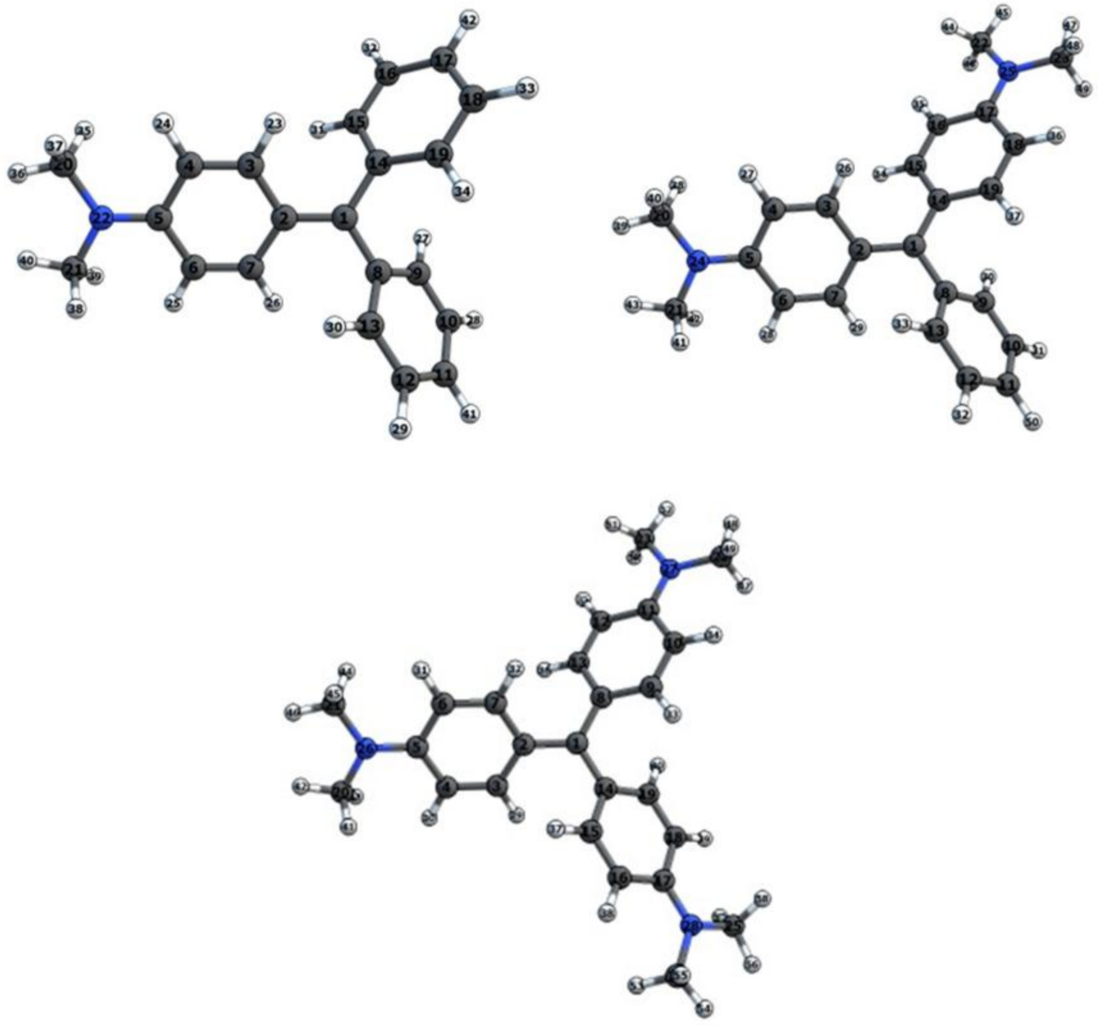

Figure S37. Atomic numbering scheme for the TAM1, TAM2, and TAM3 molecules used in dihedral analysis.

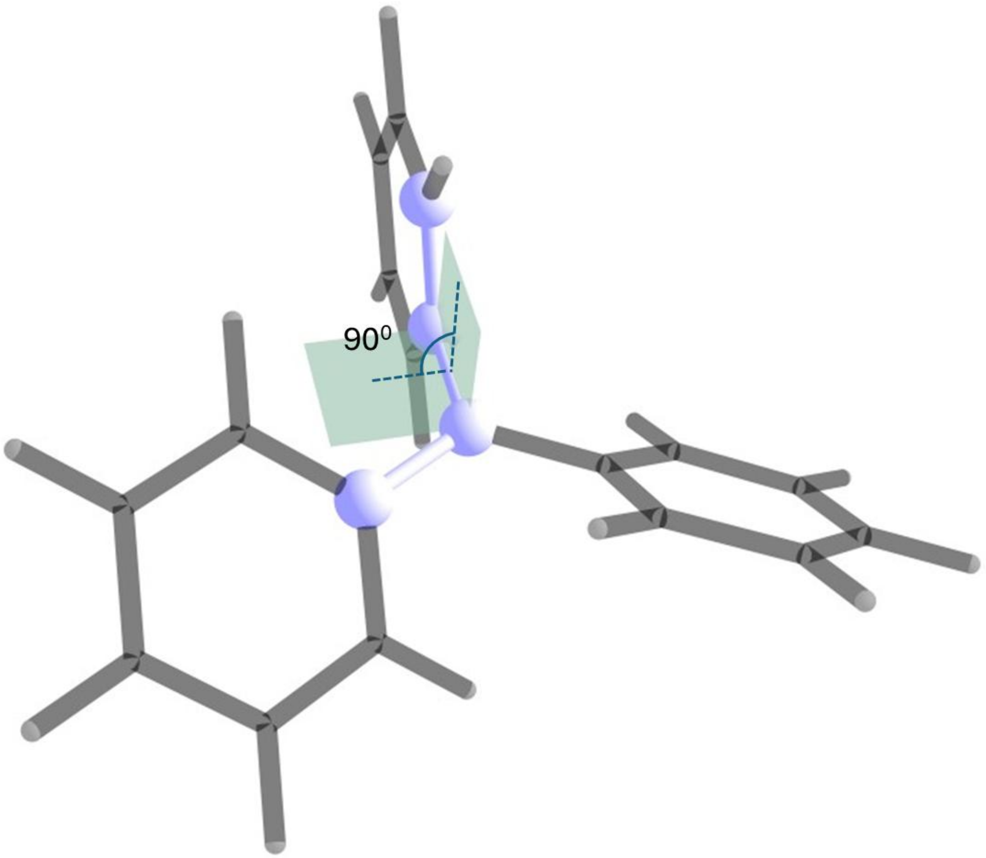

Figure S38. Dihedral angle of the non-substituted aryl ring

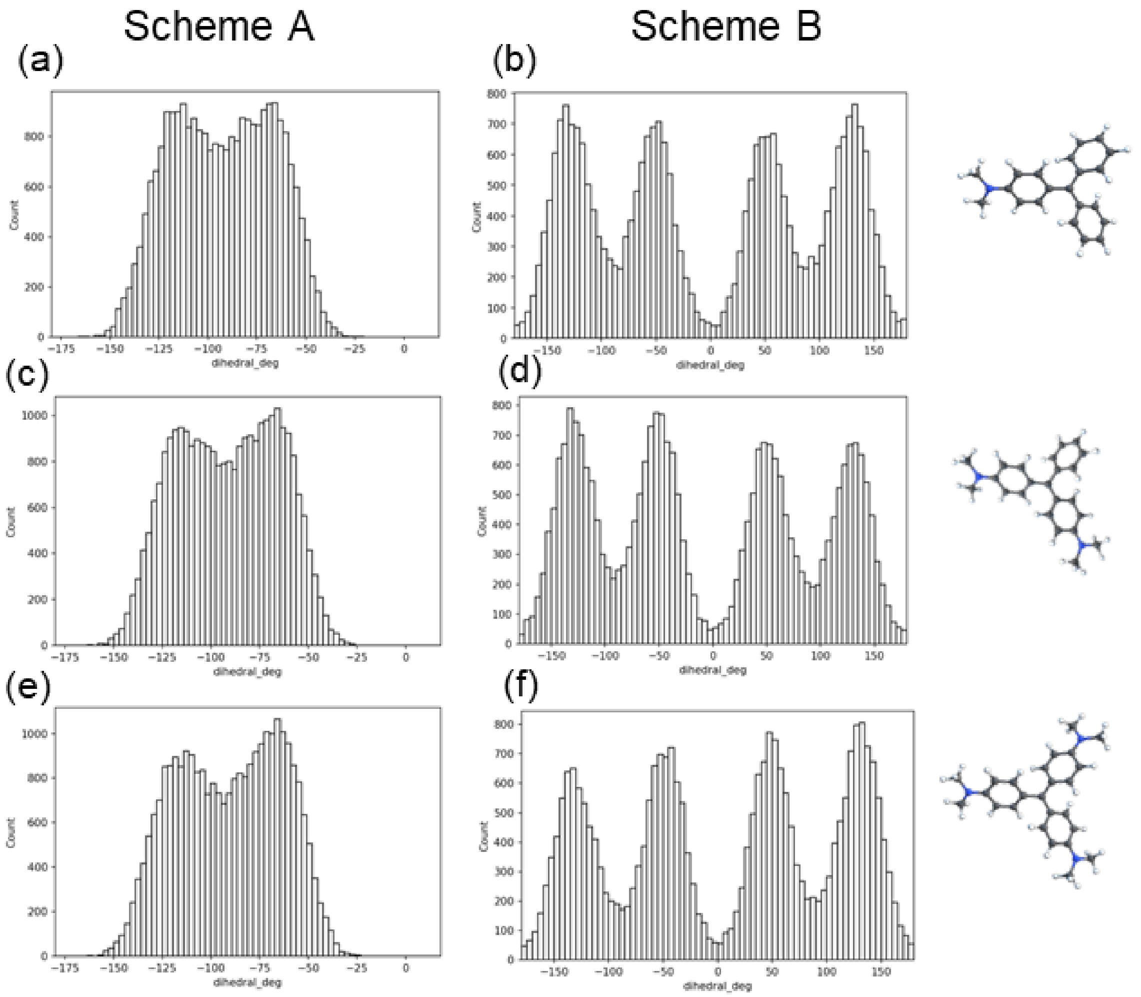

Figure S39. Comparison of dihedral angle13,8,1,2 distributions obtained with type A (left) and type B (right) parametrizations for the dyes (a,b) TAM1, (c,d) TAM2, (e,f) TAM3 obtained from single-dyed slab MD simulations at 400 K using the OPC3- FW water model.

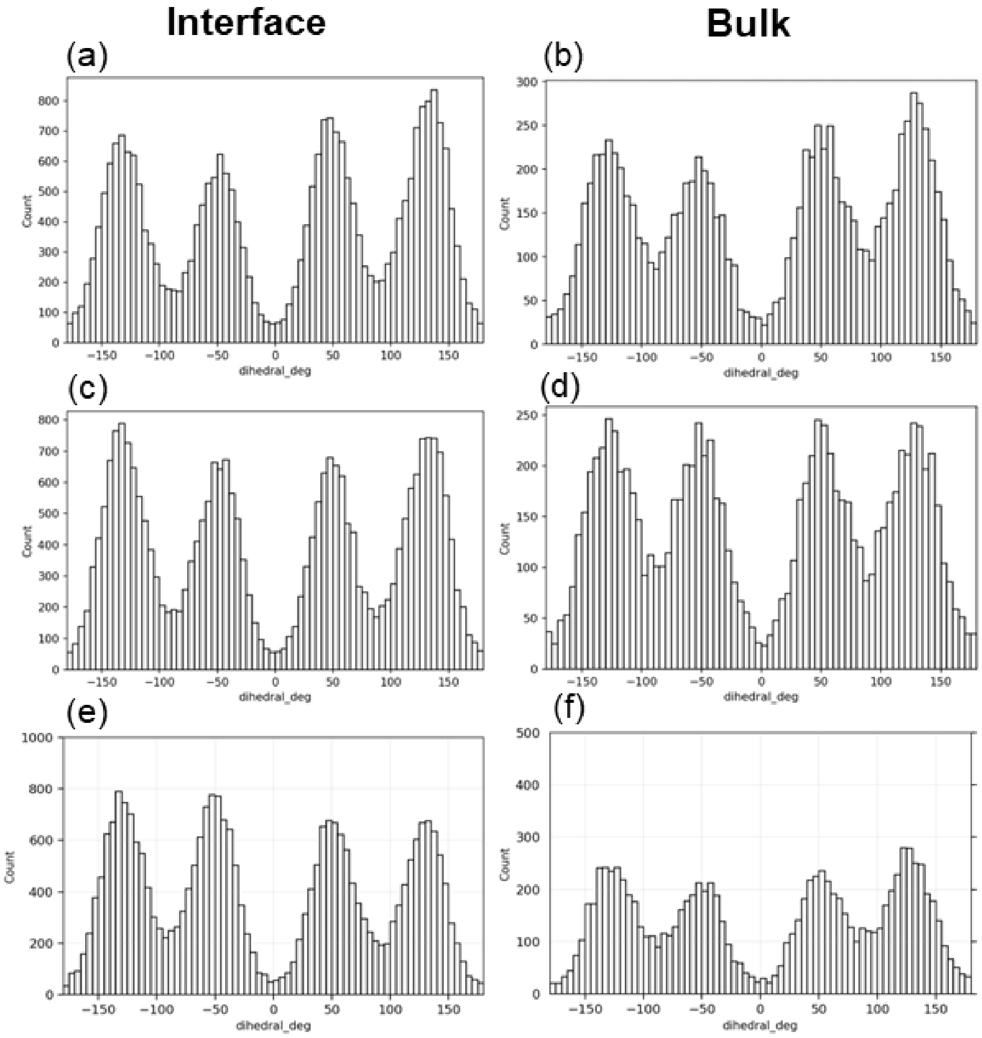

Figure S40. Probability distributions of dihedral 3,2,1,14 (a,b), dihedral 19,14,1,8 (c,d), and dihedral 13,8,1,2 (e,f) for TAM2 obtained from single-dye interface (left panels) and bulk (right panels) MD simulations using the type B parametrization OPC3-FW water model at 400 K.

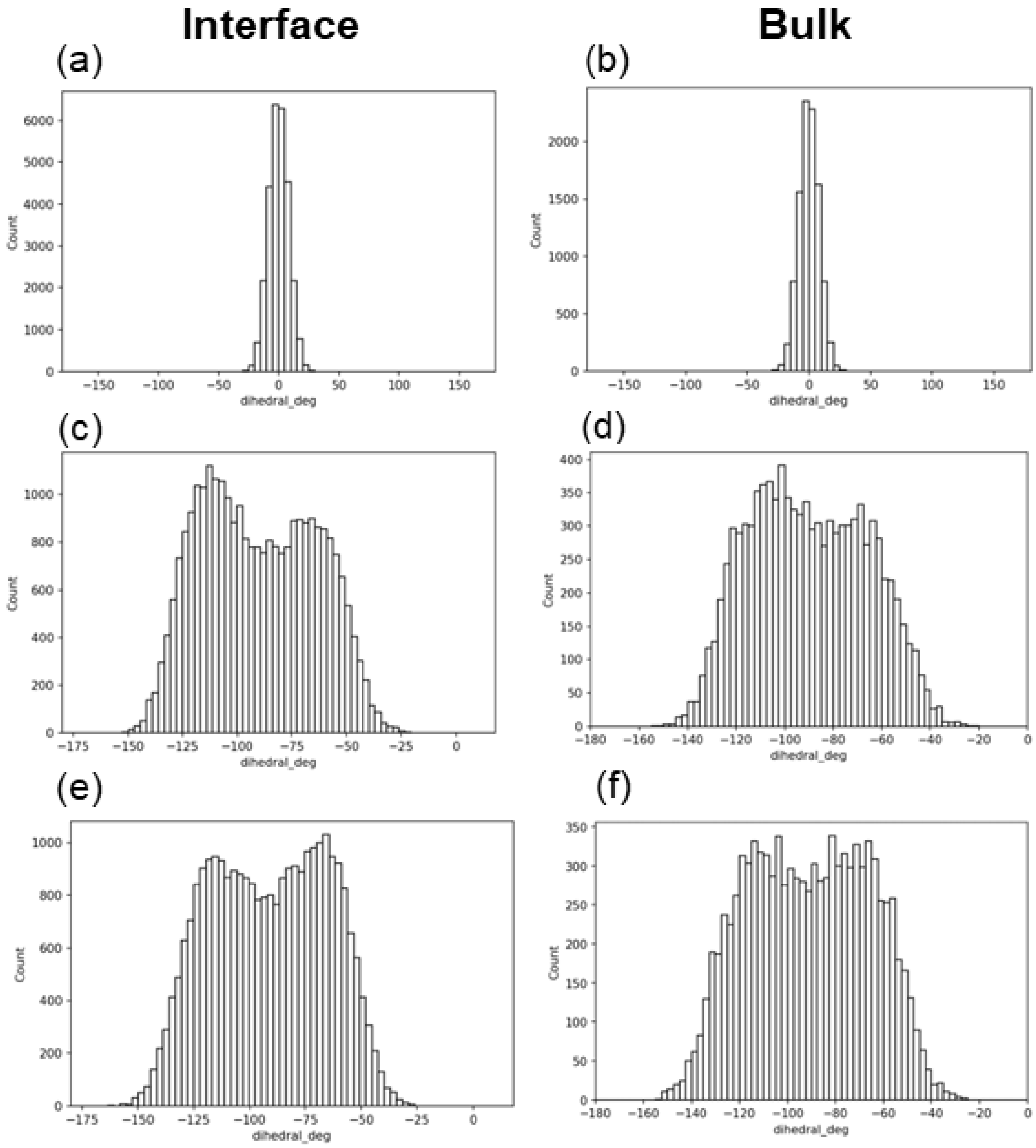

Figure S41. Probability distributions of dihedral 3,2,1,14 (a,b), dihedral 19,14,1,8 (c,d), and dihedral 13,8,1,2 (e,f) for TAM2 obtained from single-dye slab (left panels) and bulk (right panels) MD simulations using the type A parametrization OPC3-FW water model at 400 K.

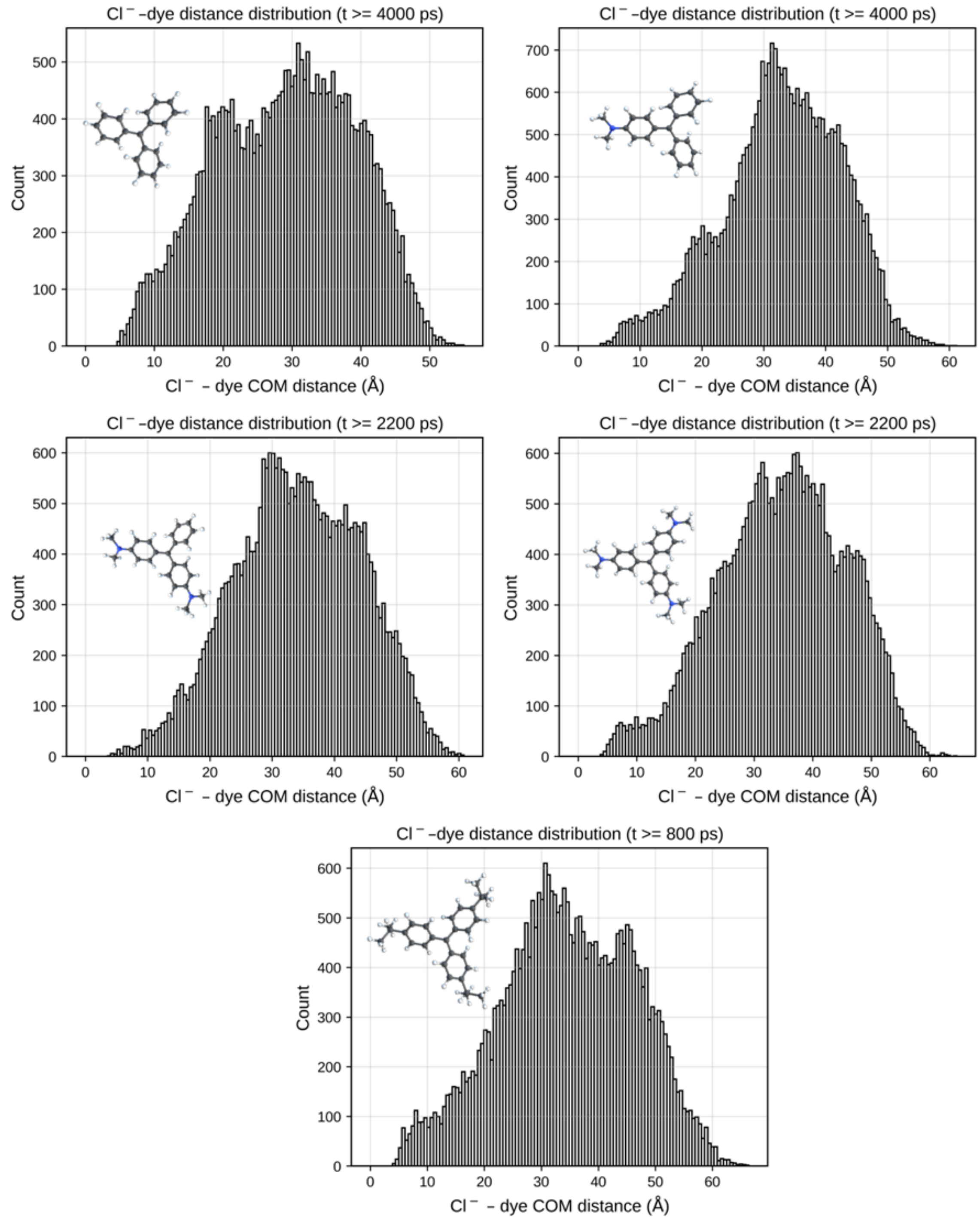

Figure S42. Probability distributions of distance between counterion and dye for (a) TAM0, (b) TAM1, (c) TAM2, (d) TAM3, and (e) TAM3I obtained from single-dye slab MD simulations using OPC3-FW at 400 K.

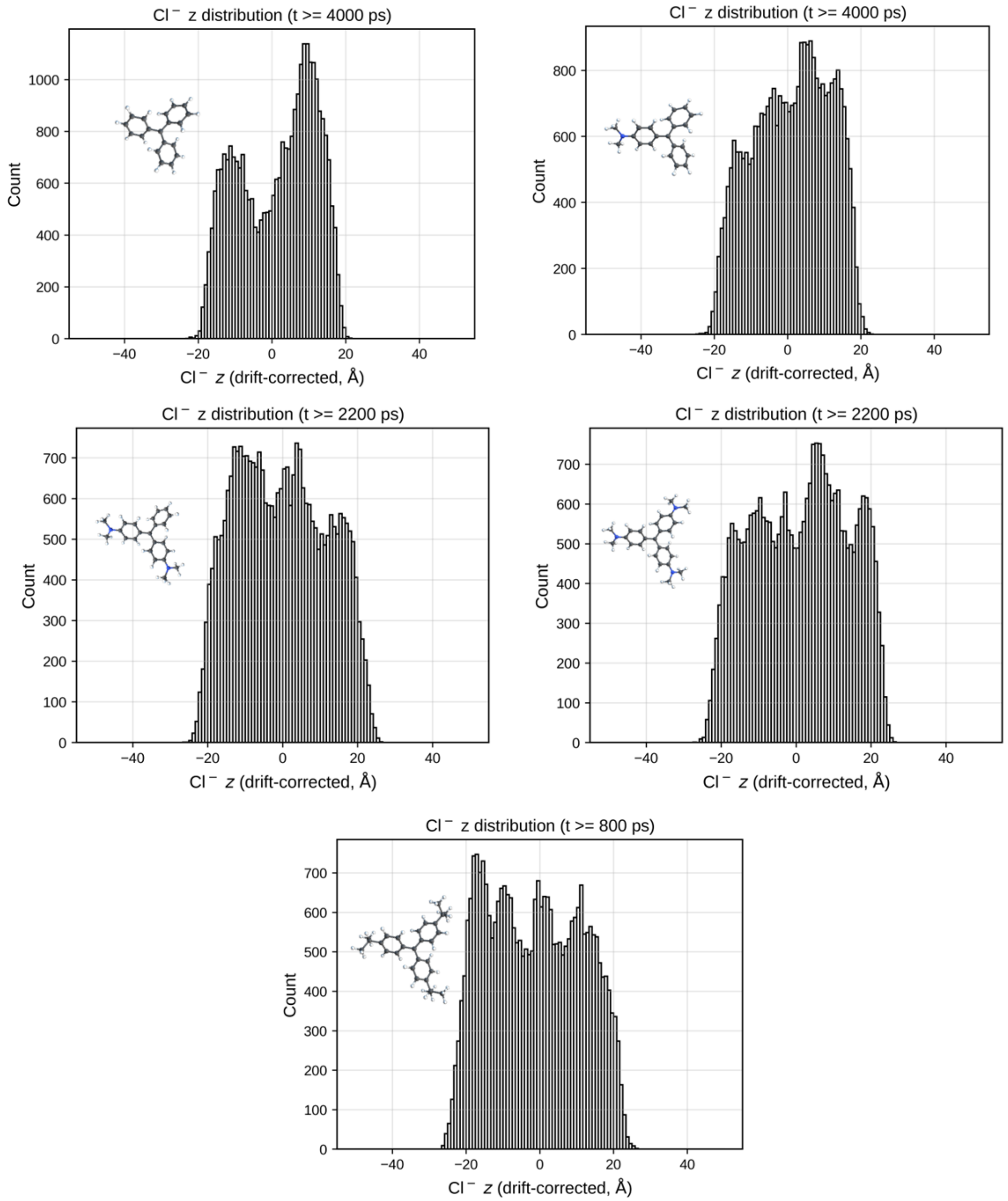

Figure S43. Counterion (Cl^-) probability distributions for (a) TAM0, (b) TAM1, (c) TAM2, (d) TAM3, and (e) TAM3I obtained from single-dye slab MD simulations using OPC3-FW at 400 K.

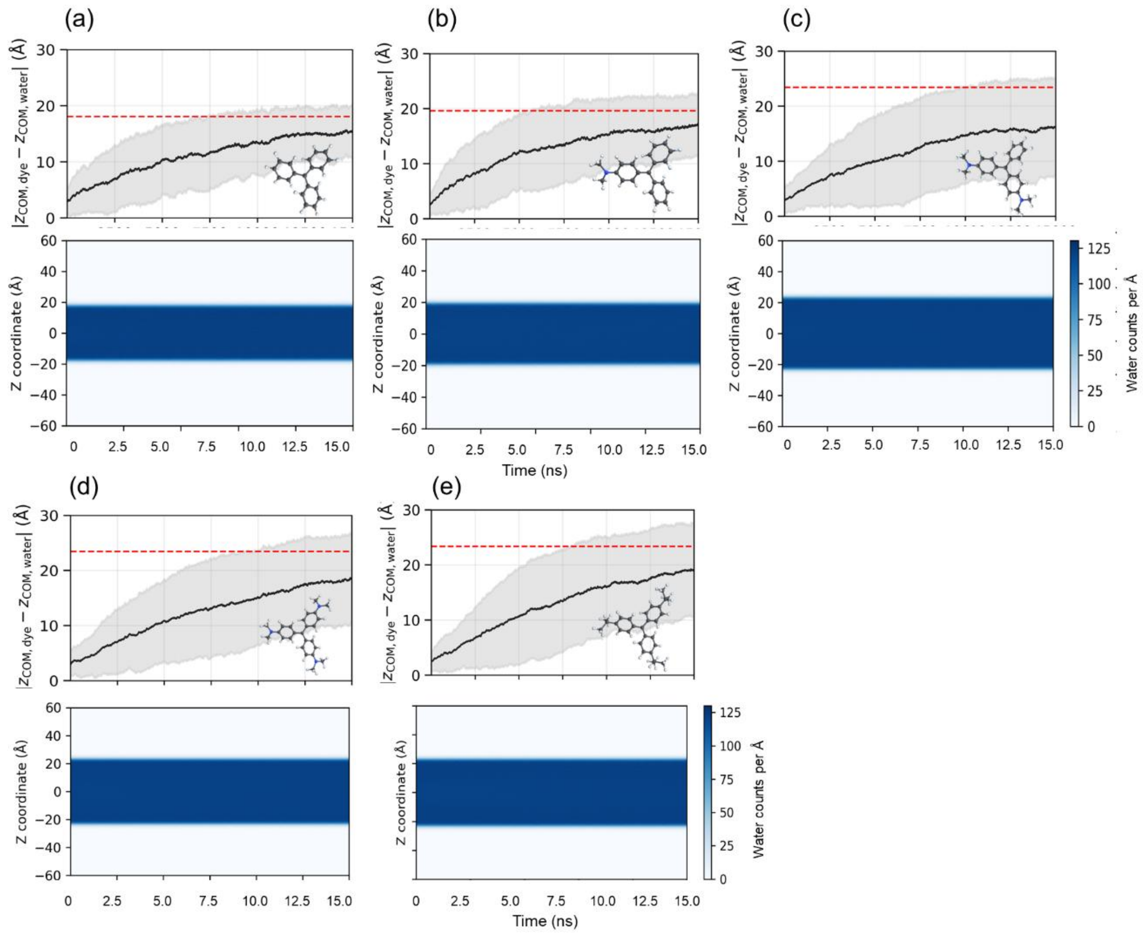

Figure S44. Drift-corrected distance between the dye and water slab centers of mass, $|z_{\mathrm{COM,dye}} - z_{\mathrm{COM,water}}|$(Å) with time, for (a) TAM0, (b) TAM1, (c) TAM2, (d) TAM3, and (e) TAM3I from single-dye slab MD simulations at 300 K using the OPC3-pol-FW/A water model. Solid black lines show the average with shaded regions indicating standard deviation. Red dashed lines mark the average positions of the air-water interfaces. Lower panels present the corresponding water number density maps along the z direction.

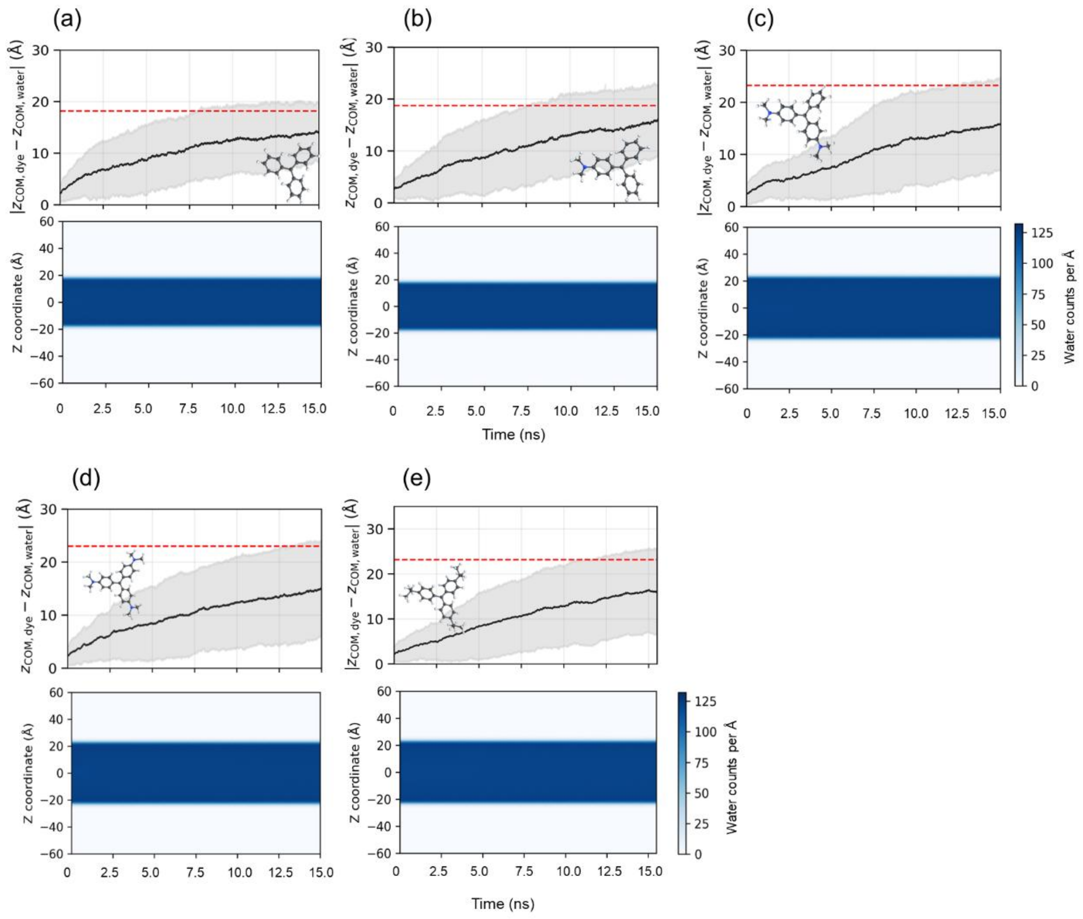

Figure S45. Drift-corrected distance between the dye and water slab centers of mass, $| z_{\mathrm{COM,dye}} - z_{\mathrm{COM,water}} |$(Å) with time, for (a) TAM0, (b) TAM1, (c) TAM2, (d) TAM3, and (e) TAM3I from single-dye slab MD simulations at 300 K using the OPC3-FW/A water model. Solid black lines show the average with shaded regions indicating standard deviation. Red dashed lines mark the average positions of the air-water interfaces. Lower panels present the corresponding time-resolved water number density maps along the z direction.

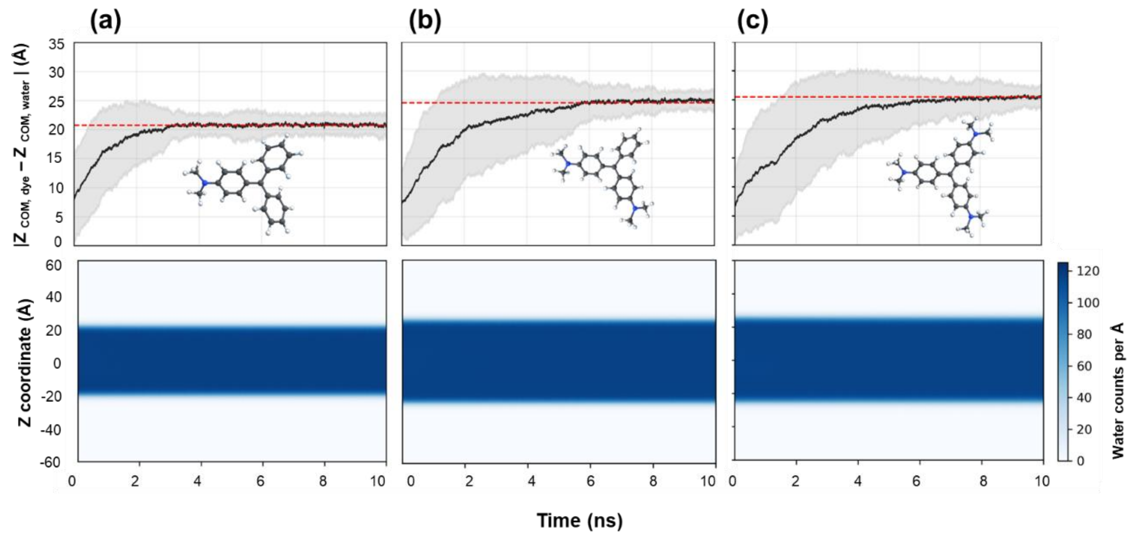

Figure S46. Drift-corrected distance between the dye and water slab centers of mass, $|\, z_{\mathrm{COM,dye}} - z_{\mathrm{COM,water}} \,|$(Å) with time, for (a) TAM1, (b) TAM2, and (c) TAM3 from single-dye slab MD simulations at 400 K using the OPC3-FW/A water model. Solid black lines show the average with shaded regions indicating standard deviation. Red dashed lines mark the average positions of the air-water interfaces. Lower panels present the corresponding time-resolved water number density maps along the z direction.

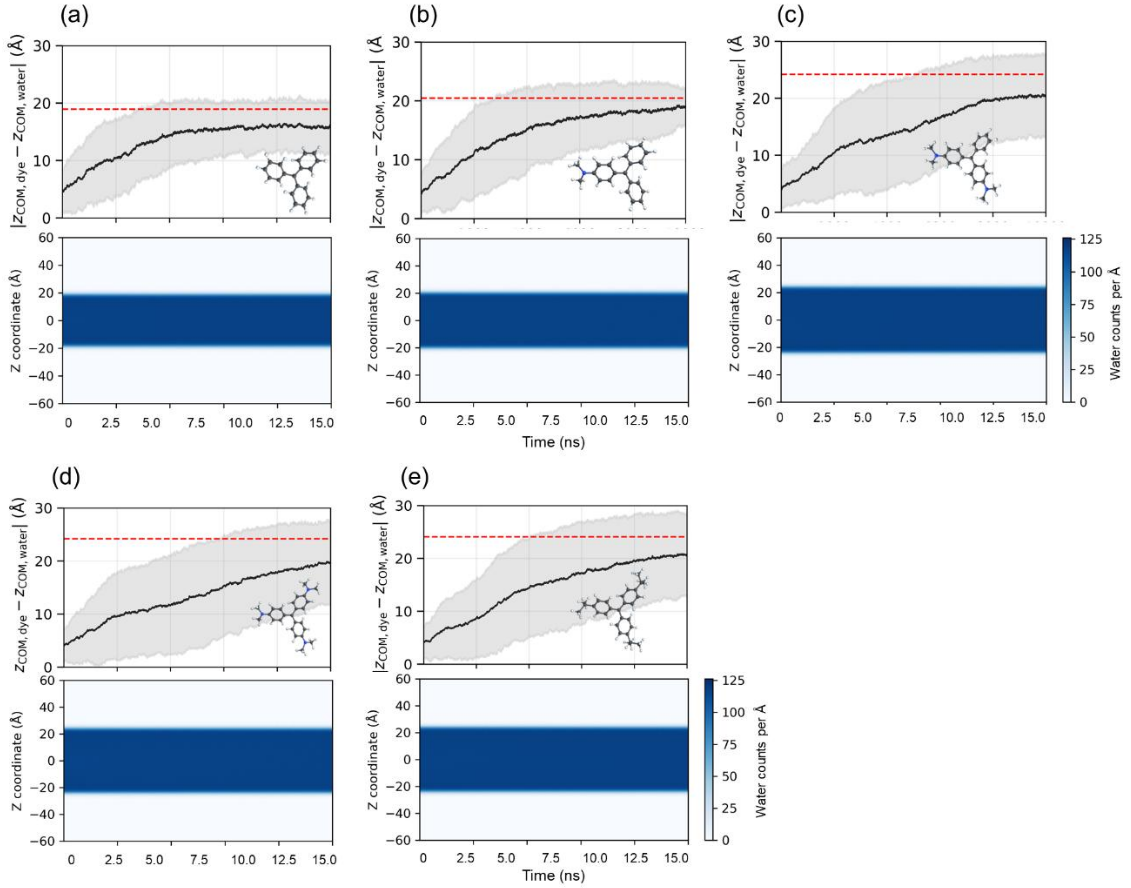

Figure S47. Drift-corrected distance between the dye and water slab centers of mass, $|z_{\mathrm{COM,dye}} - z_{\mathrm{COM,water}}|$(Å) with time, for (a) TAM0, (b) TAM1, (c) TAM2, (d) TAM3, and (e) TAM3I from single-dye slab MD simulations at 300 K using the OPC3/B water model. Solid black lines show the average with shaded regions indicating standard deviation. Red dashed lines mark the average positions of the air-water interfaces. Lower panels present the corresponding time-resolved water number density maps along the z direction.

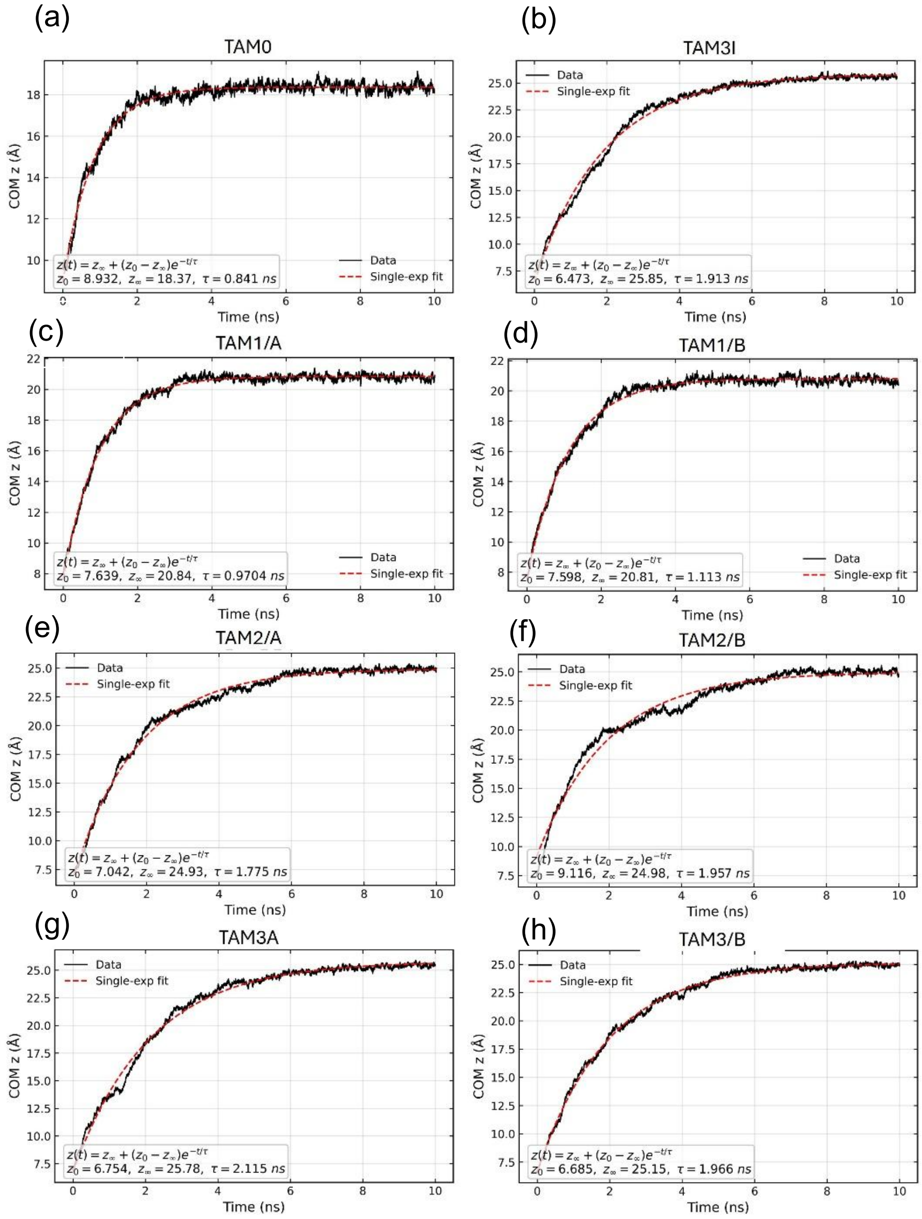

Figure S48. Dye COM position along time, for (a,b) TAM0, (c,d) TAM1, (e,f) TAM2, and (g,h) TAM3 from single-dye slab MD simulations at 400 K using the OPC3-FW water model with type A parameters (Left panels) and type B parameters (Right panels). Solid black lines show the simulation data. Red dashed lines represent the exponential fits.

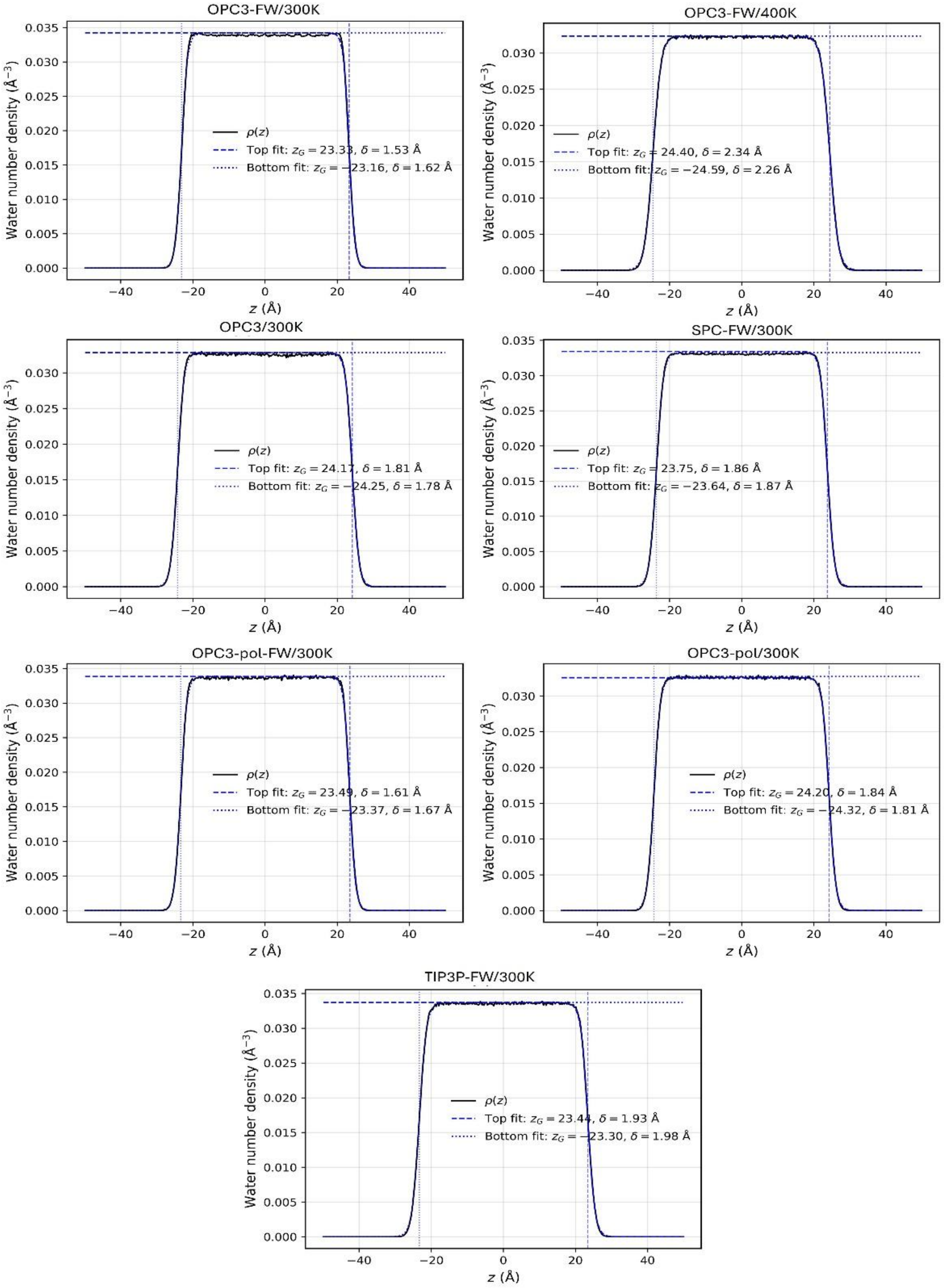

Figure S49. Water density profile along z axis, for different water models and temperatures from single-dye slab MD. Solid black lines show the simulation data. Blue dashed lines represent the fits.

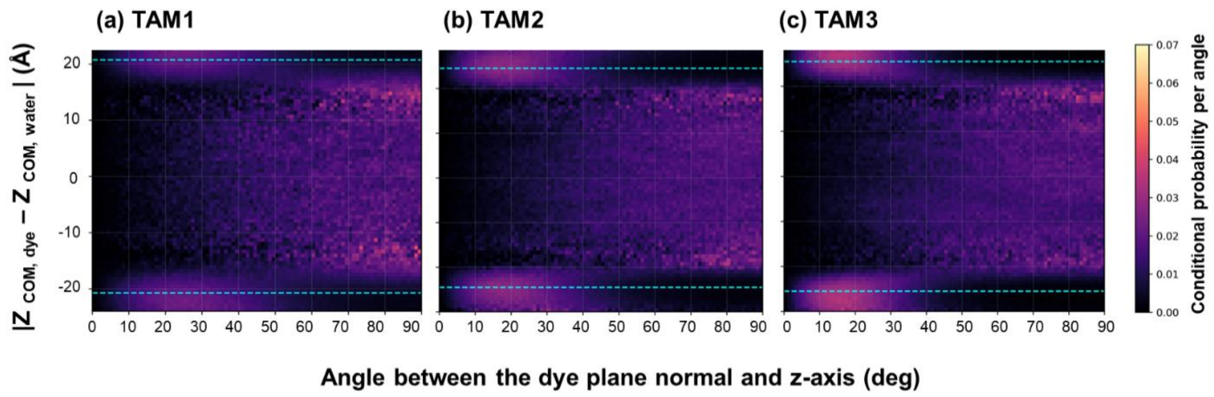

Figure S50. Number density distributions obtained from single-dyed slab MD simulations using the OPC3-FW/A water model at 400 K for (a) TAM1, (b) TAM2, and (c) TAM3.

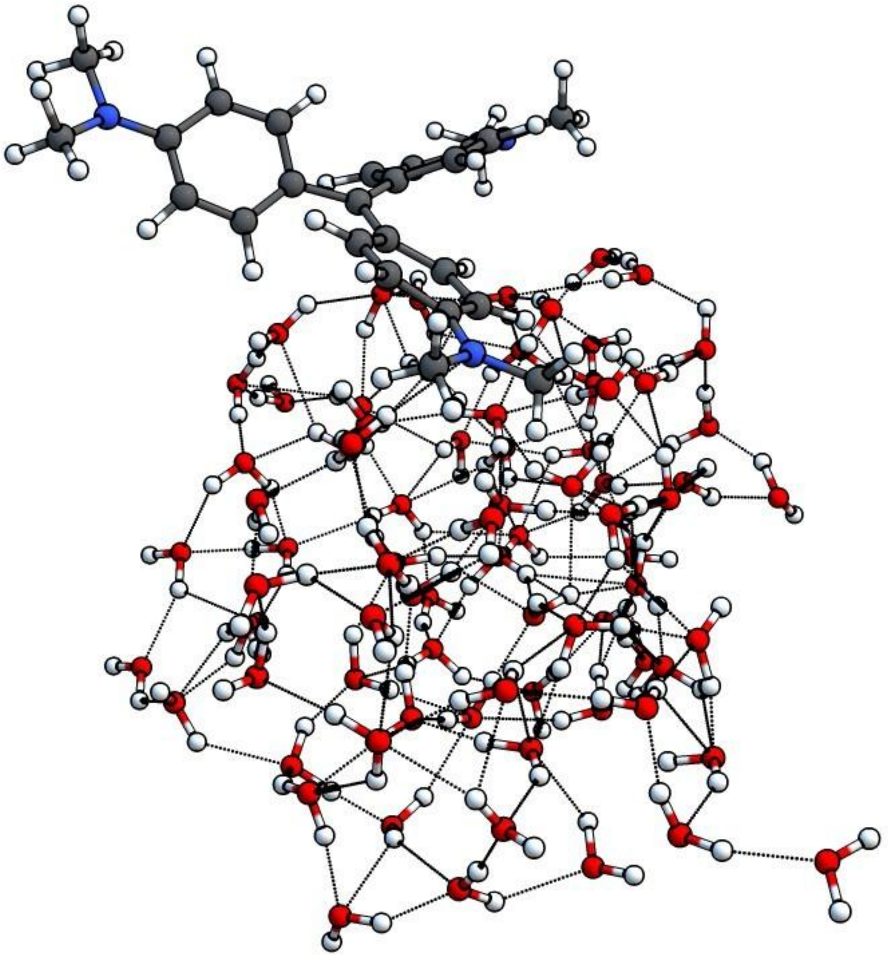

Figure S51. Representative snapshot at 20 ps from XTB simulations of TAM3 molecule solvated by 80 water molecules.

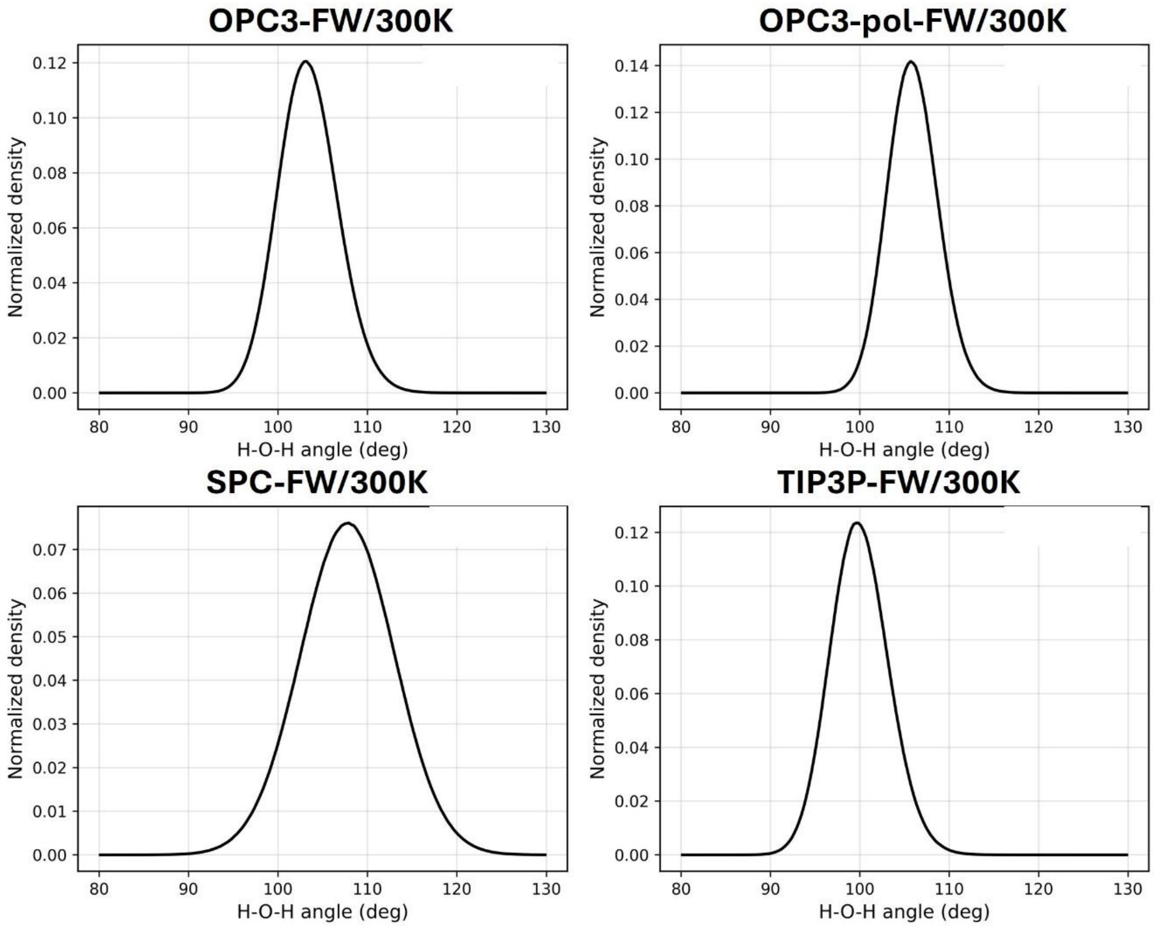

Figure S52. Normalized probability distributions of the H-O-H bond angle for bulk water at 300 K obtained from MD simulations using OPC3-FW, OPC3-pol-FW, SPC-FW, and TIP3P-FW water models.

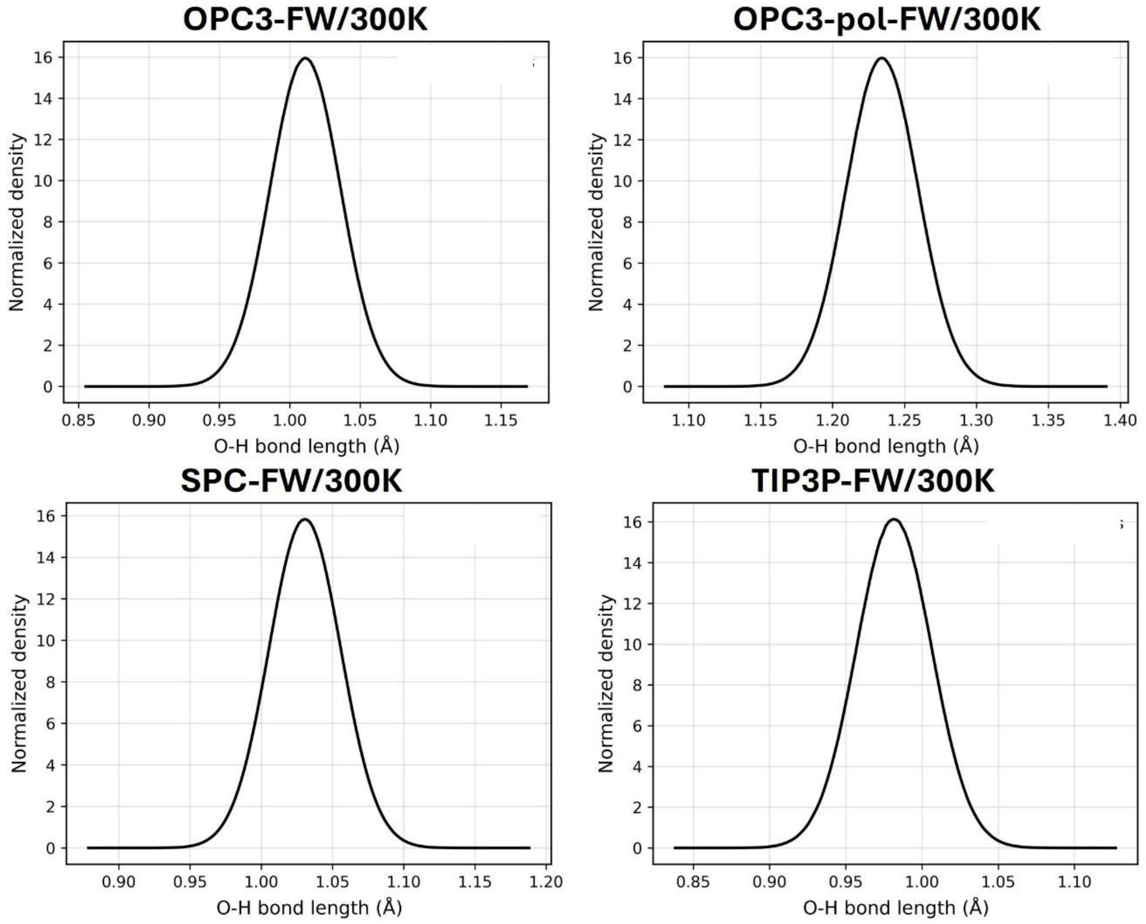

Figure S53. Normalized probability distributions of the O-H bond length for bulk water at 300 K obtained from MD simulations using OPC3-FW, OPC3-pol-FW, SPC-FW, and TIP3P-FW water models.

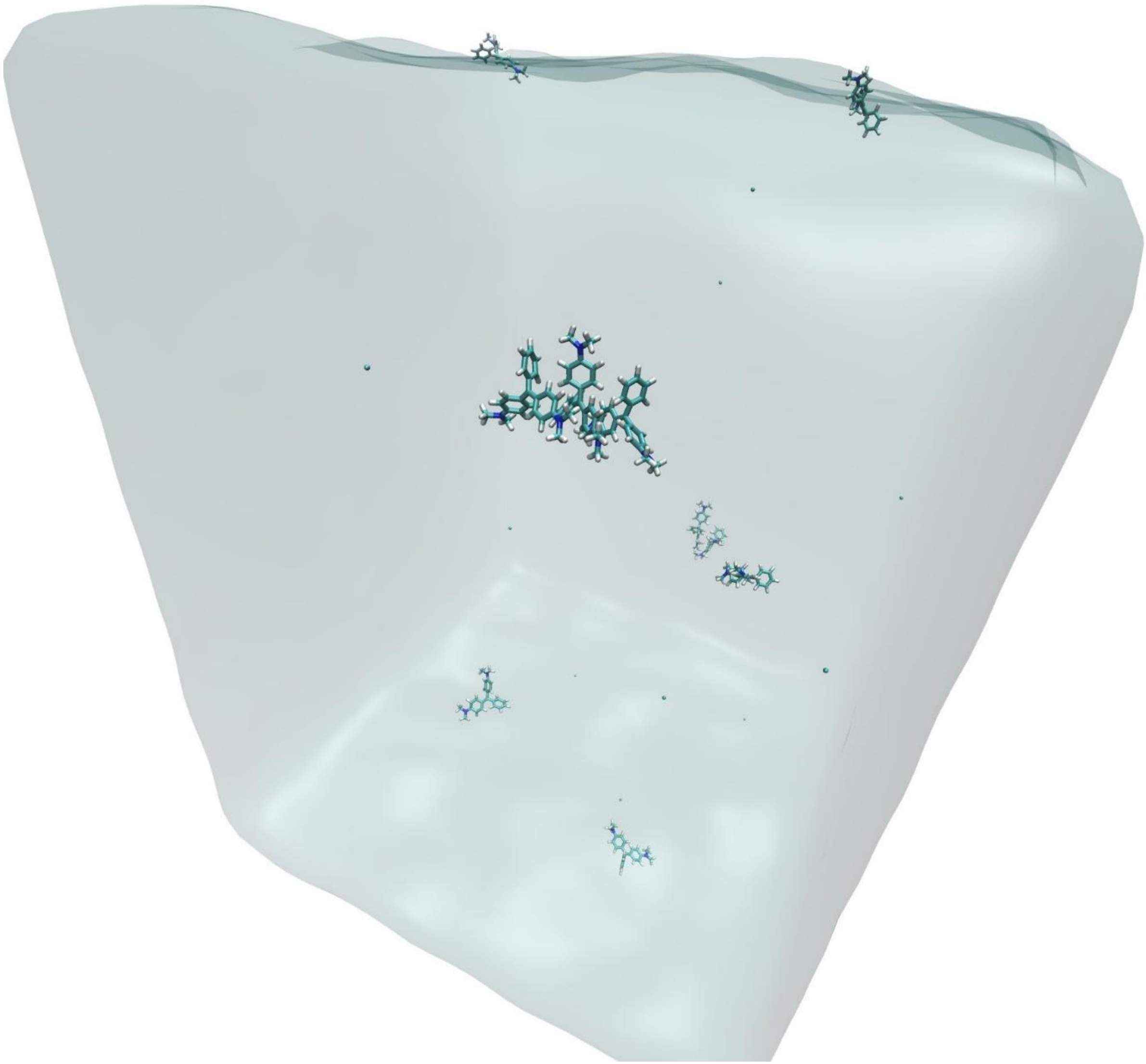

Figure S54. Representative snapshot (30 ns) from 10 TAM2 molecule MD simulation. The semi-transparent surface represents the water slab, and TAM2 molecules are shown in stick representation.

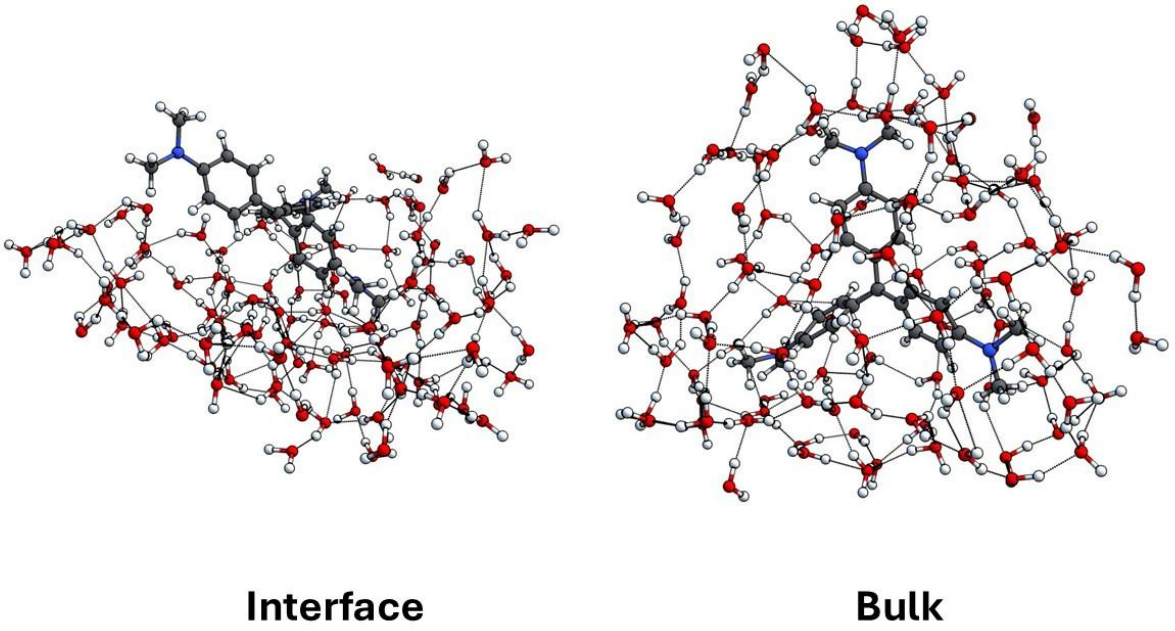

Figure S55. Representative geometries of TAM3 extracted from simulation at the air-water interface (left) and in bulk water (right), showing the dye surrounded by the 100 closest water molecules.
